# Discovery of VU6024578/BI02982816: An mGlu_1_ Positive Allosteric Modulator with Efficacy in Preclinical Antipsychotic and Cognition Models

**DOI:** 10.1021/acs.jmedchem.4c02554

**Published:** 2024-12-12

**Authors:** Carson W. Reed, Jacob F. Kalbfleisch, Jeremy A. Turkett, Trevor A. Trombley, Anthony F. Nastase, Paul K. Spearing, Daniel H. Haymer, Mohammad Moshin Sarwar, Marc Quitalig, Jonathan W. Dickerson, Annie L. Blobaum, Olivier Boutaud, Patrizia Voehringer, Niklas Schuelert, Hyekyung P. Cho, Colleen M. Niswender, Jerri M. Rook, Henning Priepke, Daniel Ursu, P. Jeffrey Conn, Bruce J. Melancon, Craig W. Lindsley

**Affiliations:** † Warren Center for Neuroscience Drug Discovery, Department of Pharmacology, Vanderbilt University, Nashville, Tennessee 37067, Unites States; ‡ Boehringer Ingelheim Pharma GmbH & Co. KG, Birkendorfer Str. 65, Biberach 88397, Germany

## Abstract

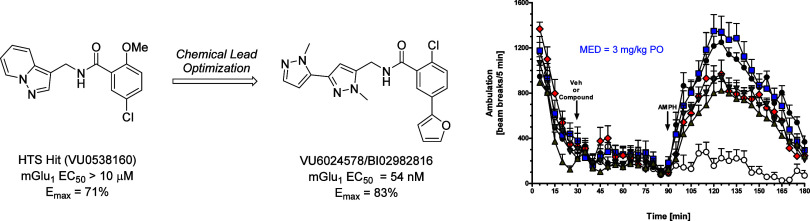

Herein, we report progress toward a metabotropic glutamate receptor subtype 1 (mGlu_1_) positive allosteric modulator (PAM) clinical candidate and the discovery of VU6024578/BI02982816. From a weak high-throughput screening hit (VU0538160, EC_50_ > 10 μM, 71% Glu_max_), optimization efforts improved functional potency over 185-fold to deliver the selective (inactive on mGlu_2–5,7,8_) and CNS penetrant (rat K_p_ = 0.99, K_p,uu_ = 0.82; MDCK-MDR1 ER = 1.7, P_app_ = 73 × 10–6 cm/s) mGlu_1_ PAM (VU6024578/BI02982816, EC_50_ = 54 nM, 83% Glu_max_). An excellent rat pharmacokinetic profile allowed the evaluation of VU6024578/BI02982816 in both amphetamine-induced hyperlocomotion (minimum effective dose (MED) = 3 mg/kg, p.o.) and MK-801 induced disruptions of novel object recognition (MED = 10 mg/kg p.o.), thus providing efficacy in preclinical models of psychosis and cognition. However, unanticipated AEs in dog prevented further consideration as a candidate. Thus, VU6024578/BI02982816 can serve as a best-in-class *in vivo* rodent tool to study selective mGlu_1_ activation.

## Introduction

The resurgence of interest in novel mechanisms and targets for the treatment of schizophrenia, especially in light of the clinical efficacy of activation of the muscarinic acetylcholine receptor 4 (M_4_) by agonists (KarXT (Cobenfy), an M_1_/M_4_ agonist),^1−3^ or PAMs (such as Cerevel’s emraclidine),^4^ has garnered significant attention. Recent work from our laboratories has demonstrated that the antipsychotic effects of M_4_ activation, and the ability to inhibit dopamine release, is dependent on coactivation of the metabotropic glutamate receptor subtype 1 (mGlu_1_). Importantly, the M_4_ PAM efficacy can be blocked by mGlu_1_ antagonists/negative allosteric modulators (NAMs).^5,6^ Previously, we demonstrated that mGlu_1_ PAM tool compounds inhibit dopamine release and exhibit antipsychotic-like effects in rodent models in an endocannabinoid dependent manner, akin to M_4_ PAMs.^6,-8^ Moreover, mGlu_1_ PAMs can restore cortical inhibitory tone and improve cognitive performance in a subchronic phencyclidine (PCP) NMDA hypofunction model.^6−8^ Recently, a human genetic link was also established.^9^ Multiple loss of function single nucleotide polymorphisms (SNPs) in the human gene encoding mGlu_1_ (*GRM1*) have been shown to be associated with schizophrenia, and *in vitro*, mGlu_1_ PAMs are capable of restoring mutant receptor signaling.^9^ We also hypothesized that positive allosteric modulation of mGlu_1_ would reduce cortical hyperactivity and re-establish the excitatory/inhibitory imbalance in the prefrontal cortex. Thus, mGlu_1_ PAMs represent a complementary approach to M_4_ PAMs for the treatment of, and an exciting new mechanism for, the positive symptoms and cognitive deficits of schizophrenia.

**Figure 1:**
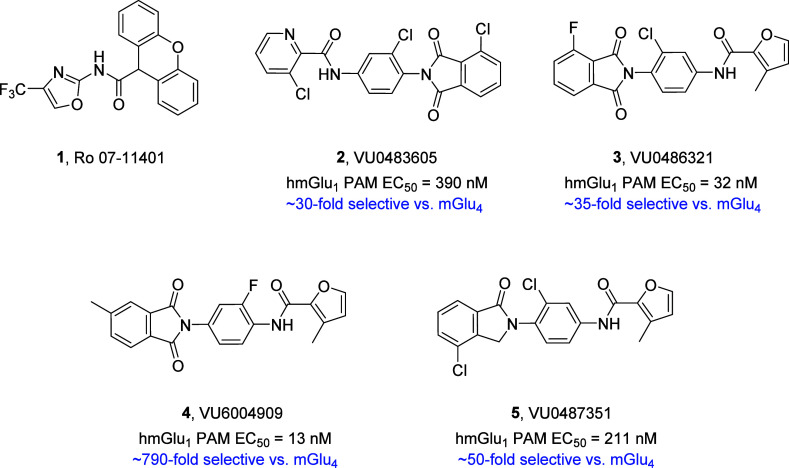
Structures of exemplar *in vitro* and *in vivo* mGlu_1_ PAM tool compounds **1**-**5**, highlighting the limited chemical diversity.

To date, the *in vitro* and *in vivo* mGlu_1_ PAM tools are limited in terms of potency, solubility, stability and pharmacokinetics (PK); however, they have provided target validation, despite not being suitable as leads for discovery campaigns (Figure 1).^6−19^ For example, second-generation tool compounds derived from a double “molecular switch” (a small chemical change that changes the mode of pharmacology) within a series of mGlu_4_ PAMs, afforded a critical antitarget, as well as hydrolytic instability of the phthalimide moiety and poor to modest CNS exposure.^9,14−19^ Driven by the exciting biology and therapeutic potential of mGlu_1_ PAMs,^5−9,14−19^ it was clear that the team would require new chemical matter to advance the program. Detailed here is the discovery effort leading to a new rodent *in vivo* mGlu_1_ PAM tool compound, VU6024578/BI02982816, with robust efficacy, high CNS exposure, an understanding of the pharmacokinetic/pharmacodynamic (PK/PD) relationship, as well as an unexpected liability in dog requiring further examination in other chemotypes.

## Results and Discussion

### High-Throughput Screening

**Figure 2:**
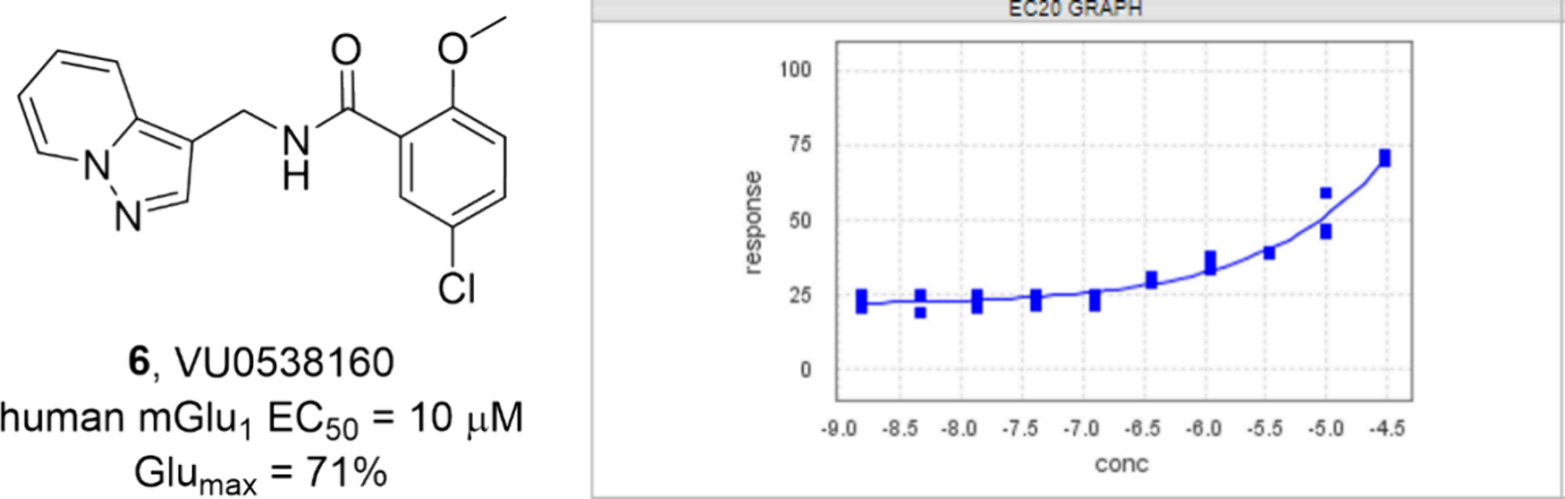
Structure of HTS hit **6** (VU0538160), a weak but chemically distinct mGlu_1_ PAM hit.

A stable cell line carrying tetracycline-inducible mGlu_1_ wildtype (WT) in T-REx-293 cells with a calcium fluorescent dye was miniaturized for the triple-add protocol for high-throughput screening of the Vanderbilt Collection (∼91,000 compounds) with a Z-prime score of 0.68.^20^ A single-point screen at a 10 μM concentration identified a number of mGlu_1_ agonists (176 primary hits), weak PAMs (1,574 primary hits) and antagonist/NAMs (435 primary hits). Through this screen, hit **6** (VU0538160), containing a pyrazolo[1,5-*a*]pyridine benzamide core (Figure 2), was confirmed to be a weak mGlu_1_ PAM (human mGlu_1_ EC_50_ > 10 μM; Glu_max_ = 71%). Due to it being chemically distinct from **1**-**5**, coupled with the ease of rapid hit expansion, PAM **6** was prioritized for further optimization.

### Hit Expansion and Chemical Optimization

**Scheme 1:**
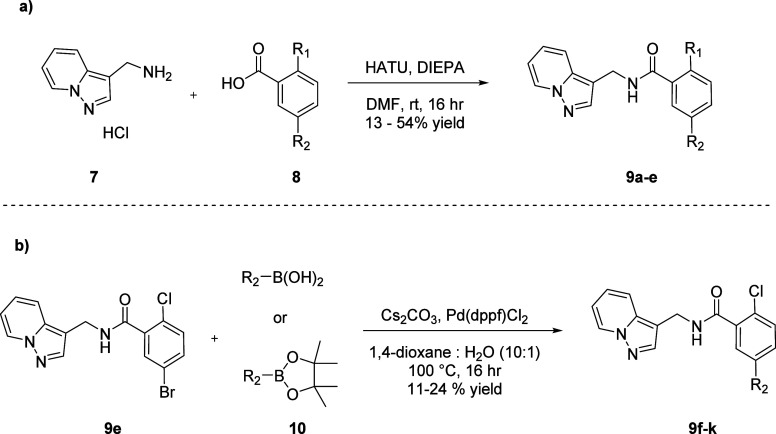
Synthesis of Analogs **9**

Derivatization of the eastern 2,5-disubstituted aromatic ring was explored first, and the synthesis of compounds of this nature is shown in Scheme 1.^20^ In the event, commercially available pyrazolo[1,5-a]pyridine-3-ylmethanamine **7** was reacted in a HATU-mediated amide coupling with benzoic acids **8** to afford analogs **9a**–**9d** in good to moderate yield (Scheme 1a). The SAR for select examples of analogs is described in Table 1. While it was observed that the presence of electron donating groups, such as OCH_3_ and CH_3_, at the 2-position (R_1_) of the eastern aromatic ring retained mGlu_1_ activity (compounds **9a** – **9c**), the incorporation of an electron withdrawing Cl atom (compound **9d**) led to an approximate 3-fold improvement in potency (human mGlu_1_ EC_50_ = 3.79 μM; Glu_max_ = 46%) compared to HTS hit **6**. Since the 5-position (R_2_) of the eastern aromatic ring tolerated a Cl atom, we wanted to further investigate the substitution at this position with various heterocycles. As shown in Scheme 1b,^20^ a Suzuki-Miyaura coupling between intermediate **9e** and the respective heteroaryl boronic acids or heteroaryl pinacol boronate esters afforded analogs **9f**-**9k**. The incorporation of a 2-thienyl at this position (compound **9g**) resulted in a drastic improvement in potency (human mGlu_1_ EC_50_ = 291 nM, Glu_max_ = 51%). However, the 3-thienyl congener (compound **9h**) was not as potent. This may indicate the necessity for the precise positioning of the southern heterocycle’s lone pair of electrons; therefore, additional heterocycles with heteroatoms at this 2-position were investigated. While both a 2-thiazole and a 5-*N*-methyl pyrazole heterocycle (compounds **9i** and **9j**) were not well tolerated, it was discovered that a 2-furanyl heterocycle (compound **9k**) was optimal. Compound **9k** displayed excellent human and rat mGlu_1_ potency (EC_50_s of 51 nM (53% Glu_max_) and 109 nM (92% Glu_max_), respectively) and moderate fraction unbound in both human (*f*_u_ = 0.036) and rat (*f*_u_ = 0.022) plasma and rat brain (*f*_u_ = 0.021). However, **9k** displayed high *in vitro* predicted hepatic clearance in both human (CL_hep_ = 19.8 mL/min/kg) and rat (CL_hep_ = 63.7 mL/min/kg). Additionally, in a rat PK PBL experiment, **9k** was found to have high *in vivo* clearance (CL_p_ = 67.2 mL/min/kg), a short half-life (*t*_1/2_ = 23.8 min), a short mean residence time (MRT = 21.3 min), and modest CNS penetrance (K_p_ = 0.27). With these data, it was clear that further optimization of these physiochemical properties was needed before selecting a suitable candidate as an *in vivo* mGlu_1_ tool compound.

**Table 1:**
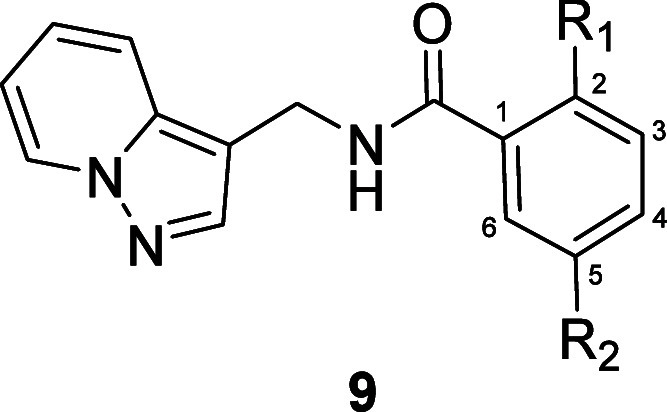

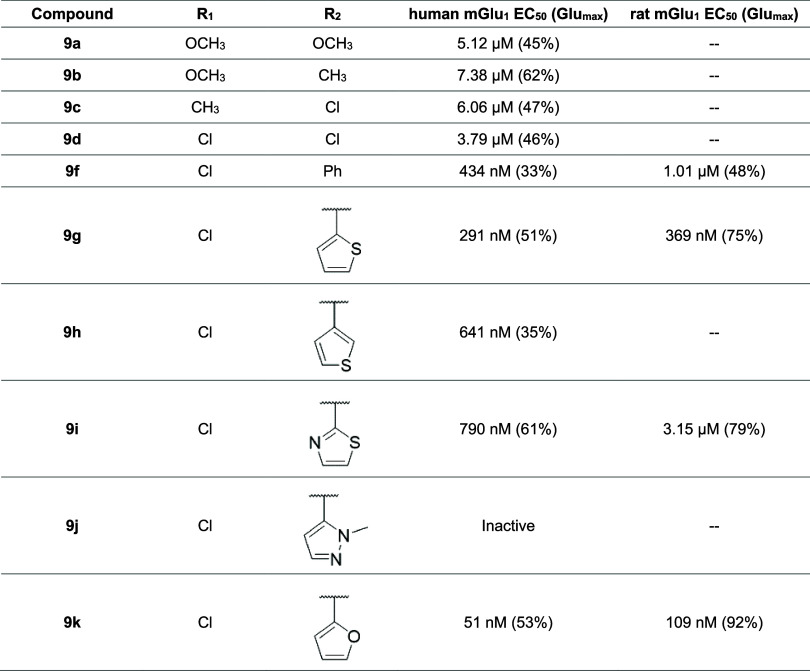
SAR of Select Analogs **9**

**Scheme 2:**
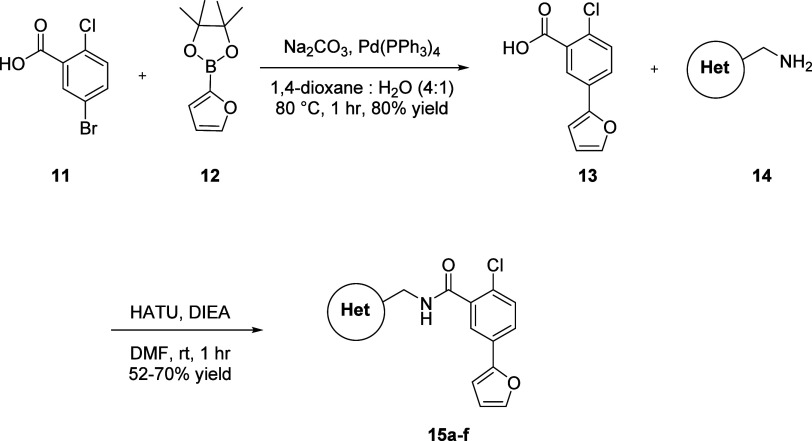
Synthesis of Analogs **15a**-**f**

With the optimal eastern heterobiaryl group in hand, the western portion of the scaffold was next investigated to see if the pyrazolopyridine heterocycle could be replaced with another heterocyclic motif. In order to access analogs of this nature, 2-chloro-5-bromobenzoic acid **11** was reacted with pinacol boronate ester **12** in a Suzuki-Miyaura coupling to afford benzoic acid **13** in 80% isolated yield. A subsequent HATU-mediated amide coupling with heterocyclic methanamines **14** delivered diverse analogs **15** in yields ranging from 52 to 70% (Scheme 2).^20^ The SAR for select examples of analogs **9** and their *in vitro* DMPK profiles are described in Table 2 and Table 3, respectively.

**Table 2:**
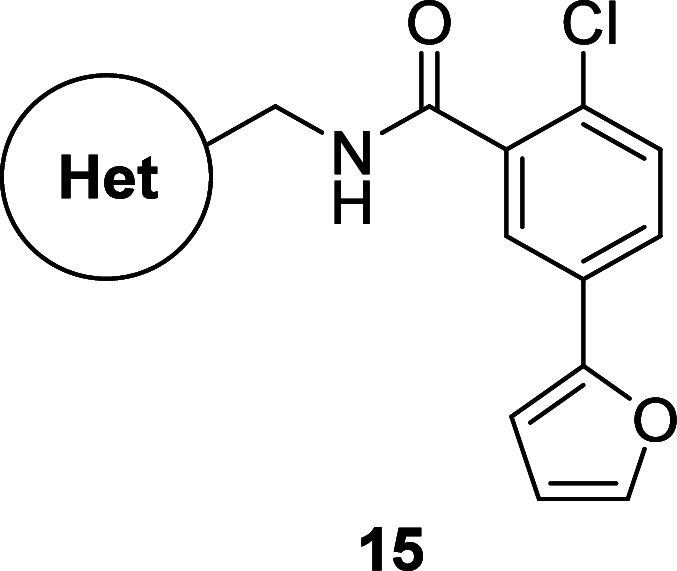

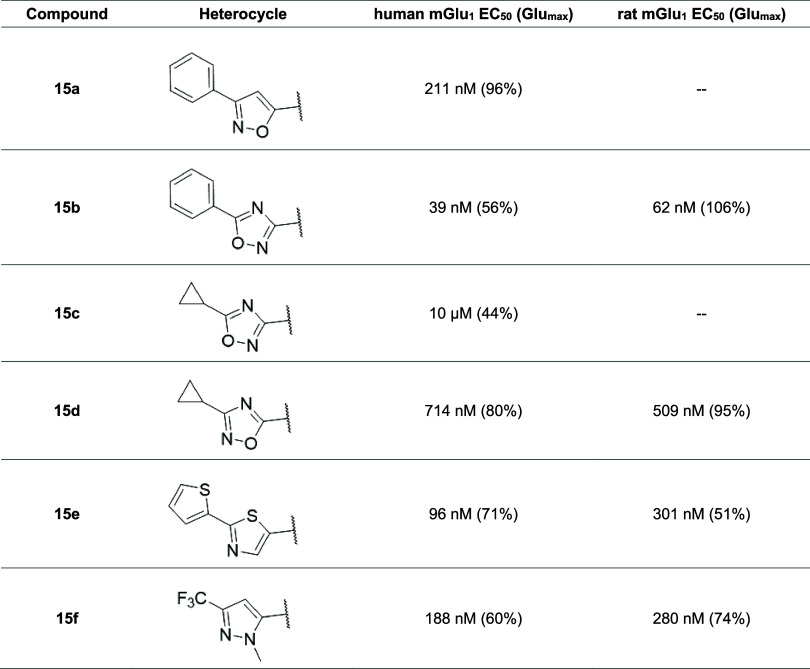
SAR for Select Examples of Analogs **15**

**Table 3:** *In Vitro* DMPK Profiles for Analogs **15**

Compound	CL_hep_ (mL/min/kg), human	CL_hep_ (mL/min/kg), rat	human *f*u_plasma_	rat *f*u_plasma_	rat *f*u_brain_
**15a**	19.4	60.9	0.001	0.009	--
**15b**	19.9	61.8	0.001	0.004	0.005
**15e**	19.7	57.4	0.001	0.003	0.002
**15f**	20.3	57.1	0.036	0.025	0.019

Gratifyingly, additional aryl-, heteroaryl-, and aliphatic-substituted heterocycles on the western portion of the molecule were tolerated. In particular, isoxazole, 1,2,4-oxadiazole, and thiazole motifs (as seen in compounds **15a**, **15b**, **15d**, and **15e**) maintained good to moderate mGlu_1_ potency. However, further investigation of their *in vitro* DMPK profiles revealed these compounds to have high predicted hepatic clearance in both human and rat, in addition to low fraction unbound in both human and rat plasma and rat brain. Interestingly, compound **15f**, containing a central *N*-Me pyrazole heterocycle, displayed moderate human and rat mGlu_1_ potency (188 nM, 60% Glu_max_ and 280 nM, 74% Glu_max_, respectively) and moderate fraction unbound in both human (*f*_u_ = 0.036) and rat (*f*_u_ = 0.025) plasma and rat brain (*f*_u_ = 0.019), albeit with high predicted hepatic clearance in human (CL_hep_ = 20.3 mL/min/kg) and rat (CL_hep_ = 57.1 mL/min/kg).

**Scheme 3:**
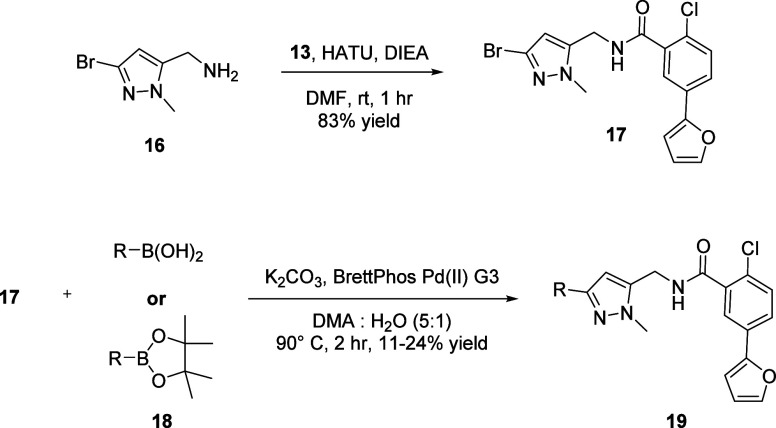
Synthesis of Analogs **19**

Given the relative improvement in plasma protein and brain homogenate binding for compound **15f**, additional SAR around this central *N*-Me pyrazole heterocycle was warranted. The synthesis of analogs exploring this SAR is outlined below in Scheme 3. From known methanamine **16**, a HATU-mediated amide coupling with benzoic acid **13** resulted in the formation of amide **17** which was then subjected to Suzuki-Miyaura couplings with various heteroaryl boronic acids or heteroaryl pinacol boronate esters **18** to afford analogs **19**.^20^

Both the SAR describing analogs **12** and their *in vitro* DMPK profiles are described in Table 4 and Table 5, respectively. While the incorporation of lipophilic substituents, such as a 2-thienyl (compound **19a**) and a 2-fluorophenyl (compound **19b**), were well tolerated from a potency perspective, it was clear that these lipophilic substituents were detrimental to plasma protein and brain homogenate binding. The introduction of a polar 3-pyridyl substituent (compound **19c**) displayed excellent human and rat mGlu_1_ potencies (EC_50_ of 74 nM (60% Glu_max_) and 124 nM (116% Glu_max_), respectively) with moderate fraction unbound in both human (*f*_u_ = 0.023) and rat (*f*_u_ = 0.015) plasma and rat brain (*f*_u_ = 0.021). We were pleased to note that the incorporation of a 5-*N*-Me pyrazole substituent (compound **19d**) also displayed excellent human and rat mGlu_1_ potencies (EC_50_ of 54 nM (83% Glu_max_) and 46 nM (124% Glu_max_), respectively) with improved fraction unbound in both human (*f*_u_ = 0.03) and rat (*f*_u_ = 0.041) plasma and rat brain (*f*_u_ = 0.034). Additionally, compound **19d** displayed moderate predicted hepatic clearance in both human (15.8 mL/min/kg) and rat (39.3 mL/min/kg). In an *in vivo* rat PK PBL experiment, compound **19d** was found to have low clearance (8.3 mL/min/kg), moderate half-life (*t*_1/2_ = 3.6 h), moderate mean residence time (MRT = 3.5 h), good volume of distribution (V_ss_ = 1.78 L/kg), and good CNS penetrance (K_p_ = 0.99 and K_p,uu_ = 0.82).^20^ Given these data, compound **19d** was selected as a suitable *in vivo* tool compound to further explore the *in vivo* pharmacology of mGlu_1_.

**Table 4:**
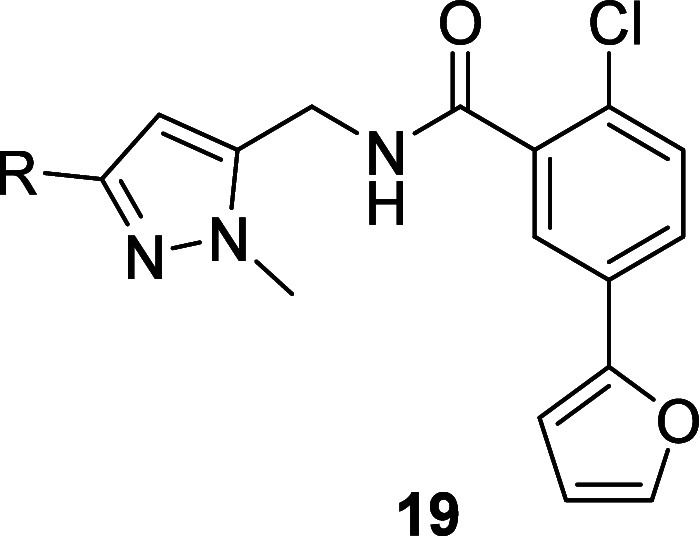

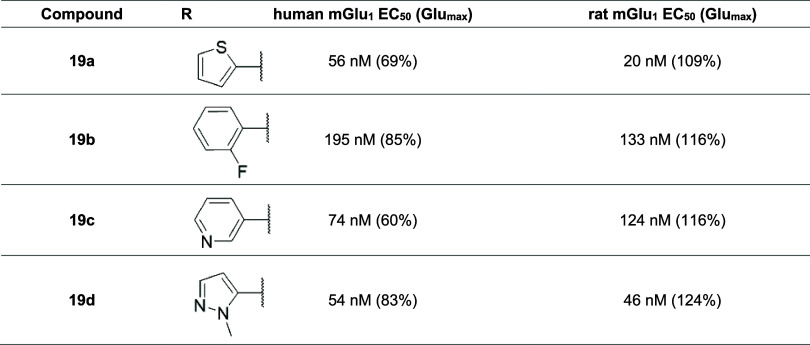
SAR for Select Analogs **19**

**Table 5:** *In Vitro* DMPK Profiles for Analogs **19**

Compound	CL_hep_ (mL/min/kg), human	CL_hep_ (mL/min/kg), rat	human *f*u_plasma_	rat *f*u_plasma_	rat *f*u_brain_
**19a**	19.0	54.8	0.002	0.002	0.003
**19b**	18.6	56.4	0.002	0.002	0.003
**19c**	18.8	51.7	0.023	0.015	0.021
**19d**	15.8	39.3	0.030	0.041	0.034

**Scheme 4:**
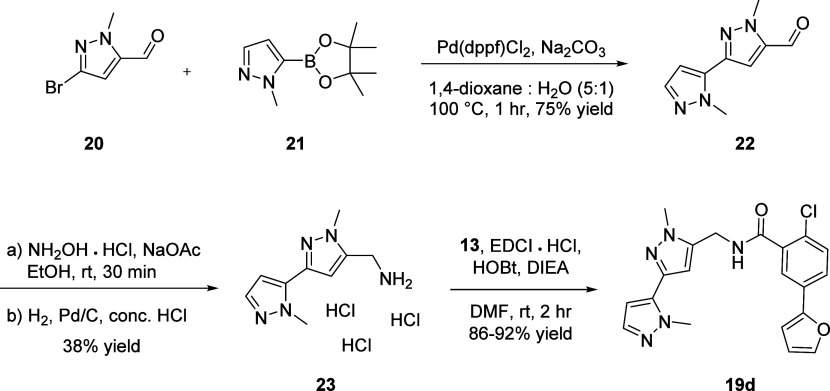
Optimized Synthesis of **19d**

While the initial synthetic route was suitable for making milligram quantities of **19d**, we wanted to optimize this compound’s synthesis in order to access gram scale quantities to enable *in vivo* behavioral work. Scheme 4 outlines this optimized synthetic route. Known aldehyde **20** and pinacol boronate ester **21** were reacted in a Suzuki-Miyaura coupling to afford aldehyde **22** in 75% yield. Oxime formation followed by subsequent reduction under acidic conditions yielded methanamine **23** as the tris-HCl salt. **23** was then coupled to benzoic acid **13** under EDCI-HOBt conditions to afford **19d** (VU6024578/BI02982816) in excellent yields (86–92%).^20^

### Molecular Pharmacology

**Figure 3:**
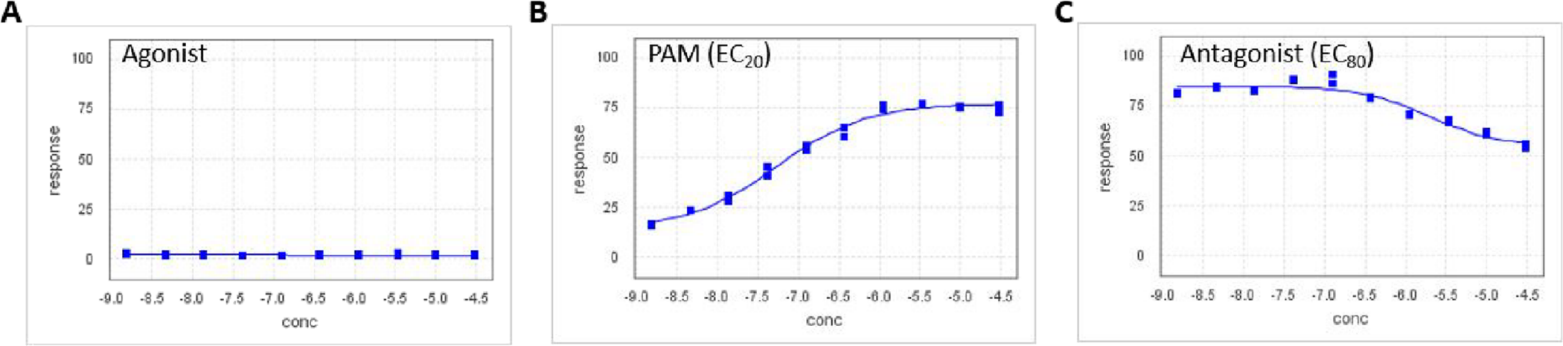
Triple-Add primary mGlu_1_ assay CRCs of **19d**. A) The agonist (or compound alone window) indicating no intrinsic agonist activity. B) The PAM (EC_20_) window, showing a robust PAM CRC. C) The antagonist (EC_80_) window, displaying little receptor desensitization.

PAM **19d** (VU6024578/BI02982816) was a low molecular weight (395.4) compound with acceptable lipophilicity (xLogP = 3.9) and low solubility (kinetic solubility at pH 7.4 = 4.1 μM; FaSSIF (2.5 μM) and FaSSGF (2.5 μM)). Over the course of the mGlu_1_ PAM program, **19d** became an assay standard and was assayed multiple times, affording refined potency values.^20^ On human mGlu_1_, **19d** displayed an EC_50_ of 54 nM (83% Glu_max_) [n = 77] (pEC_50_ = 7.31 ± 0.02) and was comparably potent on rat mGlu_1_ (EC_50_ = 46 nM (124% Glu_max_) [n = 110] (pEC_50_ = 7.39 ± 0.02)). Importantly, **19d** was inactive (>30 μM) against human and rat mGlu_2–5,7,8_, further differentiating away from the mGlu_4_ activity of predecessor mGlu_1_ PAMs **2**-**5**. PAM activity was also maintained across species, with mouse (EC_50_ = 27 nM (89% Glu_max_) [n = 5] (pEC_50_ = 7.59 ± 0.07)) and dog (EC_50_ = 87 nM (129% Glu_max_) [n = 3] (pEC_50_ = 7.07 ± 0.07)) being in-line with human and rat mGlu_1_ PAM activity. Another consideration for an mGlu_1_ PAM *in vivo* tool was a lack of intrinsic agonist activity, as well as desensitization, since mGlu_5_ ago-PAMs possessed significant neurotoxicity (as these are both Group I mGlu receptors).^21^ As shown in Figure 3, observing calcium fluorescence as a surrogate for mGlu_1_ receptor activity in our triple-add protocol,^20^**19d** displays no agonism (Figure 3A) up to 30 μM compound concentration, but affords a robust PAM concentration response curve in the presence of a subthreshold (EC_20_) concentration of glutamate (Figure 3B). Interestingly, in the antagonist or EC_80_ add window (Figure 3C), there is only slight mGlu_1_ desensitization. Overall, **19d** represented an ∼185-fold improvement in mGlu_1_ PAM activity over the HTS hit **6** and demonstrates once again that weak GPCR PAM hits can be readily optimized.

**Figure 4:**
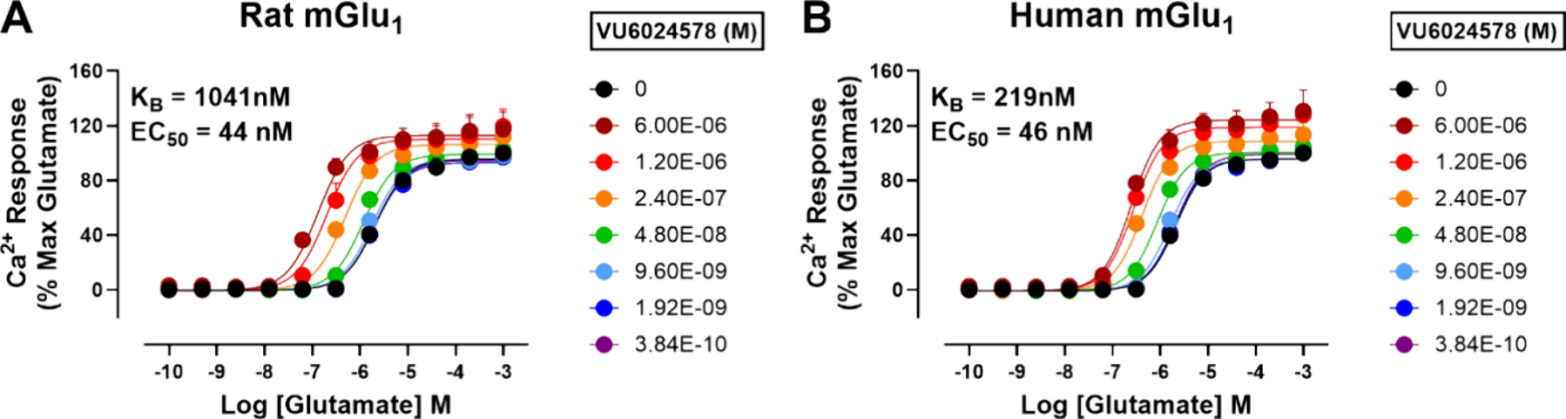
Operational modeling of the effects of increasing concentrations of VU6024578 (**19d**) on rat (A) and human (B) mGlu_1_receptors. Increasing amounts of **19d** were applied prior to increasing concentrations of glutamate at either rat or human mGlu_1_. Data were fit in GraphPad Prism as indicated in the Methods section; for panel A, log α was 0.95 and for panel B it was 1.26. Data represent one experiment performed in duplicate.

To further characterize this novel mGlu_1_ PAM, we performed progressive fold-shift assays at both human and rat mGlu_1_ (Figure 4). PAM **19d** afforded a maximal left-ward fold-shift of the glutamate concentration–response curve (CRC) at 6 μM of 8.7-fold for human and 18.4-fold for rat. The operational model of allosterism was applied to the fold-shift data to better understand the estimated binding affinity (K_B_) of **19d**. From these data, the estimated K_B_ for human mGlu_1_ was 219 nM and 1.04 μM for rat.^20^

Ancillary pharmacology in a Eurofins Lead Profiling screen of 80 GPCRs, ion channels and transporters was relatively clean (no displacement of radioligands >50% @ 10 μM) except for BZD (peripheral antagonist radioligand) at 95%, EP4 (agonist radioligand) at 62%, 5-HT1A (agonist radioligand) at 51%, and 5-HT_2B_ (agonist radioligand) at 67% (see Supporting Information Table S1).^20^ In follow-up functional assays, **19d** was inactive at 5-HT_2B_, but a functional antagonist at the peripheral BZD (IC_50_ = 207 nM). As BZD had never before been a hit in our programs, we paused to explore this off-target. BZD, or peripheral benzodiazepine receptor (PBR), is now referred to as translocator protein, expressed in peripheral tissues and viewed as an orphan protein.^22,23^ Since the ancillary pharmacology was at a peripheral target, we did not feel this activity would confound central mGlu_1_, thus providing a clean profile for more definitive proof-of-concept studies to validate selective mGlu_1_ activation in preclinical models of psychosis and cognition. In a hERG manual patch clamp assay, **19d** displayed an IC_50_ > 10 μM and was negative in an Ames test (4-strain ± S9).^20^ Thus, **19d** was suitable for advancement into more in-depth *in vitro* and *in vivo* DMPK profiling.

### *In vitro* DMPK of **19d**

**Figure 5:**
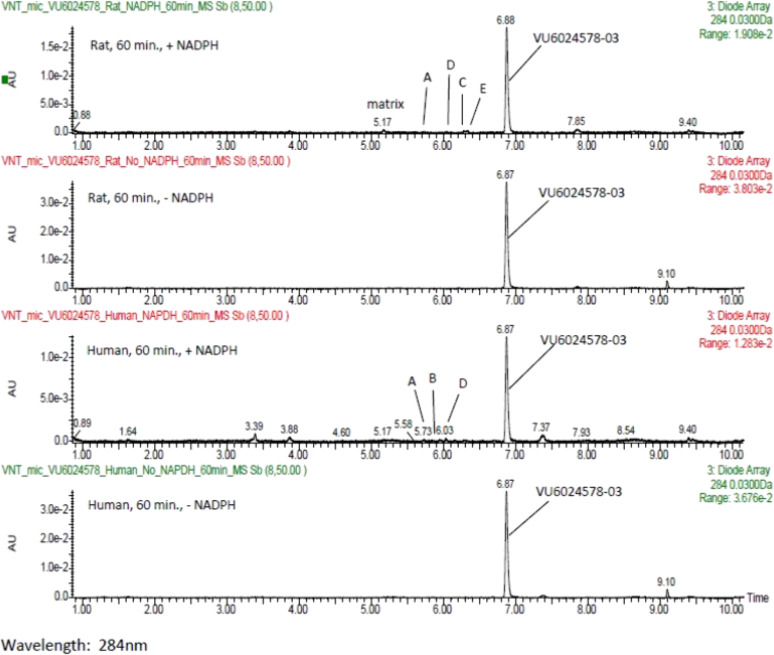
Metabolite identification of VU6024578 (**19**d) in rat and human liver microsomes. VU6024578–03 (**19d**) was the major component observed in the rat and human 60 min samples (with and without NADPH) based on UV peak areas (as well as by extracted ion). Thus, we incubated **19d** in rat and human liver microsomes (with and without NADPH) for 60 min and monitored by both extracted ion and UV detection for the production of metabolites and consumption of parent.

Unlike the phthalimide moiety in **2**-**5**, **19d** was hydrolytically stable under both acidic (pH 2.2) and neutral (pH 7.4) conditions.^14−19^ Across species, **19d** had acceptable fraction unbound in both plasma (*f*_u_ (plasma): 0.03 (human), 0.041 (rat), 0.025 (cyno), 0.039 (dog) and 0.056 (mouse)) and brain (*f*_u_ (brain): 0.034 (rat) and 0.081 (mouse)). Predicted hepatic clearance was moderate across species (CL_hep_ (h, r, d, c) = 16, 39, 23, and 31 mL/min/kg, respectively) and **19d** was not a human P-gp substrate (MDCK-MDR1 ER = 1.7 (P_app_ = 73 × 10^–6^ cm/s). PAM **19d** had an acceptable CYP450 profile (3A4:22 μM; 2D6:28 μM; 2C9:6.4 μM; 1A2:17 μM) and no 3A4 mechanism-based inhibition.^20^ We were concerned about the potential liability of the furanyl moiety, and its propensity to be metabolized to reactive intermediates, as it could be a toxicophore. Fortunately, **19d** proved to be extremely stable in these preparations and no metabolites corresponding to oxidation or opening of the furanyl ring were detected (Figure 5).^20^ A follow-up *in vitro* study to determine if reactive intermediates were produced by **19d** in rat and human liver microsomes with glutathione identified a number of minor glutathione conjugates with four involving the furanyl ring (see Supporting Information Figure S1).^20^ Thus, **19d** had a blemish, but was not out of contention as a development candidate; moreover, **19d** was clearly highly valuable as an *in vivo* rodent tool compound for definitive target validation.

### *In vivo* DMPK of **19d**

In a rat IV (1 mg/kg IV, 10% EtOH, 40% PEG, 50% saline)/PO (10% tween 80 in water) PK crossover study, **19d** displayed a highly favorable profile with a plasma clearance (CL_p_) of 8.3 mL/min/kg, a *t*_1/2_ of 3.9 h, a V_ss_ of 1.78 L/kg and 100% oral bioavailability (%F).^20^ Thus, **19d** was a stable chemical entity *in vivo*. In the PO arm, the *T*_max_ was 2 h with a *C*_max_ of 4150 ng/mL and an AUC of 24599 ng/mL. In our standard rat IV plasma:brain level (PBL) cassette paradigm (0.25 mg/kg/compound 15 min time point), PAM **19d** showed good CNS penetrance (K_p_ = 0.99 and K_p,uu_ = 0.82), and in-line with the *in vitro* P-gp data. We next performed a PO PBL study at 3 mg/kg in 10% tween 80 and found a K_p_ of ∼0.5 across time points 15 min to 7 h, resulting in an unbound brain concentration Br_[unbound]_ of 100 nM (∼2.2-fold the rat EC_50_) at *T*_max_. In an analogous IP PBL study at 3 mg/kg, Br_[unbound]_ of 68 nM (∼1.5-fold the rat EC_50_) at *T*_max_ was achieved.^20^ Overall, the rat PK and CNS penetration was supportive of *in vivo* work and progression down the development flow-chart.

### Behavioral Pharmacology of **19d**

In order to enable our target validation work, we first profiled PAM **19d** in a rat locomotor battery to ensure that pharmacodynamic efficacy was not mistaken for gross effects on locomotion.^20^ In a standard catalepsy assay measuring latency to withdraw, the positive control haloperidol induced profound catalepsy; in contrast, doses of **19d** up to 10 mg/kg were without effect 100 min postdose. A similar lack of effect was noted in both rotarod and spontaneous locomotion assays.^20^ PK was performed in parallel to establish a PK/PD relationship.^24^ In these studies, the K_p_ of **19d** was ∼0.75 with free brain concentration at 10 mg/kg of 134 nM (∼2.9-fold over the *in vitro* EC_50_), thus mGlu_1_ is sufficiently activated (see Supporting Information Figure S2).^20^

**Figure 6:**
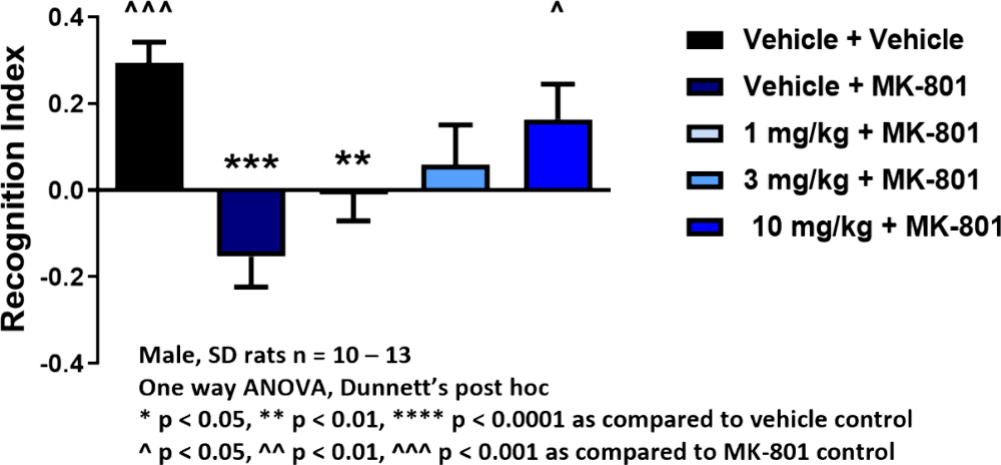
Rat MK-801 induced disruption of novel object recognition and reversal by VU6024578 (**19d**). MK-801 (0.5 mg/kg IP) induced a robust disruption of NOR, which was dose-dependently reversed by oral administration (10% tween 80) of **19d**.

Based on our previous work with the mGlu_1_ PAM **4**,^6−9,14−19^ we first evaluated the ability of **19d** to reverse MK-801 disruptions of novel object recognition (NOR) in rats (Figure 6). As shown in Figure 6, MK-801 administration robustly disrupts NOR in rats, and this pharmacological disruption is dose-dependently reversed by oral administration of **19d**, with a minimum effective dose (MED) at 10 mg/kg. Satellite PK (PO, 210 min postdose to mirror testing window in the NOR) with **19d** demonstrated a K_p_ of 0.34 with free brain concentration at 10 mg/kg of 73 nM (∼1.4-fold over the *in vitro* EC_50_), thus mGlu_1_ has sufficient target coverage and PK/PD is established for this paradigm (see Supporting Information Table S2).^20^

**Figure 7:**
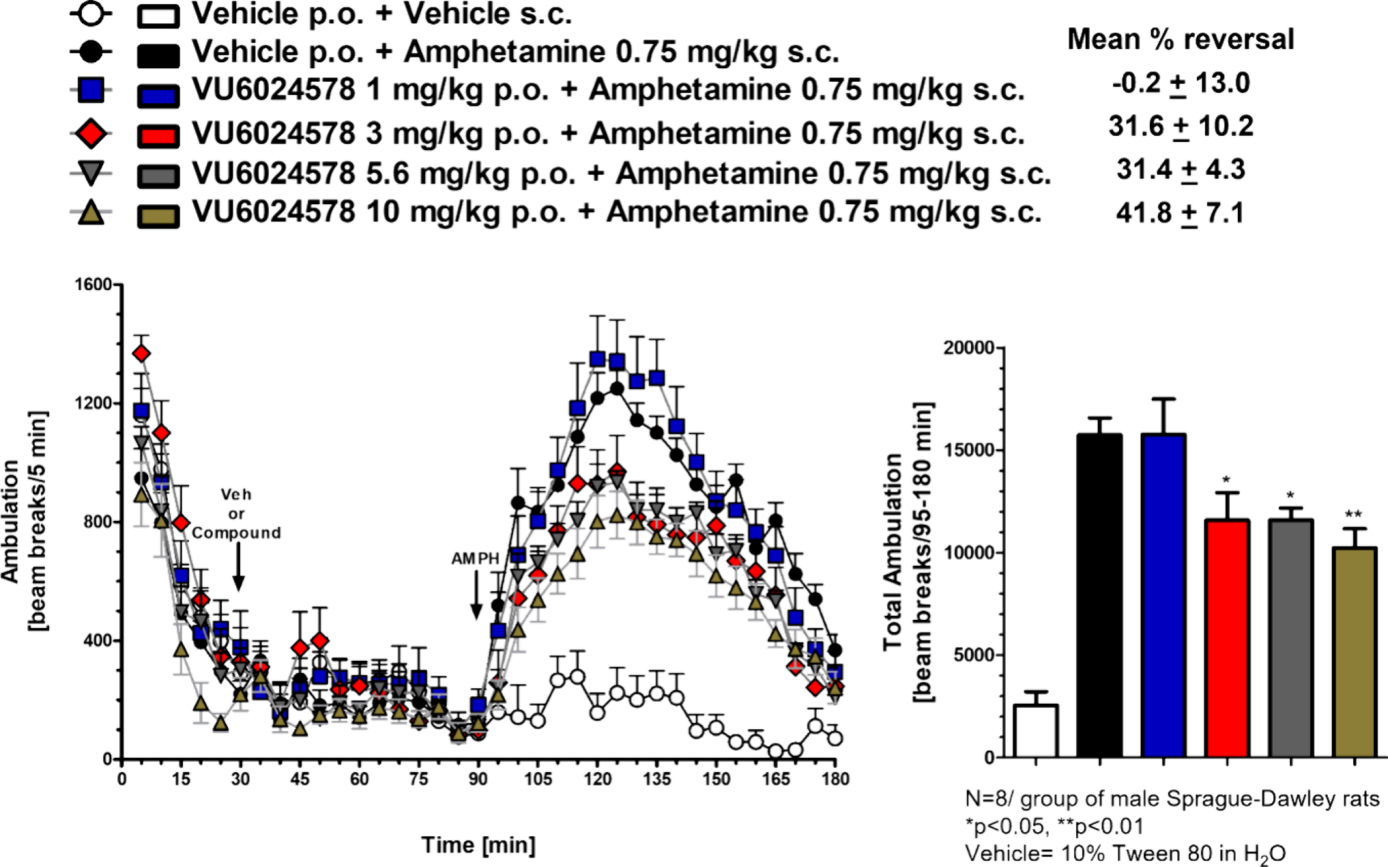
Rat amphetamine-induced hyperlocomotion and reversal by VU6024578 (**19d**). Amphetamine (0.75 mg/kg IP) induced robust hyperlocomotion, which was dose-dependently reversed by oral administration (10% tween 80) of **19d**.

Based on the lack of effect in the rat locomotor panel, we were confident we could assess the efficacy of **19d** on reversing amphetamine-induced hyperlocomotion (AHL), a standard preclinical psychosis model where clinically available antipsychotics, and M_4_ PAMs, display robust efficacy (Figure 7).^5^ Here, administration of 0.75 mg/kg IP of amphetamine induced a robust hyperlocomotive state in rats (>1200 beam breaks), which was dose-dependently reversed by oral administration of **19d**. Once again, satellite rat PK taken at the 2.5-h end of study time point showed that at the 3 mg/kg MED, there was a free brain concentration of 32 nM (∼0.6-fold over the *in vitro* EC_50_) and maximal reversal at 10 mg/kg (free brain concentration of 67 nM, ∼ 1.2-fold over the *in vitro* EC_50_) (see Supporting Information Table S3).^20^ Considering the longer time point for exposure and the results, mGlu_1_ has sufficient target coverage and PK/PD is established for this paradigm.^24^

**Figure 8:**
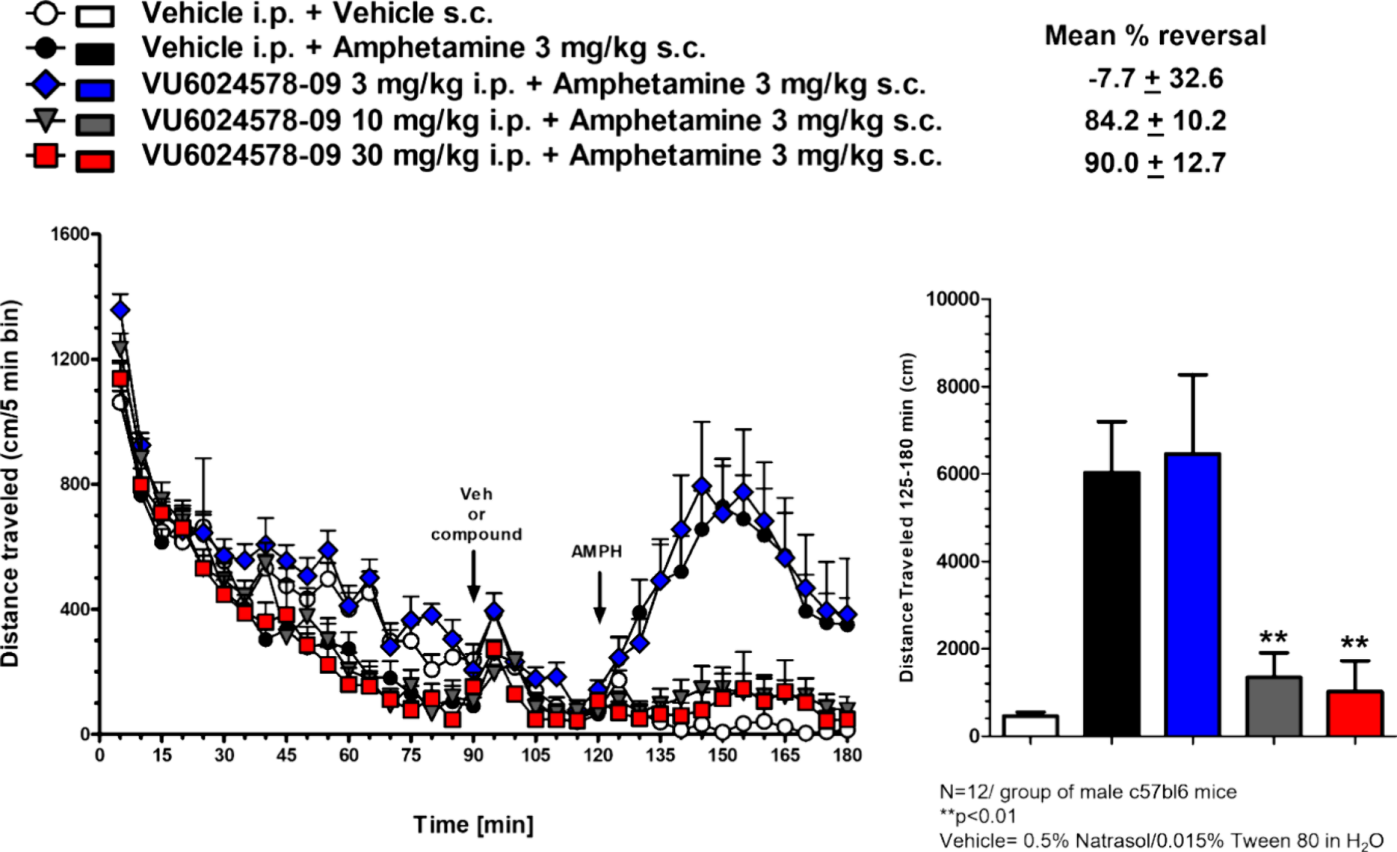
Mouse amphetamine-induced hyperlocomotion and reversal by VU6024578 (**19d**). Amphetamine (3.0 mg/kg s.c.) induced robust hyperlocomotion, which was dose-dependently reversed by oral administration (0.5% Natrasol/0.015% tween 80 in H_2_O) of **19d**.

In parallel with the rat AHL study, we carried out a mouse AHL study to determine if the PK/PD relationship would remain intact across rodent species. Here, amphetamine was dosed to C57Bl/6 mice at 3 mg/kg s.c. to achieve ∼800 beam breaks, followed by the oral administration of **19d** at 3, 10, and 30 mg/kg (Figure 8). At 3 mg/kg, there was no reversal of AHL, but free brain levels in the satellite PK study only reached 7 nM (far below the mouse EC_50_ of 27 nM). Full reversal was observed at both the 10 and 30 mg/kg doses, where free brain concentrations were 36 nM (1.3-fold over the EC_50_) and 67 nM (2.5-fold over the EC_50_), respectively (see Supporting Information Table S4).^20^ Thus, mGlu_1_ has sufficient target coverage and PK/PD^24^ is established for this paradigm in mouse and agree well with the PK/PD relationship established in rat for both NOR and AHL wherein free brain concentrations at or above the *in vitro* EC_50_ facilitates a significant pharmacodynamic response.

**Figure 9:**
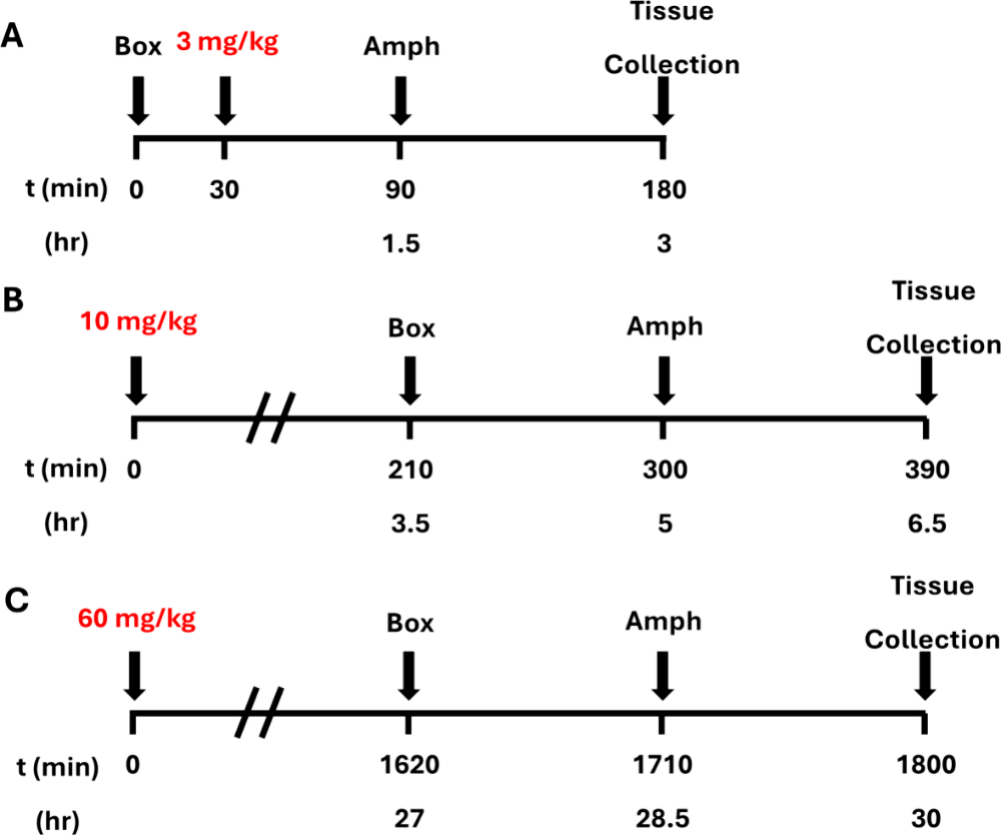
Study design for Amphetamine-induced hyperlocomotion *C*_max_ versus C_trough_ study.

Based on these positive behavioral data and the clear PK/PD relationship tied to achieving free brain levels at, or above, the mGlu_1_ PAM EC_50_, we next wanted to define the role of *C*_max_ versus C_trough_ drug (**19d**) concentrations in driving the PK/PD relationship. To accomplish this, pretreatment times and doses were calculated based upon previous PK (see Supporting Information Figure S3)^20^ data to target free brain concentrations at and below the *in vitro* EC_50_ at the time of amphetamine challenge. Rats were pretreated with 3 mg/kg **19d** or vehicle after habituation to the locomotor chambers are described in Figure 9A. Rats pretreated with **19d** (10–60 mg/kg) or vehicle were administered compound prior to starting the amphetamine-induced hyperlocomotion experiment as depicted in Figure 9**B-D**. Ambulations were evaluated for 90 min post amphetamine treatment for all dosing groups. Pretreatment of **19d**, chamber habituation, amphetamine treatment and ambulation recording times are outlined for each dosing group in Figure 9. Blood and brain samples were obtained at the end of testing (EOT) to determine **19d** levels at the end of the study (Table 6).

**Table 6:** Terminal Unbound Brain Concentrations of **19d** Following Rat AHL

Measured conc. [ nM]		
Dose (mg/kg)	Pretreatment Time (hr)	Brain Unbound (nM) @EOT
3	2.5	40
10	6.5	44
60	15	3
60	30	6

**Figure 10:**
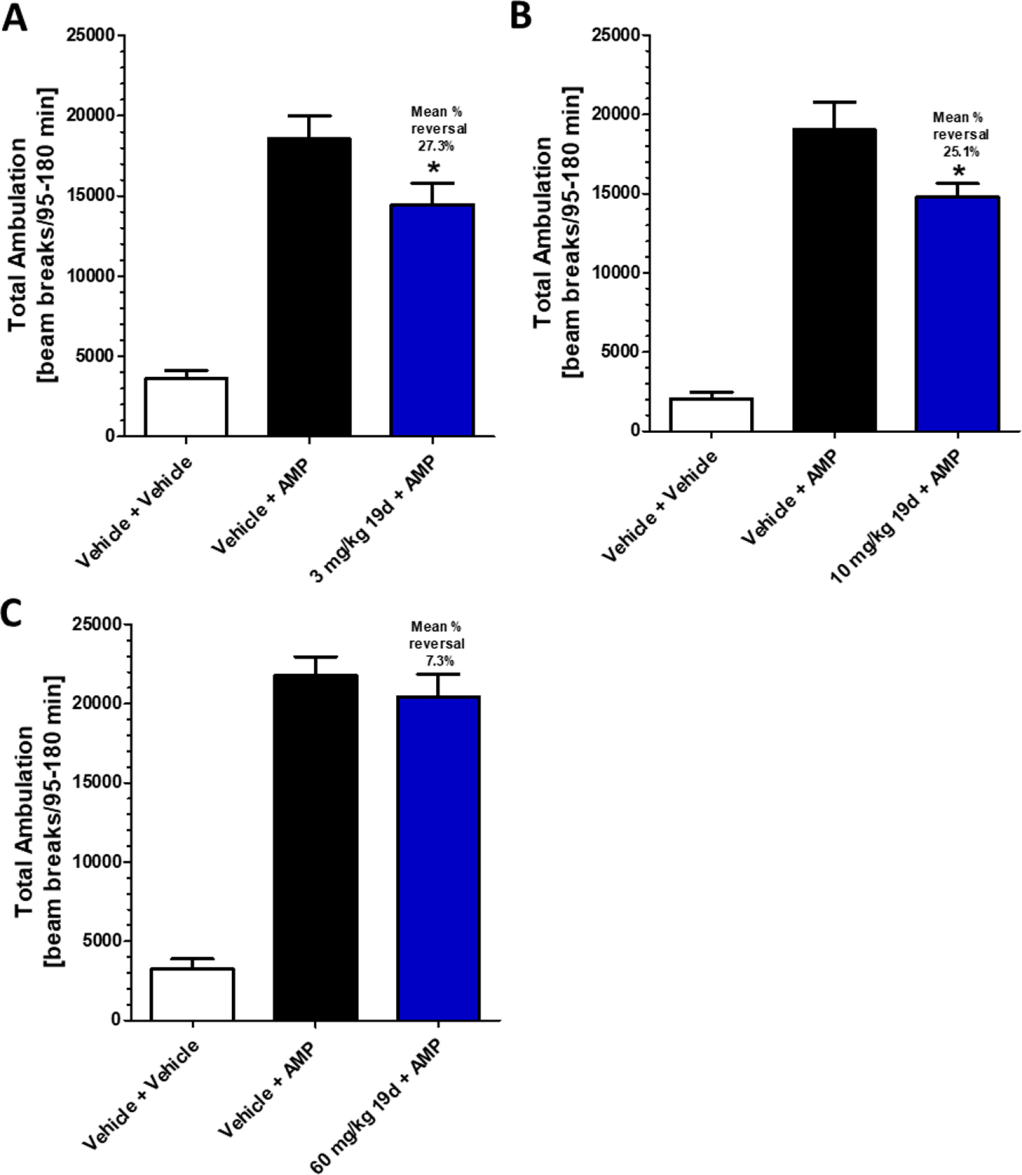
Amphetamine-induced hyperlocomotion in rats. A) 1 h pretreatment of 3 mg/kg **19d**. B) 5 h pretreatment of 10 mg/kg **19d**. C) 28.5 h pretreatment of 60 mg/kg **19d**. Total ambulation from 95 to 180 min. *p < 0.05 compared to Vehicle + Amphetamine treated animals.

The administration of amphetamine significantly increased the locomotor activity of the rats in this experiment compared to vehicle treated animals in all treatment groups. Additionally, pretreatment with 3 mg/kg of **19d** 1 h prior to amphetamine significantly reduced the amphetamine-induced hyperlocomotion in this experiment (Figure 10A). The terminal free brain concentrations for this dosing group were 40 nM. Similarly, pretreatment with 10 mg/kg of **19d** 5 h before amphetamine administration significantly reduced the amphetamine-induced hyperlocomotion in this experiment (Figure 10B), and free brain concentrations at the end of the study were 44 nM. Pretreatment with 60 mg/kg of **19d** resulted in a *C*_max_ of 313 nM at 3.33 h post administration. This concentration is 6.8-fold over the rat *in vitro* potency of the compound at *T*_max_ and well above what we find is required for *in vivo* efficacy in AHL. Therefore, based upon these data and PK modeling, we chose a pretreatment time for **19d** of 28.5 h before amphetamine to determine if efficacy would still be observed after brain levels had fallen below our *in vitro* potency The reduction of locomotor activity after amphetamine was not demonstrated in animals dosed 60 mg/kg of VU6024578 28.5 h before amphetamine (Figure 10C) where the brain levels were significantly lower at the end of the AHL study (6 nM) compared to the other dosing groups. For the 3 and 10 mg/kg dosing paradigms that maintained brain concentrations similar to the EC_50_ of the compound and those observed in previous NOR and AHL studies in rats and mice that demonstrated efficacy, similar reduction in locomotor activity after amphetamine were observed. However, the dosing paradigm of 60 mg/kg with a pretreatment time that resulted in brain concentrations significantly lower than the potency of the compound at the time of testing did not achieve efficacy in this model. These data suggest that regardless of the maximum concentration achieved in the brain, the free brain concentrations present during the amphetamine challenge (C_trough_) are the driving factor for the observed antipsychotic-like activity in AHL.^20^

### Translatable Biomarker Efforts

The evaluation of potential therapeutic compounds often necessitates the use of robust and reliable biomarkers that can provide insights into the drug’s effect on neurophysiological functions. In this context, we have followed two approaches to investigate the positive modulation of mGlu1 receptors: (i) quantitative analysis of electroencephalography signals (qEEG)^25^ and (ii) auditory event-related potentials (ERP).^26^ qEEG, including EEG power spectra and vigilance states analyses are recognized as useful physiological and translational biomarkers for the investigation of mental disorders.^20,25^

**Figure 11:**
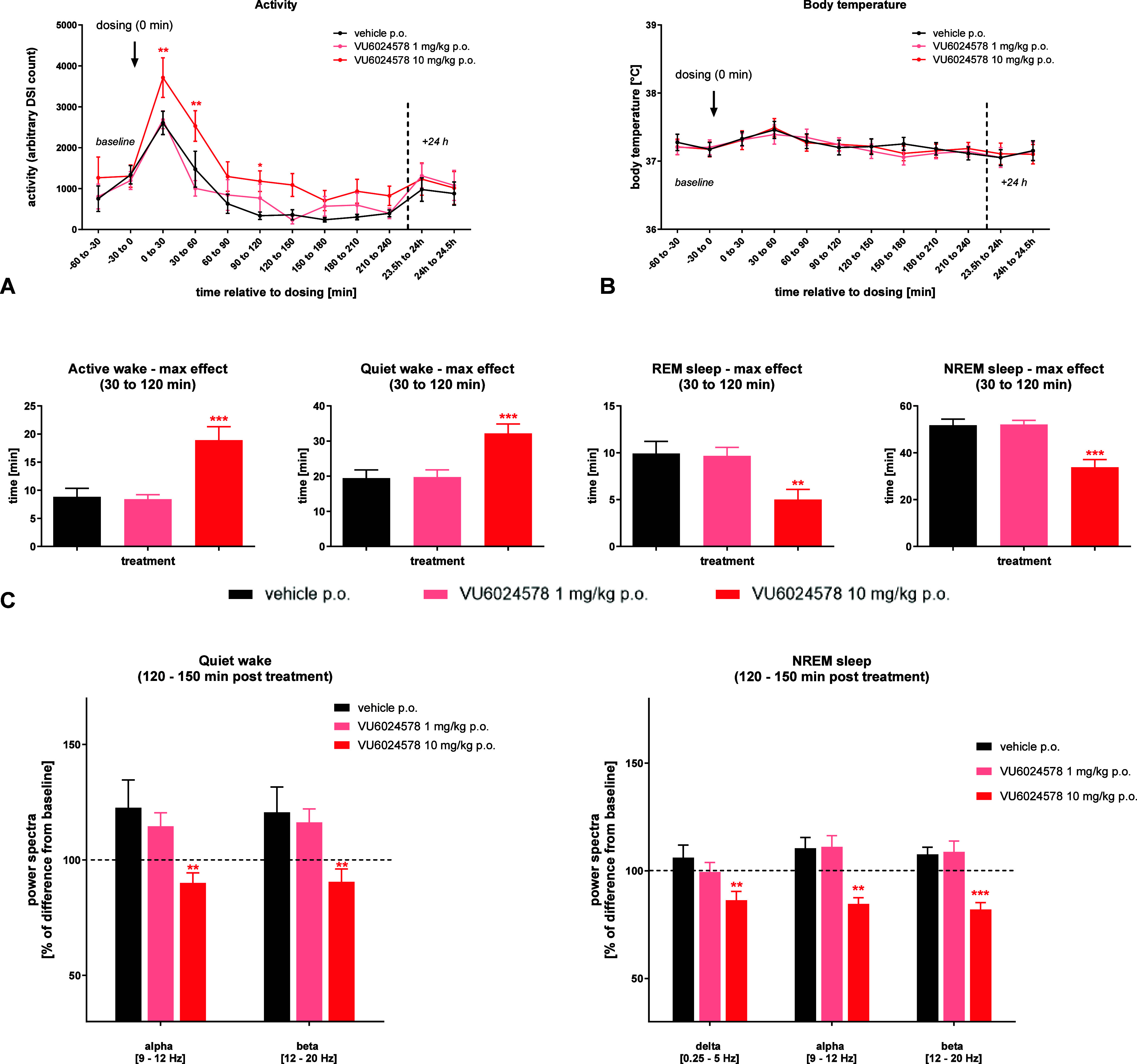
Effects of compound **19d** (1, 10 mg/kg, p.o., *n* = 14) on motor activity, body temperature, sleep-wake profile and EEG brain activity of rats: A) motors activity, B) body activity, C) vigilance states assessment and D), EEG power spectra. Data are expressed as mean ± SEM and were analyzed by one- and two-way ANOVA followed by Dunnett’s Multiple Comparisons tests (*p < 0.05, **p < 0.01, ***p < 0.001 versus vehicle).

We analyzed the effects of compound **19d** on motor activity, body temperature, sleep-wake profile/vigilance states (active wake, quiet wake, nonrapid eye movement (NREM) sleep and rapid eye movement (REM) sleep) and EEG brain activity/power spectra of conscious adult male. Treatment of rats with **19d** 10 mg/kg significantly increased motor activity showing a maximum effect within the first 120 min post treatment with no measurable changes in body temperature of rats (Figure 11**A,B**). Vigilance states analysis demonstrated a significant increase of time rats spent in active and quiet wake states by the higher dose of the test compound and a counterpart reduction of time spent in NREM and REM sleep. These effects were most pronounced between 30 and 120 min post treatment (Figure 11C). Regarding analysis of the EEG power spectra, the 10 mg/kg showed again a consistent reduction of delta, alpha and beta power affecting mostly quiet wake states and particularly NREM sleep with a long-lasting effect from 60 to 240 min post treatment (Figure 11**,D**). In conclusion, these data demonstrate that the mGluR_1_ PAM **19d** modulates central electrophysiological brain activity in rats inducing a specific sleep EEG fingerprint that can be used as translatable EEG biomarker.

**Figure 12:**
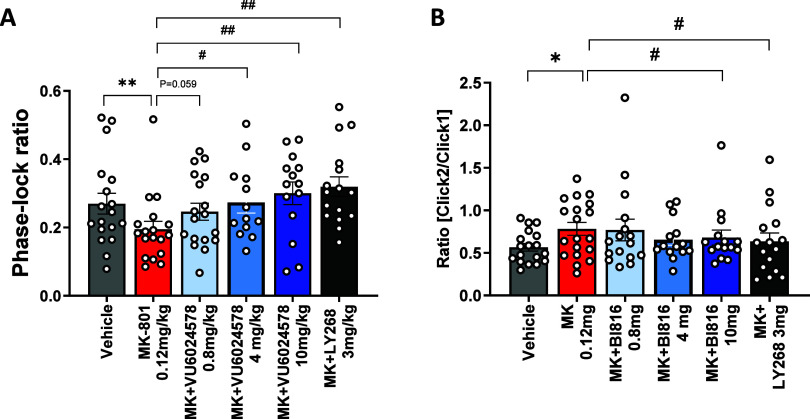
Modulation of auditory event related potentials in C57BL/6JRj mice following s.c. administration of different doses of compound 19d. The mGlu2/3 selective agonist LY379268 was used here as a positive control, applied also s.c. at 3 mg/kg dose. Effects of 0.8, 4, and 10 mg/kg compound 19d on ASSR 40 Hz Phase-lock coherence (A) and N1 gating (B) measured in prefrontal cortex were analyzed and compared to mice treated with MK-801 0.12 mg/kg or vehicle. Data are shown as mean ± SEM (*n* = 13–19 for each treatment group). *P < 0.05, **P < 0.01, ***P < 0.005 compared with vehicle; #*P* < 0.05, ##*P* < 0.01 compared with MK-801.

We have also employed here event-related potentials (ERPs) and auditory-evoked oscillations, which serve as critical translational biomarkers, to assess the impact of our investigational drug on sensory processing and cognitive functions, providing valuable data on its potential efficacy in treating disorders like schizophrenia. Detailed description of the methodology, setup specifications and recording procedure and analysis of the 40 Hz auditory steady-state response (ASSR) protocol can be found in Schuelert et al.^27^ The 40 Hz ASSR is one of the most established diagnostic EEG biomarker revealing a deficit of auditory steady-state synchronization in schizophrenia patients.^28−31^ Reduced entrainment of gamma oscillation reflects abnormalities in basic sensory registration and processing but is also associated with impaired higher cognitive functions.^32^ ASSR can also be reliably impaired preclinically by different NMDA receptor antagonists.^33,34^ The mGluR_1_ PAM **19d** significantly and dose-dependently reversed MK-801 induced deficits in 40 Hz ASSR intertrial coherence in the prefrontal cortex (PFC). The low dose of 0.8 mg/kg had no significant effect while the 4 mg/kg and the 10 mg/kg dose induced a complete reversal of the deficit (Figure 12A). Abnormalities associated with the filtering or “gating” of sensory information are among the most consistent findings in schizophrenia research.^35^ N100 gating deficits also correlated with negative-cognitive deficits dimensions of schizophrenia^36^ and can be modeled preclinically with NMDA receptor antagonists.^37^**19d** reversed N1 sensory gating deficit induced by MK-801 with the highest dose of 10 mg/kg (Figure 12B). Mean plasma exposures in satellite mice after subcutaneous administration of **19d** at 0.8, 4, and 10 mg/kg were 187, 1207, and 2490 nM, respectively. These values correspond to estimated compound CSF levels in the range of 0.3, 1.4 and 2.7x mGlu_1_ PAM EC_50_ of 27 nM. These data point to excellent potential for enablement of disease relevant clinical biomarkers, considering the high translatability of the above readouts to humans.

After achieving a more definitive PK/PD understanding of mGlu_1_ PAM efficacy in AHL and a translatable biomarker approach, we elected to continue profiling of **19d** in multispecies PK en route to a putative clinical candidate.

### Dog PK

Deeper profiling began with IV dog PK (0.5 mg/kg, n = 3 male beagles). Here, **19d** showed a very favorable profile with a CL_p_ of 5.4 mL/min/kg, a *t*_1/2_ of 3.6 h and a V_ss_ of 0.92 L/kg. C_0_ exposure was 2.05 μM total with 80 nM unbound (note the dog mGlu_1_ EC_50_ is 72 nM). Unexpectedly, adverse events (AEs) were noted in dog at this plasma exposure that was equivalent to the *in vitro* EC_50_. The veterinary staff noted GI-salivation and rigidity, but the AEs occurred 1–3 min postdose (*C*_max_) and subsided/returned to normal and the 24-h study was able to be completed. Was this a mechanism-based toxicity in dog? Could there be dog-specific pharmacology of mGlu_1_ activation? Were these AEs derived from the **19d** chemotype? Could a mGlu_1_/mGlu_5_ heterodimer be responsible for the observed AEs? Interestingly, much higher exposures elicited no AEs in rats with **19d**. In an attempt to address the chemotype question, we evaluated PAM **4** (VU6004909),^8^ an early generation rodent *in vivo* tool compound, for the first time in dog PK. Replication of the study with **19d**, PAM **4** produced no AEs in dog. Despite low clearance (CL_p_ = 14 mL/min/kg), a long half-life (16.8 h) and a large volume (V_ss_ = 12 L/kg), C_0_ exposure only achieved 1.1 μM total exposure and 36.4 nM unbound plasma exposure–far below the dog mGlu_1_ EC_50_ (400 nM). Therefore, more molecular pharmacology and PK studies as well as additional chemically distinct mGlu_1_ PAM chemotypes are required to understand the AEs elicited by an mGlu_1_ PAM in dogs, an otherwise very promising target for the treatment of schizophrenia.

## Conclusions

In summary, we disclosed progress toward a metabotropic glutamate receptor subtype 1 (mGlu_1_) positive allosteric modulator (PAM) clinical candidate, and the discovery of VU6024578/BI02982816. From a weak high-throughput screening hit (VU0538160, EC_50_ > 10 μM, 71% Gl*u*_max_), we were able to improve potency ∼185-fold to deliver a highly selective (inactive on mGlu_2–5,7,8_) and CNS penetrant (rat K_p_ = 0.99, K_p,uu_ = 0.82; MDCK-MDR1 ER = 1.7, P_app_ = 73 × 10–6 cm/s) mGlu_1_ PAM (VU6024578/BI02982816, EC_50_ = 54 nM, 83% Gl*u*_max_). VU6024578/BI02982816 was found to active in both amphetamine-induced hyperlocomotion (minimum effective dose (MED) = 3 mg/kg, p.o.) and MK-801 induced disruptions of novel object recognition (minimum effective dose = 10 mg/kg p.o.), thus providing robust efficacy in preclinical models of psychosis and cognition, as well as a robust PK/PD relationship driven by C_trough_ versus *C*_max_. Progress was also made in terms of clinical biomarkers employing event-related electroencephalography readouts. However, unanticipated AEs in dog prevented further consideration as a clinical candidate, and resources shifted to orthogonal chemotypes and deeper mechanistic exploration of mGlu_1_. Nevertheless, VU6024578/BI02982816 can serve as a best-in-class *in vivo* rodent tool compound to study selective mGlu_1_ activation.

## Experimental Section

### General Chemistry

All reactions were carried out employing standard chemical techniques under an inert atmosphere. Solvents used for extraction, washing, and chromatography were HPLC grade.All reagents were purchased from commercial sources and were used without further purification. All microwave reactions were carried out in sealed tubes in a Biotage Initiator microwave synthesis reactor. Temperature control was automated via IR sensor and all indicated temperatures correspond to the maximal temperature reached during each experiment. Analytical HPLC was performed on an Agilent 1200LCMS with UV detection at 215 and 254 nm along with ELSD detection and electrospray ionization, with all final compounds showing >95% purity and a parent mass ion consistent with the desired structure. Low resolution mass spectra were obtained on an Agilent 6120 or 6150 with ESI source. All NMR spectra were recorded on a 400 MHz Brüker AV-400 instrument. 1H chemical shifts are reported as δ values in ppm relative to the residual solvent peak (CDCl3 = 7.26). Data are reported as follows: chemical shift, multiplicity (br. = broad, s = singlet, d = doublet, t = triplet, q = quartet, dd = doublet of doublets, m = multiplet), coupling constant(Hz), and integration. 13C chemical shifts are reported as δ values in ppm relative to the residual solvent peak (CDCl3 = 77.16). High resolution mass spectra were obtained on an Agilent 6540 UHD Q-TOF with ESI source. Automated flash column chromatography was performed on a Teledyne ISCO Combiflash Rf system. For compounds that were purified on a Gilson preparative reversed-phase HPLC, the system comprised of a 333 aqueous pump with solvent-selection valve, 334 organic pump, GX-271 or GX-281 liquid hander, two column switching valves, and a 155 UV detector. UV wavelength for fraction collection was user-defined, with absorbance at 254 nm always monitored. Method: Phenomenex Axia-packed Luna C18, 30 × 50 mm, 5 μm column. Mobile phase: CH3CN inH2O (0.1% TFA). Gradient conditions: 0.75 min equilibration, followed by user-defined gradient (starting organic percentage, ending organic percentage, duration), hold at 95% CH3CN in H2O (0.1%TFA) for 1 min, 50 mL/min, 23 °C. Melting points were recorded on an OptiMelt automated melting point system by Stanford Research Systems. cLogP, MW, and TPSA were calculated using PerkinElmer ChemDraw professional version 20.1.0.110. All final compounds were purified to >95% as determined by analytical LCMS (214 nm, 254 nm, and ELSD), ^1^H and/or ^13^C NMR, and high-resolution MS.

#### General Procedure 1

##### Synthesis of Analogs **6** and **9a**-**9e**

To a solution of the respective benzoic acid (1.1 equiv) and *N*,*N*-diisopropylethylamine (3.5 equiv) in DMF (0.13 M) was added HATU (1.2 equiv), and the resulting reaction was stirred at rt for 5 min before the addition of 1-(aminomethyl)-3-azaindolizine hydrochloride (1 equiv). The reaction was then stirred at rt for 1 h.

#### Purification Method A

The crude reaction mixture was purified via reverse phase HPLC (Gilson 30 × 100 mm basic column: H_2_O with 0.05% NH_4_OH/MeCN: 10–80% MeCN gradient) to afford the desired product.

#### Purification Method B

The crude reaction mixture was diluted with EtOAc and sequentially washed with water and brine. The combined organic material was passed through a phase separator, concentrated, and purified via flash chromatography (Teledyne ISCO flash purification system; silica gel column, hexanes:EtOAc; 5–80% EtOAc gradient) to afford the desired product.

##### Synthesis of 5-Chloro-2-methoxy-*N*-(pyrazolo[1,5-*a*]pyridin-3-ylmethyl)benzamide (**6**)

This compound was synthesized according to general procedure 1 and purified via purification method A. Light tan solid (15 mg, 73% yield). ^1^H NMR (400 MHz, DMSO-*d*_6_): 8.67 (t, *J* = 5.8 Hz, 1H), 8.62 (dt, *J* = 7.0, 1.1 Hz, 1H), 7.96 (s, 1H), 7.82 (dt, *J* = 8.9, 1.3 Hz, 1H), 7.65 (d, *J* = 2.8 Hz, 1H), 7.49 (dd, *J* = 8.9, 2.8 Hz, 1H), 7.24–7.20 (m, 1H), 7.14 (d, *J* = 8.9 Hz, 1H), 6.86 (td, *J* = 6.8, 1.4 Hz, 1H), 4.62 (d, *J* = 5.8 Hz, 2H), 3.83 (s, 3H). ^13^C NMR (100 MHz, DMSO-*d*_6_): 163.7, 155.7, 141.4, 137.7, 131.5, 129.5, 128.7, 125.1, 124.3, 123.0, 117.2, 114.1, 112.0, 108.6, 56.3, 32.9. HRMS: (Q-TOF, ES+): calc’d for C_16_H_15_ClN_3_O_2_ (M+H)^+^, 316.0847; found, 316.0850.

##### Synthesis of 2,5-Dimethoxy-*N*-(pyrazolo[1,5-*a*]pyridin-3-ylmethyl)benzamide (**9a**)

This compound was synthesized according to general procedure 1 and purified via purification method A. Colorless oil (19 mg, 93% yield). ^1^H NMR (400 MHz, DMSO-*d*_6_): 8.65–8.61 (m, 2H), 7.96 (s, 1H), 7.83 (dt, *J* = 9.0, 1.2 Hz, 1H), 7.30 (dd, *J* = 3.0, 0.6 Hz, 1H), 7.23–7.19 (m, 1H), 7.07–7.00 (m, 2H), 6.86 (td, *J* = 6.8, 1.4 Hz, 1H), 4.62 (d, *J* = 5.8 Hz, 2H), 3.78 (s, 3H), 3.72 (s, 3H). ^13^C NMR (100 MHz, DMSO-*d*_6_): 164.5, 152.9, 151.1, 141.4, 137.7, 128.7, 123.5, 123.0, 117.5, 117.2, 115.0, 113.5, 112.0, 108.8, 56.4, 55.5, 32.8.HRMS: (Q-TOF, ES+): calc’d for C_17_H_18_N_3_O_3_ (M+H)^+^, 312.1343; found, 312.1347.

##### Synthesis of 2-Methoxy-5-methyl-*N*-(pyrazolo[1,5-*a*]pyridin-3-ylmethyl)benzamide (**9b**)

This compound was synthesized according to general procedure 1 and purified via purification method A. Colorless oil (16 mg, 83% yield). ^1^H NMR (400 MHz, DMSO-*d*_6_): 8.62 (dt, *J* = 7.0, 1.1 Hz, 1H), 8.56 (t, *J* = 5.8 Hz, 1H), 7.95 (s, 1H), 7.83 (dt, *J* = 8.9, 1.2 Hz, 1H), 7.55 (d, *J* = 2.3 Hz, 1H), 7.26–7.19 (m, 2H), 6.99 (d, *J* = 8.4 Hz, 1H), 6.85 (td, *J* = 6.8, 1.4 Hz, 1H), 4.61 (d, *J* = 5.8 Hz, 2H), 3.79 (s, 3H), 2.25 (s, 3H). ^13^C NMR (100 MHz, DMSO-*d*_6_): 165.0, 154.9, 141.4, 137.7, 132.5, 130.7, 129.2, 128.7, 123.0, 122.7, 117.3, 112.0, 109.0, 55.9, 32.7, 19.9. HRMS: (Q-TOF, ES+): calc’d for C_17_H_18_N_3_O_2_ (M+H)^+^, 296.1394; found, 296.1397.

##### Synthesis of 5-Chloro-2-methyl-*N*-(pyrazolo[1,5-*a*]pyridin-3-ylmethyl)benzamide (**9c**)

This compound was synthesized according to general procedure 1 and purified via purification method A. Off-white solid (18 mg, 92% yield). ^1^H NMR (400 MHz, DMSO-*d*_6_): 8.82 (t, *J* = 5.7 Hz, 1H), 8.64 (dt, *J* = 7.0, 1.1 Hz, 1H), 7.97 (s, 1H), 7.79 (dt, *J* = 9.0, 1.2 Hz, 1H), 7.36 (dd, *J* = 8.2, 2.3 Hz, 1H), 7.31 (d, *J* = 2.3 Hz, 1H), 7.26–7.20 (m, 2H), 6.87 (td, *J* = 6.8, 1.4 Hz, 1H), 4.57 (d, *J* = 5.7 Hz, 2H), 2.25 (s, 3H). ^13^C NMR (100 MHz, DMSO-*d*_6_): 167.4, 141.4, 138.5, 137.8, 134.3, 132.3, 129.9, 129.0, 128.8, 126.7, 123.1, 117.1, 112.0, 108.5, 54.9, 32.5, 18.7. HRMS: (Q-TOF, ES+): calc’d for C_16_H_15_ClN_3_O (M+H)^+^, 300.0898; found, 300.0902.

##### Synthesis of 2,5-Dichloro-*N*-(pyrazolo[1,5-*a*]pyridin-3-ylmethyl)benzamide (**9d**)

This compound was synthesized according to general procedure 1 and purified via purification method A. Off-white solid (17 mg, 81% yield). ^1^H NMR (400 MHz, DMSO-*d*_6_): 8.97 (t, *J* = 5.6 Hz, 1H), 8.64 (dt, *J* = 7.0, 1.1 Hz, 1H), 7.99 (s, 1H), 7.78 (dt, *J* = 9.0, 1.3 Hz, 1H), 7.53–7.48 (m, 3H), 7.25–7.21 (m, 1H), 6.88 (td, *J* = 6.8, 1.4 Hz, 1H), 4.59 (d, *J* = 5.6 Hz, 2H). ^13^C NMR (100 MHz, DMSO-*d*_6_): 164.8, 141.4, 138.3, 137.8, 131.6, 131.4, 130.5, 128.8, 128.5, 123.1, 117.1, 112.1, 108.0, 32.7. HRMS: (Q-TOF, ES+): calc’d for C_15_H_12_Cl_2_N_3_O (M+H)^+^, 320.0352; found, 320.0354.

##### Synthesis of 5-Bromo-2-chloro-*N*-(pyrazolo[1,5-*a*]pyridin-3-ylmethyl)benzamide (**9e**)

This compound was synthesized according to general procedure 1 and purified via purification method B. Beige solid (313 mg, 79% yield). ^1^H NMR (400 MHz, DMSO-*d*_6_): 8.97 (t, *J* = 5.7 Hz, 1H), 8.64 (d, *J* = 7.1 Hz, 1H), 7.99 (s, 1H), 7.78 (d, *J* = 8.9 Hz, 1H), 7.64–7.59 (m, 2H), 7.44 (d, *J* = 8.4 Hz, 1H), 7.25–7.21 (m, 1H), 6.88 (td, *J* = 6.8, 1.4 Hz, 1H), 4.59 (d, *J* = 5.6 Hz, 2H). ^13^C NMR (100 MHz, DMSO-*d*_6_): 164.6, 141.4, 138.6, 137.8, 133.4, 131.6, 131.3, 129.3, 128.8, 123.1, 119.8, 117.1, 112.1, 108.0, 32.7. HRMS: calc’d for C_15_H_12_BrClN_3_O (M+H)^+^, 363.9847; found, 363.9847

##### General Procedure 2: Synthesis of Analogs **9f**–**9k**

A vial was charged with 5-bromo-2-chloro-*N*-(pyrazolo[1,5-*a*]pyridin-3-ylmethyl)benzamide (**9e**) (1.0 equiv), the respective boronic acid or boronic acid pinacol ester (1.2 equiv), Pd(dppf)Cl_2_ (0.1 equiv), Cs_2_CO_3_ (3 equiv), and a 10:1 mixture of 1,4-dioxane:water (0.15 M). The resulting reaction mixture was sparged with nitrogen (g) for 1 min before the reaction vial was sealed and heated to 100 °C for 2 h. Upon cooling to rt, the crude reaction mixture was diluted with EtOAc, passed through a syringe PTFE filter, concentrated, and purified via flash chromatography (Teledyne ISCO flash purification system; silica gel column, DCM:MeOH; 0–10% MeOH gradient) to afford the desired product.

##### Synthesis of 4-Chloro-*N*-(pyrazolo[1,5-*a*]pyridin-3-ylmethyl)-[1,1′-biphenyl]-3-carboxamide (**9f**)

This compound was synthesized according to general procedure 2. Off-white solid (21 mg, 70% yield). ^1^H NMR (400 MHz, DMSO-*d*_6_): 8.97 (t, *J* = 5.7 Hz, 1H), 8.64 (d, *J* = 7.0 Hz, 1H), 8.00 (s, 1H), 7.82 (d, *J* = 9.0 Hz, 1H), 7.73–7.65 (m, 4H), 7.56 (d, *J* = 8.4 Hz, 1H), 7.49–7.45 (m, 2H), 7.41–7.37 (m, 1H), 7.25–7.21 (m, 1H), 6.88 (td, *J* = 6.8, 1.4 Hz, 1H), 4.62 (d, *J* = 5.7 Hz, 2H). ^13^C NMR (100 MHz, DMSO-*d*_6_): 166.0, 141.4, 138.9, 138.3, 137.8, 137.1, 130.3, 129.3, 129.1, 128.8, 128.8, 128.1, 127.0, 126.7, 123.1, 117.2, 112.1, 108.3, 32.7. HRMS: (Q-TOF, ES+): calc’d for C_21_H_17_ClN_3_O (M+H)^+^, 362.1055; found, 362.1060

##### Synthesis of 2-Chloro-*N*-(pyrazolo[1,5-*a*]pyridin-3-ylmethyl)-5-(thiophen-2-yl)benzamide (**9g**)

This compound was synthesized according to general procedure 2. Pale yellow solid (10 mg, 33% yield). ^1^H NMR (400 MHz, DMSO-*d*_6_): 8.98 (t, *J* = 5.7 Hz, 1H), 8.64 (dt, *J* = 7.0, 1.1 Hz, 1H), 8.00 (s, 1H), 7.81 (dt, *J* = 9.0, 1.3 Hz, 1H), 7.69 (dd, *J* = 8.4, 2.4 Hz, 1H), 7.64 (d, *J* = 2.3 Hz, 1H), 7.61–7.59 (m, 2H), 7.50 (d, *J* = 8.4 Hz, 1H), 7.26–7.22 (m, 1H), 7.15 (dd, *J* = 5.0, 3.7 Hz, 1H), 6.88 (td, *J* = 6.8, 1.4 Hz, 1H), 4.62 (d, *J* = 5.7 Hz, 2H). ^13^C NMR (100 MHz, DMSO-*d*_6_): 165.8, 141.4, 141.2, 137.8, 137.4, 132.7, 130.4, 128.8, 127.3, 126.7, 125.4, 124.9, 123.1, 117.2, 112.1, 108.2, 32.7. HRMS: (Q-TOF, ES+): calc’d for C_19_H_15_ClN_3_OS (M+H)^+^, 368.0619; found, 368.0623

##### Synthesis of 2-Chloro-*N*-(pyrazolo[1,5-*a*]pyridin-3-ylmethyl)-5-(thiophen-3-yl)benzamide (**9h**)

This compound was synthesized according to general procedure 2. Off-white solid (15 mg, 50% yield). ^1^H NMR (400 MHz, DMSO-*d*_6_): 8.95 (t, *J* = 5.7 Hz, 1H), 8.64 (d, *J* = 7.0 Hz, 1H), 8.00 (s, 1H), 7.98 (dd, *J* = 2.9, 1.4 Hz, 1H), 7.82 (d, *J* = 9.0 Hz, 1H), 7.78–7.74 (m, 2H), 7.65 (dd, *J* = 5.0, 2.9 Hz, 1H), 7.59 (dd, *J* = 5.1, 1.4 Hz, 1H), 7.49 (d, *J* = 8.2 Hz, 1H), 7.25–7.21 (m, 1H), 6.88 (td, *J* = 6.9, 1.4 Hz, 1H), 4.62 (d, *J* = 5.7 Hz, 2H). ^13^C NMR (100 MHz, DMSO-*d*_6_): 166.1, 141.4, 139.6, 137.8, 137.2, 134.0, 130.1, 128.8, 128.4, 128.1, 127.5, 126.2, 126.1, 123.1, 122.2, 117.2, 112.1, 108.2, 32.7. HRMS: (Q-TOF, ES+): calc’d for C_19_H_15_ClN_3_OS (M+H)^+^, 368.0619; found, 368.0623

##### Synthesis of 2-Chloro-*N*-(pyrazolo[1,5-*a*]pyridin-3-ylmethyl)-5-(thiazol-2-yl)benzamide (**9i**)

A vial was charged with 5-bromo-2-chloro-*N*-(pyrazolo[1,5-*a*]pyridin-3-ylmethyl)benzamide (**9e**) (50 mg, 0.14 mmol), bis(pinacolato)diboron (52 mg, 0.21 mmol), potassium acetate (40 mg, 0.41 mmol), Pd(dppf)Cl_2_ (10 mg, 0.014 mmol), and 1,4-dioxane (1 mL). The resulting reaction was sparged with nitrogen for ∼1 min before the reaction vial was sealed and heated to 100 °C for 3 h (LCMS indicated complete conversion to intermediate boronic pinacol ester). Upon cooling to rt, the crude reaction mixture was diluted with EtOAc, filtered, and concentrated. The resulting black residue was dissolved in 1,4-dioxane (0.75 mL) and water (75 μL). Cesium carbonate (134 mg, 0.41 mmol), Pd(dppf)Cl_2_ (10 mg, 0.014 mmol), and 2-bromothiazole (15 μL, 0.16 mmol) were then added. The crude reaction mixture was sparged with nitrogen for ∼1 min before the vial was sealed and heated to 100 °C for 2 h. Upon cooling to rt, the crude reaction mixture was diluted with EtOAc, filtered, and purified via flash chromatography (Teledyne ISCO flash purification system; silica gel column, DCM:MeOH; 0–10% MeOH gradient) to afford the desired product as an off-white powder (12 mg, 24% yield). ^1^H NMR (400 MHz, DMSO-*d*_6_): 9.04 (t, *J* = 5.7 Hz, 1H), 8.65 (dt, *J* = 7.0, 1.1 Hz, 1H), 8.00 (s, 1H), 7.98 (dd, *J* = 8.4, 2.3 Hz, 1H), 7.95 (d, *J* = 3.2 Hz, 1H), 7.91 (d, *J* = 2.2 Hz, 1H), 7.85 (d, *J* = 3.2 Hz, 1H), 7.82 (dt, *J* = 9.0, 1.3 Hz, 1H), 7.61 (d, *J* = 8.4 Hz, 1H), 7.26–7.22 (m, 1H), 6.89 (td, *J* = 6.8, 1.4 Hz, 1H), 4.63 (d, *J* = 5.7 Hz, 2H). ^13^C NMR (100 MHz, DMSO-*d*_6_): 165.4, 165.1, 144.1, 141.4, 137.8, 137.4, 131.8, 131.5, 130.7, 128.8, 128.1, 126.1, 123.1, 121.5, 117.2, 112.1, 108.2, 32.7. HRMS: (Q-TOF, ES+): calc’d for C_18_H_14_ClN_4_OS (M+H)^+^, 369.0571; found, 369.0574

##### Synthesis of 2-Chloro-5-(1-methyl-1*H*-pyrazol-5-yl)-*N*-(pyrazolo[1,5-*a*]pyridin-3-ylmethyl)benzamide (**9j**)

This compound was synthesized according to general procedure 2. Off-white powder (20 mg, 66% yield). ^1^H NMR (400 MHz, DMSO-*d*_6_): 8.97 (t, *J* = 5.6 Hz, 1H), 8.64 (d, *J* = 7.0 Hz, 1H), 7.99 (s, 1H), 7.80 (dt, *J* = 8.9, 1.3 Hz, 1H), 7.59 (d, *J* = 1.3 Hz, 2H), 7.53 (t, *J* = 1.3 Hz, 1H), 7.47 (d, *J* = 1.9 Hz, 1H), 7.25–7.21 (m, 1H), 6.87 (td, *J* = 6.8, 1.4 Hz, 1H), 6.46 (d, *J* = 1.9 Hz, 1H), 4.62 (d, *J* = 5.6 Hz, 2H), 3.84 (s, 3H). ^13^C NMR (100 MHz, DMSO-*d*_6_): 165.6, 141.4, 140.9, 138.1, 137.8, 137.1, 130.4, 130.1, 130.0, 129.0, 128.8, 128.6, 123.1, 117.2, 112.1, 108.1, 106.3, 37.6, 32.7. HRMS: (Q-TOF, ES+): calc’d for C_19_H_17_ClN_5_O (M+H)^+^, 366.1116; found, 366.1121

##### Synthesis of 2-Chloro-5-(furan-2-yl)-*N*-(pyrazolo[1,5-*a*]pyridin-3-ylmethyl)benzamide (**9k**)

This compound was synthesized according to general procedure 2. Orange glass (9 mg, 31% yield). ^1^H NMR (400 MHz, DMSO-*d*_6_): 8.97 (t, *J* = 5.7 Hz, 1H), 8.65 (dt, *J* = 7.0, 1.1 Hz, 1H), 7.99 (s, 1H), 7.81 (dt, *J* = 8.9, 1.3 Hz, 1H), 7.77 (dd, *J* = 1.8, 0.7 Hz, 1H), 7.73 (dd, *J* = 8.4, 2.2 Hz, 1H), 7.69 (d, *J* = 2.2 Hz, 1H), 7.52 (d, *J* = 8.4 Hz, 1H), 7.26–7.22 (m, 1H), 7.08 (dd, *J* = 3.4, 0.8 Hz, 1H), 6.88 (td, *J* = 6.8, 1.4 Hz, 1H), 6.61 (dd, *J* = 3.4, 1.8 Hz, 1H), 4.61 (d, *J* = 5.7 Hz, 2H). ^13^C NMR (100 MHz, DMSO-*d*_6_): 165.8, 151.3, 143.6, 141.4, 137.8, 137.3, 130.2, 129.1, 128.8, 128.5, 125.3, 123.4, 123.1, 117.2, 112.4, 112.1, 108.2, 107.3, 32.6. HRMS: (Q-TOF, ES+): calc’d for C_19_H_15_ClN_3_O_2_ (M+H)^+^, 352.0847; found, 352.0852

##### Synthesis of 2-Chloro-5-(furan-2-yl)benzoic acid (**13**)

A RBF was charged with furan-2-boronic acid pinacol ester (6.18 g, 31.9 mmol), 5-bromo-2-chlorobenzoic acid (5.00 g, 21.2 mmol), tetrakis(triphenylphosphine)palladium(0) (736 mg, 0.64 mmol) and sodium carbonate (6.88 g, 63.7 mmol) in 1,4-dioxane (60 mL) and water (15 mL). The reaction was sparged with nitrogen for ∼5 min before the flask was sealed and heated to 80 °C for 1 h. The solution was cooled, diluted with EtOAc, and then extracted with 1 M NaOH x 2. The combined aqueous material was washed with EtOAc and then acidified with conc. HCl. The resulting solids were collected via vacuum filtration (washing with H_2_O) and then dried *in vacuo* to afford 2-chloro-5-(furan-2-yl)benzoic acid (3.78 g,17.0 mmol, 80% yield) as a white solid. ^1^H NMR (400 MHz, DMSO-*d*_6_): 8.06 (d, *J* = 2.3 Hz, 1H), 7.83 (dd, *J* = 8.5, 2.3 Hz, 1H), 7.80 (dd, *J* = 1.8, 0.8 Hz, 1H), 7.59 (d, *J* = 8.4 Hz, 1H), 7.11 (dd, *J* = 3.4, 0.8 Hz, 1H), 6.63 (dd, *J* = 3.4, 1.8 Hz, 1H). ^13^C NMR (100 MHz, DMSO-*d*_6_): 166.4, 151.0, 143.8, 132.1, 131.3, 130.0, 129.2, 127.0, 125.2, 112.4, 107.5. HRMS (Q-TOF, ES+): calc’d for C_11_H_8_ClO_3_ (M+H)^+^, 223.0156; found, 223.0156.

##### General Procedure 3: Synthesis of Analogs **15a**–**15f**

To a solution of 2-chloro-5-(furan-2-yl)benzoic acid (**13**) (1.0 equiv) in DMF (0.05 M) was sequentially added *N*,*N*-diisopropylethylamine (3 eq if free base of amine is going to be used or 4 eq if the amine HCl salt is going to be used) and HATU (1.1 equiv). The resulting reaction was stirred at rt for 5 min before the addition of either the respective amine or the respective amine HCl salt (1.1 equiv). The reaction mixture was then stirred at rt for 1 h before being purified via reverse phase HPLC (Gilson 30 × 100 mm basic column: H_2_O with 0.05% NH_4_OH/MeCN: 5–95% MeCN gradient) to afford the desired products.

##### Synthesis of 2-Chloro-5-(furan-2-yl)-*N*-((3-phenylisoxazol-5-yl)methyl)benzamide (**15a**)

This compound was synthesized according to general procedure 3. White solid (12.3 mg, 60% yield). ^1^H NMR (400 MHz, DMSO-*d*_6_): 9.29 (t, *J* = 5.8 Hz, 1H), 7.91–7.85 (m, 2H), 7.82–7.80 (m, 2H), 7.78 (dd, *J* = 8.4, 2.2 Hz, 1H), 7.57 (d, *J* = 8.4 Hz, 1H), 7.56–7.47 (m, 3H), 7.12 (d, *J* = 3.4 Hz, 1H), 6.98 (s, 1H), 6.63 (dd, *J* = 3.4, 1.8 Hz, 1H), 4.67 (d, *J* = 6.0 Hz, 2H). ^13^C NMR (100 MHz, DMSO-*d*_6_): 170.5, 166.2, 161.9, 151.3, 143.7, 136.6, 130.3, 130.2, 129.2, 129.1, 128.6, 128.5, 126.6, 125.6, 123.6, 112.4, 107.4, 100.2, 35.1. HRMS (Q-TOF, ES+): calc’d for C_21_H_16_ClN_2_O_3_ (M+H)^+^, 379.0844; found, 379.0851.

##### Synthesis of 2-Chloro-5-(furan-2-yl)-*N*-((5-phenyl-1,2,4-oxadiazol-3-yl)methyl)benzamide (**15b**)

This compound was synthesized according to general procedure 3. White solid (10.6 mg, 52% yield). ^1^H NMR (400 MHz, DMSO-*d*_6_): 9.29 (t, *J* = 5.8 Hz, 1H), 8.17–8.10 (m, 2H), 7.82–7.76 (m, 3H), 7.76–7.70 (m, 1H), 7.69–7.61 (m, 2H), 7.58–7.55 (m, 1H), 7.09 (d, *J* = 3.4, 1H), 6.63 (dd, *J* = 3.4, 1.8 Hz, 1H), 4.67 (d, *J* = 5.8 Hz, 2H). ^13^C NMR (100 MHz, DMSO-*d*_6_): 175.2, 168.7, 166.3, 151.3, 143.7, 136.6, 133.3, 130.4, 129.6, 129.1, 128.6, 127.8, 125.6, 123.5, 123.3, 112.4, 107.3, 35.0. HRMS (Q-TOF, ES+): calc’d for C_20_H_15_ClN_3_O_3_ (M+H)^+^, 380.0796; found, 380.0801.

##### Synthesis of 2-Chloro-*N*-((5-cyclopropyl-1,2,4-oxadiazol-3-yl)methyl)-5-(furan-2-yl)benzamide (**15c**)

This compound was synthesized according to general procedure 3. Beige glass (13 mg, 70% yield). ^1^H NMR (400 MHz, DMSO-*d*_6_): 9.16 (t, *J* = 5.9 Hz, 1H), 7.80 (dd, *J* = 1.8, 0.7 Hz, 1H), 7.77 (dd, *J* = 8.4, 2.2 Hz, 1H), 7.74 (d, *J* = 2.1 Hz, 1H), 7.55 (d, *J* = 8.4 Hz, 1H), 7.08 (dd, *J* = 3.4, 0.8 Hz, 1H), 6.63 (dd, *J* = 3.4, 1.8 Hz, 1H), 4.50 (d, *J* = 5.9 Hz, 2H), 2.34 (m, 1H), 1.28–1.23 (m, 2H), 1.12–1.08 (m, 2H). ^13^C NMR (100 MHz, DMSO-*d*_6_): 181.7, 167.8, 166.2, 151.3, 143.7, 136.6, 130.4, 129.1, 128.6, 125.6, 123.5, 112.4, 107.3, 34.9, 10.0, 7.1. HRMS (Q-TOF, ES+): calc’d for C_17_H_15_ClN_3_O_3_ (M+H)^+^, 344.0796; found, 344.0803.

##### Synthesis of 2-Chloro-*N*-((3-cyclopropyl-1,2,4-oxadiazol-5-yl)methyl)-5-(furan-2-yl)benzamide (**15d**)

This compound was synthesized according to general procedure 3. White solid (12.8 mg, 69% yield). ^1^H NMR (400 MHz, DMSO-*d*_6_): 9.34 (t, *J* = 5.8 Hz, 1H), 7.96–7.67 (m, 3H), 7.57 (d, *J* = 8.4 Hz, 1H), 7.09 (d, *J* = 3.3 Hz, 1H), 6.64 (dd, *J* = 3.4, 1.8 Hz, 1H), 4.68 (d, *J* = 5.8 Hz, 2H), 2.14 (tt, *J* = 8.3, 4.8 Hz, 1H), 1.11–1.06 (m, 2H), 0.92–0.88 (m, 2H). ^13^C NMR (100 MHz, DMSO-*d*_6_): 176.4, 171.8, 166.5, 151.2, 143.7, 136.2, 130.4, 129.2, 128.6, 125.8, 123.5, 112.4, 107.4, 35.5, 7.4, 6.3. HRMS (Q-TOF, ES+): calc’d for C_17_H_15_ClN_3_O_3_ (M+H)^+^, 344.0796; found, 344.0801.

##### Synthesis of 2-Chloro-5-(furan-2-yl)-*N*-((2-(thiophen-2-yl)thiazol-5-yl)methyl)benzamide (**15e**)

This compound was synthesized according to general procedure 3 using **Amine 1a** as the amine HCl salt (the synthesis for **Amine 1a** is described later in this document). White solid (16 mg, 74% yield). ^1^H NMR (400 MHz, DMSO-*d*_6_): 9.27 (t, *J* = 4.0 Hz, 1H), 7.79 (dd, *J* = 1.8, 0.7 Hz, 1H), 7.76 (dd, *J* = 8.4, 2.2 Hz, 1H), 7.74–7.71 (m, 2H), 7.70 (dd, *J* = 5.1, 1.1 Hz, 1H), 7.64 (dd, *J* = 3.7, 1.1 Hz, 1H), 7.55 (d, *J* = 8.4 Hz, 1H), 7.16 (dd, *J* = 5.1, 3.7 Hz, 1H), 7.11 (dd, *J* = 3.4, 0.8 Hz, 1H), 6.62 (dd, *J* = 3.4, 1.8 Hz, 1H), 4.66 (d, *J* = 5.0 Hz, 2H). ^13^C NMR (100 MHz, DMSO-*d*_6_): 166.1, 160.8, 151.2, 143.7, 141.5, 136.8, 136.2, 130.4, 129.2, 128.61, 128.56, 128.4, 127.1, 125.5, 123.4, 112.4, 107.4, 35.3. HRMS (Q-TOF, ES+): calc’d for C_19_H_14_ClN_2_O_2_S_2_ (M+H)^+^, 401.0180; found, 401.0181.

##### Synthesis of 2-Chloro-5-(furan-2-yl)-*N*-((1-methyl-3-(trifluoromethyl)-1*H*-pyrazol-5-yl)methyl)benzamide (**15f**)

This compound was synthesized according to general procedure 3. Tan solid (11.4 mg, 55% yield). ^1^H NMR (400 MHz, DMSO-*d*_6_): 9.12 (t, *J* = 5.6 Hz, 1H), 7.80 (dd, *J* = 1.8, 0.7 Hz, 1H), 7.78–7.75 (m, 2H), 7.58–7.53 (m, 1H), 7.11 (dd, *J* = 3.4, 0.8 Hz, 1H), 6.67 (s, 1H), 6.63 (dd, *J* = 3.4, 1.8 Hz, 1H), 4.58 (d, *J* = 5.6 Hz, 2H), 3.93 (s, 3H). ^13^C NMR (100 MHz, DMSO-*d*_6_): 166.0, 151.2, 143.7, 142.0, 138.9 (q, *J*_*CF*_ = 37 Hz), 136.8, 130.3, 129.2, 128.4, 125.5, 123.5, 121.5 (q, *J*_*CF*_ = 266 Hz), 112.3, 107.4, 104.1, 37.2, 33.8. HRMS (Q-TOF, ES+): calc’d for C_17_H_14_ClF_3_N_3_O_2_ (M+H)^+^, 384.0721; found, 384.0726.

##### Synthesis of *tert*-Butyl ((2-(Thiophen-2-yl)thiazol-5-yl)methyl)carbamate (**Amine 1**)

A vial was charged with 5-(Boc-aminomethyl)-2-bromothiazole (400 mg, 1.4 mmol), 2-thienylboronic acid (209 mg, 1.6 mmol), potassium carbonate (383 mg, 2.7 mmol), Pd(dppf)Cl_2_ (100 mg, 0.14 mmol), 1,4-dioxane (8 mL), and water (0.8 mL). The reaction mixture was sparged with nitrogen for ∼5 min before the reaction vial was sealed and heated to 90 °C for 16 h. Upon cooling to rt, the reaction mixture was diluted with EtOAc, filtered through a pad of Celite, concentrated, and purified via flash chromatography (Teledyne ISCO flash purification system; silica gel column; hexane: EtOAc; 0–50% EtOAc gradient) to afford the desired product *tert*-butyl ((2-(thiophen-2-yl)thiazol-5-yl)methyl)carbamate (**Amine 1**) (225 mg, 0.76 mmol, 56% yield) as an off-white solid. ^1^H NMR (400 MHz, DMSO-*d*_6_): 7.68 (dd, *J* = 5.0, 1.2 Hz, 1H), 7.60 (dd, *J* = 3.7, 1.1 Hz, 1H), 7.59–7.50 (m, 2H), 7.14 (dd, *J* = 5.1, 3.7 Hz, 1H), 4.31 (d, *J* = 6.0 Hz, 2H), 1.40 (s, 9H). ^13^C NMR (100 MHz, DMSO-*d*_6_): 160.4, 155.6, 140.8, 137.7, 136.8, 128.5, 128.4, 126.9, 78.3, 36.2, 28.2. HRMS (Q-TOF, ES+): calc’d for C_13_H_17_N_2_O_2_S_2_ (M+H)^+^, 297.0726; found, 297.0729.

##### Synthesis of (2-(Thiophen-2-yl)thiazol-5-yl)methanamine Hydrochloride (**Amine 1a**)

To a solution of *tert*-butyl ((2-(thiophen-2-yl)thiazol-5-yl)methyl)carbamate (**Amine 1**) (200 mg, 0.7 mmol) in 1,4-dioxane (2 mL) was added hydrochloric acid (3.4 mL, 13.5 mmol) (4 M solution in 1,4-dioxane), and the reaction was stirred vigorously at rt for 16 h. Volatiles were removed *in vacuo* and dried under vacuum to afford the desired product (2-(thiophen-2-yl)thiazol-5-yl)methanamine hydrochloride (**Amine 1a**) (145 mg, 0.62 mmol, 92% yield) as a tan solid, which was carried forward as is to the next step without purification. ^1^H NMR (400 MHz, DMSO-*d*_6_): 8.67 (bs, 3H), 7.87 (s, 1H), 7.74 (dd, *J* = 5.0, 1.1 Hz, 1H), 7.67 (dd, *J* = 3.7, 1.0 Hz, 1H), 7.18 (dd, *J* = 5.0, 3.6 Hz, 1H), 4.31 (m, 2H). Note: Water peak observed at 3.62 ppm possibly due to presence of HCl. ^13^C NMR (100 MHz, DMSO-*d*_6_): 162.3, 144.7, 136.3, 130.1, 129.2, 128.6, 127.5, 34.4. HRMS (Q-TOF, ES+): calc’d for C_8_H_9_N_2_S_2_ (M+H)^+^, 197.0202; found, 197.0203.

##### Synthesis of *N*-((3-Bromo-1-methyl-1*H*-pyrazol-5-yl)methyl)-2-chloro-5-(furan-2-yl)benzamide (**17**)

HATU (86.8 mg, 0.23 mmol) was added to 2-chloro-5-(2-furyl)benzoic acid (**13**) (42 mg, 0.2 mmol), (5-bromo-2-methyl-pyrazol-3-yl)methanamine;dihydrochloride (50 mg, 0.2 mmol) and *N*,*N*-diisopropylethylamine (0.1 mL, 0.6 mmol) in DMF (1 mL) at RT then the solution was stirred at RT for 16 h. The solution was added dropwise into H_2_O (10 mL) with vigorous stirring. The resulting solids were collected by vacuum filtration, washed with H_2_O, and then dried under vacuum to give *N*-((3-bromo-1-methyl-1*H*-pyrazol-5-yl)methyl)-2-chloro-5-(furan-2-yl)benzamide (**17**) (62 mg, 83% yield) as an off-white solid. ^1^H NMR (400 MHz, CDCl_3_): 7.94 (d, *J* = 2.4 Hz, 1H), 7.67 (dd, *J* = 8.5, 2.6 Hz, 1H), 7.50 (d, *J* = 1.6 Hz, 1H), 7.42 (d, *J* = 8.6 Hz, 1H), 6.73 (d, *J* = 3.0 Hz, 1H), 6.63–6.58 (bs, 1H), 6.51 (dd, *J* = 3.0 Hz, 1.6 Hz, 1H), 6.28 (s, 1H), 4.69 (d, *J* = 6.4 Hz, 2H), 3.91 (s, 3H). ^13^C NMR (100 MHz, CDCl_3_): 166.0, 151.8, 142.9, 140.8, 134.3, 130.8, 130.2, 128.9, 126.7, 125.4, 124.7, 112.0, 108.7, 106.7, 37.1, 34.4. HRMS (Q-TOF, ES+): calc’d for C_16_H_14_BrClN_3_O_2_ (M+H)^+^, 393.9952; found, 393.9954.

### General Procedure 4

#### Synthesis of Analogs **19a**-**19d**

A vial was charged with *N*-((3-bromo-1-methyl-1*H*-pyrazol-5-yl)methyl)-2-chloro-5-(furan-2-yl)benzamide (**17**) (1.0 equiv), the respective boronic acid or boronic acid pinacol ester (1.5 equiv), BrettPhos Pd(II) G3 (0.1 equiv), potassium carbonate (2.5 equiv), and 5:1 DMA/H_2_O (0.08 M) The mixture was sparged with N_2_ for ∼5 min then heated to 90 °C for 2 h. Upon cooling to RT, the reaction mixture was filtered and then purified via reverse phase HPLC (Gilson 30 × 100 mm basic column: H_2_O with 0.05% NH_4_OH/MeCN: 5–70% MeCN gradient) to afford the desired products.

#### Synthesis of 2-Chloro-5-(furan-2-yl)-*N*-((1-methyl-3-(thiophen-2-yl)-1*H*-pyrazol-5-yl)methyl)benzamide (**19a**)

This compound was synthesized according to general procedure **4**. Off-white solid (2.4 mg, 12% yield). ^1^H NMR (400 MHz, CDCl_3_): 7.97 (d, *J* = 1.6 Hz, 1H), 7.67 (dd, *J* = 2.2, 8.7 Hz, 1H), 7.50 (d, *J* = 2.2 Hz, 1H), 7.42 (d, *J* = 8.7 Hz, 1H), 7.37 (d, *J* = 3.3 Hz, 1H), 7.26 (d, *J* = 4.9 Hz, 1H), 7.07–7.05 (m, 1H), 6.73 (d, *J* = 3.3 Hz, 1H), 6.69–6.64 (bs, 1H), 6.52–6.50 (m, 1H), 6.49 (s, 1H), 4.74 (d, *J* = 5.0 Hz, 2H), 3.99 (s, 3H).^13^C NMR (100 MHz, CDCl_3_): 166.2, 151.9, 145.4, 143.1, 140.4, 135.3, 134.2, 130.7, 130.2, 129.2, 127.6, 126.7, 125.3, 124.9, 124.1, 112.0, 106.9, 103.5, 36.9, 34.9. HRMS (Q-TOF, ES+): calc’d for C_20_H_17_ClN_3_O_2_S (M+H)^+^, 398.0725; found, 398.0726.

#### Synthesis of 2-Chloro-*N*-((3-(2-fluorophenyl)-1-methyl-1*H*-pyrazol-5-yl)methyl)-5-(furan-2-yl)benzamide (**19b**)

This compound was synthesized according to general procedure 4. Off-white solid (4.9 mg, 24% yield). ^1^H NMR (400 MHz, CDCl_3_): 8.05 (t, *J* = 8.0 Hz, 1H), 7.98 (d, *J* = 2.5 Hz, 1H), 7.69 (dd, *J* = 8.6, 1.5 Hz, 1H), 7.51 (d, *J* = 2.1 Hz, 1H), 7.43 (d, *J* = 8.1 Hz, 1H), 7.34–7.28 (m, 1H), 7.24–7.19 (m, 1H), 7.17–7.11 (m, 1H), 6.77 (d, *J* = 4.0 Hz, 1H), 6.74 (d, *J* = 3.5 Hz, 1H), 6.63–6.56 (bs, 1H), 6.52–6.49 (m, 1H), 4.83–4.77 (m, 2H), 4.08 (s, 3H). ^13^C NMR (100 MHz, CDCl_3_): 166.2, 160.2 (d, *J*_*CF*_ = 248 Hz), 151.9, 144.7, 143.1, 140.2, 134.6, 130.9, 130.4, 129.7 (d, *J*_*CF*_ = 9 Hz), 129.1, 128.5 (d, *J*_*CF*_ = 4 Hz), 126.8, 125.5, 124.6 (d, *J*_*CF*_ = 4 Hz), 120.2 (d, *J*_*CF*_ = 12 Hz), 116.2 (d, *J*_*CF*_ = 22 Hz), 112.2, 107.3 (d, *J*_*CF*_ = 10 Hz), 106.8, 37.1, 34.9. HRMS (Q-TOF, ES+): calc’d for C_22_H_18_ClFN_3_O_2_ (M+H)^+^, 410.1066; found, 410.1068.

#### Synthesis of 2-Chloro-5-(furan-2-yl)-*N*-((1-methyl-3-(pyridin-3-yl)-1*H*-pyrazol-5-yl)methyl)benzamide (**19c**)

This compound was synthesized according to general procedure 4. Off-white solid (2.1 mg, 11% yield). ^1^H NMR (400 MHz, CDCl_3_): 9.04–8.80 (bs, 1H), 8.51–8.31 (bs, 1H), 8.13 (d, *J* = 8.4 Hz, 1H), 7.92 (d, *J* = 2.2 Hz, 1H), 7.64 (dd, *J* = 8.8, 2.2 Hz, 1H), 7.48 (d, *J* = 1.8 Hz, 1H), 7.39 (d, *J* = 8.3 Hz, 1H), 7.37–7.31 (m, 2H), 6.70 (d, *J* = 4.0 Hz, 1H), 6.58 (s, 1H), 6.50–6.48 (m, 1H), 4.74 (d, *J* = 6.2 Hz, 2H), 3.97 (s, 3H).^13^C NMR (100 MHz, CDCl_3_): 166.4, 151.9, 147.0, 146.7, 145.5, 143.0, 140.6, 135.0, 133.8, 130.8, 130.2, 129.1, 126.5, 125.2, 124.3, 112.1, 106.7, 103.8, 37.2, 34.8. HRMS (Q-TOF, ES+): calc’d for C_21_H_18_ClN_4_O_2_ (M+H)^+^, 393.1113; found, 393.1120.

#### Synthesis of 2-Chloro-*N*-((1,2′-dimethyl-1*H*,2’*H*-[3,3′-bipyrazol]-5-yl)methyl)-5-(furan-2-yl)benzamide (**19d**)

This compound was synthesized according to general procedure 4. Off-white solid (2.9 mg, 15% yield). ^1^H NMR (400 MHz, DMSO-*d*_6_): 9.11 (t, *J* = 5.6 Hz, 1H), 7.79 (dd, *J* = 1.8, 0.7 Hz, 1H), 7.77–7.74 (m, 2H), 7.56–7.54 (m, 1H), 7.40 (d, *J* = 1.9 Hz, 1H), 7.11 (dd, *J* = 3.4, 0.8 Hz, 1H), 6.63 (dd, *J* = 3.4, 1.8 Hz, 1H), 6.59 (s, 1H), 6.50 (d, *J* = 1.9 Hz, 1H), 4.58 (d, *J* = 5.6 Hz, 2H), 4.04 (s, 3H), 3.91 (s, 3H). ^13^C NMR (100 MHz, DMSO-*d*_6_): 166.0, 151.3, 143.7, 140.8, 140.4, 137.8, 137.0, 135.9, 130.3, 129.2, 128.5, 125.4, 123.5, 112.4, 107.4, 105.2, 104.9, 38.5, 36.7, 33.8. HRMS (Q-TOF, ES+): calc’d for C_20_H_19_ClN_5_O_2_ (M+H)^+^, 396.1222; found, 396.1225.

### Scale-up Route for Accessing VU6024578 (19d)

#### Synthesis of 1,2′-Dimethyl-1*H*,2’*H*-[3,3′-bipyrazole]-5-carbaldehyde (**22**)

A RBF containing 1-methylpyrazole-5-boronic acid pinacol ester (**21**) (39.6 g, 190.4 mmol), 5-formyl-3-bromo-1-methylpyrazole (**20**) (30 g, 158.7 mmol), dichloro[1,1′-bis(diphenylphosphino)ferrocene]palladium(II) dichloromethane adduct (6.5 g, 7.9 mmol) and sodium carbonate (51.4 g, 476.1 mmol) in 1,4-dioxane (450 mL) and water (90 mL) was sparged with nitrogen and then stirred at 100 °C for 1 h. Upon cooling to rt, the reaction mixture was filtered through a pad of Celite, concentrated, and purified via flash chromatography (Teledyne ISCO flash purification system; silica gel column; hexane: EtOAc; 0–70% EtOAc gradient) to afford the desired product 1,2′-dimethyl-1*H*,2’*H*-[3,3′-bipyrazole]-5-carbaldehyde (**15**) (22.7 g, 119.3 mmol, 75% yield) as a colorless oil which solidified upon standing. ^1^H NMR (400 MHz, DMSO-*d*_6_): 9.95 (s, 1H), 7.45 (d, *J* = 1.9 Hz, 1H), 7.40 (s, 1H), 6.63 (d, *J* = 1.9 Hz, 1H), 4.17 (s, 3H), 4.06 (s, 3H). ^13^C NMR (100 MHz, DMSO-*d*_6_): 181.5, 141.2, 140.2, 138.0, 134.6, 111.7, 105.7, 38.9, 38.6. HRMS (Q-TOF, ES+): calc’d for C_9_H_11_N_4_O (M+H)^+^, 191.0927; found, 191.0928.

#### Synthesis of (1,2′-Dimethyl-1*H*,2’*H*-[3,3′-bipyrazol]-5-yl)methanamine Trihydrochloride (**23**)

Intermediate **22** (19.7 g, 103.6 mmol), hydroxylamine hydrochloride (7.6 g, 108.8 mmol) and sodium acetate (9.0 g, 108.8 mmol) were suspended in ethanol (500 mL). The mixture was sonicated and then stirred at rt for 30 min. The mixture was filtered through a pad of Celite before the sequential addition of palladium on activated carbon (1.84 g, 10.4 mmol) and conc. hydrochloric acid (17.4 mL, 208.3 mmol). The mixture was stirred vigorously under a H_2_ balloon (1 atm) at rt for 16 h. LCMS shows ∼95% conversion. The vessel was recharged with a fresh H_2_ balloon (1 atm), and the mixture was stirred vigorously at rt for an additional 4 h. The solution was then filtered through a pad of Celite. The filter cake was washed with 5:1 HCl (12 N aq): MeOH. The combined filtrates were then diluted with THF (3 volumes) and allowed to sit for 1 h. The resulting solids were collected via vacuum filtration (washing with additional THF) and then dried *in vacuo* to afford (1,2′-dimethyl-1*H*,2’*H*-[3,3′-bipyrazol]-5-yl)methanamine trihydrochloride (**23**) (11.9 g, 39.6 mmol, 38% yield) as a white powder. ^1^H NMR (400 MHz, DMSO-*d*_6_): 8.77 (bs, 3H), 7.45 (d, *J* = 2.0 Hz, 1H), 6.77 (s, 1H), 6.48 (d, *J* = 2.0 Hz, 1H), 4.19–4.14 (m, 2H), 4.03 (s, 3H), 3.94 (s, 3H). Note: A broad singlet was also observed at 6.10 ppm which could correspond to presence of residual water and HCl.^13^C NMR (100 MHz, DMSO-*d*_6_): 140.4, 137.8, 137.2, 135.7, 106.7, 105.0, 38.5, 37.1, 32.7. HRMS (Q-TOF, ES+): calc’d for C_9_H_14_N_5_ (M+H)^+^, 192.1244; found, 192.1246.

#### Synthesis of VU6024578 (**19d**)

1-(3-(dimethylamino)propyl)-3-ethylcarbodiimide hydrochloride (400 mg, 2.1 mmol) and 1-hydroxybenzotriazole hydrate (320 mg, 2.1 mmol) were added to 2-chloro-5-(furan-2-yl)benzoic acid (**13**) (370 mg, 1.7 mmol), (1,2′-dimethyl-1*H*,2’*H*-[3,3′-bipyrazol]-5-yl)methanamine trihydrochloride (**23**) (500 mg, 1.7 mmol) and *N,N*-diisopropylethylamine (1.2 mL, 6.7 mmol) in DMF (15 mL) at rt. The reaction was then stirred at rt for 2 h. The solution was added to H_2_O (100 mL) with stirring, and the mixture was then sonicated for 10 min. The resulting solids were collected via vacuum filtration (washing with H_2_O) then dried under vacuum to give the desired product **19d** (565 mg,1.4 mmol, 86% yield) as a white solid. The reaction was repeated using 2.80 and 6.39 g of benzoic acid **13** in two additional batches scaling from above. The combined reaction mixtures were poured slowly in to H_2_O (1000 mL) with vigorous stirring. The mixture was sonicated for 10 min, and then the resulting solids were collected via vacuum filtration (washing with H_2_O) and then dried under vacuum to afford 11.1 g of **19d** as a white solid (92% yield.) ^1^H NMR (400 MHz, DMSO-*d*_6_): 9.11 (t, *J* = 5.6 Hz, 1H), 7.79 (dd, *J* = 1.8, 0.7 Hz, 1H), 7.77–7.74 (m, 2H), 7.56–7.54 (m, 1H), 7.40 (d, *J* = 1.9 Hz, 1H), 7.11 (dd, *J* = 3.4, 0.8 Hz, 1H), 6.63 (dd, *J* = 3.4, 1.8 Hz, 1H), 6.59 (s, 1H), 6.50 (d, *J* = 1.9 Hz, 1H), 4.58 (d, *J* = 5.6 Hz, 2H), 4.04 (s, 3H), 3.91 (s, 3H). ^13^C NMR (100 MHz, DMSO-*d*_6_): 166.0, 151.3, 143.7, 140.8, 140.4, 137.8, 137.0, 135.9, 130.3, 129.2, 128.5, 125.4, 123.5, 112.4, 107.4, 105.2, 104.9, 38.5, 36.7, 33.8. HRMS (Q-TOF, ES+): calc’d for C_20_H_19_ClN_5_O_2_ (M+H)^+^, 396.1222; found, 396.1225.

## In vitro Pharmacology

### Molecular Pharmacology

Tetracycline-tested fetal bovine serum (FBS) was purchased from Atlanta Biologicals (Lawrenceville, GA), and all other tissue culture reagents and Fluo-4-acetoxymethylester (Fluo-4-AM) were purchased from Life Technologies (Carlsbad, CA). Tetracycline hydrochloride (Sigma), l-glutamic acid (Tocris, Minneapolis, MN), and (*S*)-3,5-dihydroxyphenylglycine (DHPG) (Abcam, Cambridge, MA). EZ-Link Sulfo-NHS-SS-Biotin and NeutrAvidin agarose beads (Pierce Biotechnology, Rockford, IL).

### Cell Culture and Mutagenesis

Tetracycline-inducible human mGlu_1_ WT-T-REx-293 cells (Wu et al, Science 2014) were cultured at 37 °C in Dulbecco’s Modified Eagle Medium (DMEM) growth medium containing 10% Tet-tested FBS, 2 mM l-glutamine, 20 mM HEPES, 0.1 mM nonessential amino acids, 1 mM sodium pyruvate, antibiotic/antimycotic, 100 μg/mL hygromycin and 5 μg/mL blasticidin in the presence of 5% CO_2_. To generate a collection of stable cell lines carrying tetracycline-inducible hmGlu_1_ schizophrenic mutants,^21,22^ site-directed mutagenesis of human mGlu_1_ WT in pcDNA5/TO was performed using Quikchange II XL kit (Agilent Technologies, Santa Clara, CA), and all point-mutations were confirmed by sequencing. The mutant stable cell lines were generated in the same manner as the WT as previously described and cultured in the growth medium described above.

### Calcium Mobilization Assay

To determine the potency of mGlu_1_ PAMs in calcium assays, Ca flux was measured as previously described in *ACS Chem. Bio*. **2014**, 9, 2334–2346. Briefly, the day before the assay, human mGlu_1_ WT-T-REx-293 cells were plated in black-walled, clear-bottomed, poly-d-lysine coated 96-well plates at 80,000 cells/100 μL assay medium (DMEM supplemented with 10% dialyzed FBS, 20 mM HEPES, and 1 mM sodium pyruvate) containing 50 ng/mL of tetracycline to induce mGlu_1_ expression. The next day, media was removed and the cells were incubated with 50 μL of 1.15 μM Fluo-4 AM dye solution prepared in assay buffer (buffer (Hank’s balanced salt solution, 20 mM HEPES, and 2.5 mM probenecid) for 45 min at 37 °C, the dye was removed and replaced with 45 μL of assay buffer. Then, calcium flux was measured using Flexstation II (Molecular Devices, Sunnyvale, CA). Compounds serially diluted at half log concentrations in DMSO were further diluted in assay buffer. The compounds or DMSO vehicle were added to cells and incubated for 2.5 min and an EC_20_ concentration of glutamate was added and incubated for 1 min. An E*C*_max_ concentration of glutamate was also added to cells that were incubated with DMSO vehicle to accurately calculate the EC_20_ calcium response. Data were normalized by subtracting the basal florescent peak before EC_20_ agonist addition from the maximal peak elicited by EC_20_ agonist and PAMs. Using GraphPad Prism 5.0, the concentration response curves were generated and the potencies of the mGlu_1_ PAMs were determined.

## DMPK Methods

### Plasma Protein Binding

The protein binding of each compound was determined in plasma via equilibrium dialysis employing RED Plates (ThermoFisher Scientific, Rochester, NY). Plasma was added to the 96 well plate containing test compound and mixed thoroughly for a final concentration of 5 μM. Subsequently, an aliquot of the plasma-compound mixture was transferred to the *cis* chamber (red) of the RED plate, with an phosphate buffer (25 mM, pH 7.4) in the *trans* chamber. The RED plate was sealed and incubated for 4 h at 37 °C with shaking. At completion, aliquots from each chamber were diluted 1:1 with either plasma (*cis*) or buffer (*trans*) and transferred to a new 96 well plate, at which time ice-cold acetonitrile containing internal standard (50 ng/mL carbamazepine) (2 volumes) was added to extract the matrices. The plate was centrifuged (3000 rcf, 10 min) and supernatants transferred and diluted 1:1 (supernatant: water) into a new 96 well plate, which was then sealed in preparation for LC/MS/MS analysis. Each compound was assayed in triplicate within the same 96-well plate. Fraction unbound was determined using the following equation
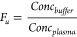


### Intrinsic Clearance

Human or rat hepatic microsomes (0.5 mg/mL) and 1 μM test compound were incubated in 100 mM potassium phosphate pH 7.4 buffer with 3 mM MgCl_2_ at 37 °C with constant shaking. After a 5 min preincubation, the reaction was initiated by addition of NADPH (1 mM). At selected time intervals (0, 3, 7, 15, 25, and 45 min), aliquots were taken and subsequently placed into a 96-well plate containing cold acetonitrile with internal standard (50 ng/mL carbamazepine). Plates were then centrifuged at 3000 rcf (4 °C) for 10 min, and the supernatant was transferred to a separate 96-well plate and diluted 1:1 with water for LC/MS/MS analysis. The *in vitro* half-life (*T*_1/2_, min, eq 1), intrinsic clearance (CL_int_, mL/min/kg, eq 2) and subsequent predicted hepatic clearance (CL_hep_, mL/min/kg, eq 3) was determined employing the following equations:
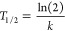
1where k represents the slope from linear regression analysis of the natural log percent remaining of test compound as a function of incubation time
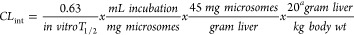
2

^a^scale-up factors: of 20 (human) or 45 (rat)
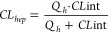
3where Q_h_ (hepatic blood flow, mL/min/kg) is 21 (human) or 70 (rat).

### LC/MS/MS Bioanalysis of Samples from Plasma Protein Binding and Intrinsic Clearance Assays

Samples were analyzed on a Thermo Electron TSQ Quantum Ultra triple quad mass spectrometer (San Jose, CA) via electrospray ionization (ESI) with two Themo Electron Accella pumps (San Jose, CA), and a Leap Technologies CTC PAL autosampler (Carrboro, NC). Analytes were separated by gradient elution on a dual column system with two Thermo Hypersil Gold (2.1 × 30 mm, 1.9 μm) columns (San Jose, CA) thermostated at 40 °C. HPLC mobile phase A was 0.1% formic acid in water and mobile phase B was 0.1% formic acid in acetonitrile. The gradient started at 10% B after a 0.2 min hold and was linearly increased to 95% B over 0.8 min; hold at 95% B for 0.2 min; returned to 10% B in 0.1 min. The total run time was 1.3 min and the HPLC flow rate was 0.8 mL/min. While pump 1 ran the gradient method, pump 2 equilibrated the alternate column isocratically at 10% B. Compound optimization, data collection and processing was performed using Thermo Electron’s QuickQuan software (v2.3) and Xcalibur (v2.0.7 SP1).

#### Inhibition of Cytochrome P450 Enzymes

A cocktail of substrates for cytochrome P450 enzymes (1A2: Phenacetin, 10 μM; 2C9: Diclofenac, 5 μM; 2D6: Dextromethorphan, 5 μM; 3A4: Midazolam, 2 μM) were mixed for cocktail analysis. For P450 2C19, the substrate stock (Mephenytoin, 40 μM)and substrate mix were prepared separately for discrete analysis. The positive control for pan-P450 inhibition (miconazole) was included alongside each test compound in analysis.

A reaction mixture of 100 mM Kpi, pH 7.4, 0.1 mg/mL human liver microsomes (HLM) and Substrate Mix is prepared and aliquoted into a 96-deepwell block. Test compound and positive control (in duplicate) were then added such that the final concentration of test compound ranged from 0.1–30 μM. The plate was vortexed briefly and then preincubated at 37 °C while shaking for 15 min. The reaction was initiated with the addition of NADPH (1 mM final concentration). The incubation continued for 8 min and the reaction quenched by 2x volume of cold acetonitrile containing internal standard (50 nM carbamazepine). The plate was centrifuged for 10 min (4000 rcf, 4 °C) and the resulting supernatant diluted 1:1 with water for LC/MS/MS analysis. A 12 point standard curve of substrate metabolites over the range of 0.98 nM to 2000 nM.

Samples were analyzed via electrospray ionization (ESI) on an AB Sciex API-4000 (Foster City, CA) triple-quadrupole instrument that was coupled with Shimadzu LC-10AD pumps (Columbia, MD) and a Leap Technologies CTC PAL autosampler (Carrboro, NC). Analytes were separated by gradient elution using a Fortis C18 3.0 × 50 mm, 3 μm column (Fortis Technologies Ltd., Cheshire, UK) thermostated at 40 °C. HPLC mobile phase A was 0.1% formic acid in water (pH unadjusted), mobile phase B was 0.1% formic acid in acetonitrile (pH unadjusted). The gradient started at 10% B after a 0.2 min hold and was linearly increased to 90% B over 1.2 min; held at 90% B for 0.1 min and returned to 10% B in 0.1 min followed by a re-equilibration (0.9 min). The total run time was 2.5 min and the HPLC flow rate was 0.5 mL/min. The source temperature was set at 500 °C and mass spectral analyses were performed using multiple reaction monitoring (MRM), with transitions specific for each compound utilizing a Turbo-Ionspray source in positive ionization mode (5.0 kV spray voltage).

The IC_50_ values for each compound were obtained for the individual CYP enzymes by quantitating the inhibition of metabolite formation for each probe substrate. A 0 μM compound condition (or control) was set to 100% enzymatic activity and the effect of increasing test compound concentrations on enzymatic activity could then be calculated from the % of control activity. Curves were fitted using XLfit 5.2.2 (four-parameter logistic model, eq 201) to determine the concentration that produces half-maximal inhibition (IC_50_).

## *In Vivo* DMPK Experimental

Compounds were formulated as 10% tween 80 micro suspensions in sterile water at the concentration of 1 mg/mL and administered intraperitoneally to male Sprague- Dawley rats weighing 225 to 250 g (Harlan, Inc., Indianapolis, IN) at the dose of 10 mg/kg. The rat blood and brain were collected at 0.25 h. Animals were euthanized and decapitated, and the brains were removed, thoroughly washed in cold phosphate buffered saline and immediately frozen on dry ice. Trunk blood was collected in EDTA Vacutainer tubes, and plasma was separated by centrifugation and stored at −80 °C until analysis. Plasma was separated by centrifugation (4000 rcf, 4 °C) and stored at 80 °C until analysis. On the day of analysis, frozen whole-rat brains were weighed and diluted with 1:3 (w/w) parts of 70:30 isopropanol:water. The mixture was then subjected to mechanical homogenation employing a Mini-Beadbeater and 1.0 mm Zirconia/Silica Beads (BioSpec Products) followed by centrifugation. The sample extraction of plasma (20 μL) or brain homogenate (20 μL) was performed by a method based on protein precipitation using three volumes of ice-cold acetonitrile containing an internal standard (50 ng/mL carbamazepine). The samples were centrifuged (3000 rcf, 5 min) and supernatants transferred and diluted 1:1 (supernatant: water) into a new 96 well plate, which was then sealed in preparation for LC/MS/MS analysis.

*In vivo* samples were analyzed via electrospray ionization (ESI) on an AB Sciex API-5500 QTrap (Foster City, CA) instrument that was coupled with Shimadzu LC-20AD pumps (Columbia, MD) and a Leap Technologies CTC PAL autosampler (Carrboro, NC). Analytes were separated by gradient elution using a Fortis C18 3.0 × 50 mm, 3 μm column (Fortis Technologies Ltd., Cheshire, UK) thermostated at 40 °C. HPLC mobile phase A was 0.1% formic acid in water (pH unadjusted), mobile phase B was 0.1% formic acid in acetonitrile (pH unadjusted). The gradient started at 30% B after a 0.2 min hold and was linearly increased to 90% B over 0.8 min; held at 90% B for 0.5 min and returned to 30% B in 0.1 min followed by a re-equilibration (0.9 min). The total run time was 2.5 min and the HPLC flow rate was 0.5 mL/min. The source temperature was set at 500 °C and mass spectral analyses were performed using multiple reaction monitoring (MRM), with transitions specific for each compound utilizing a Turbo-Ionspray source in positive ionization mode (5.0 kV spray voltage). The calibration curves were constructed in blank plasma. All data were analyzed using AB Sciex Analyst software v1.5.1.

## Animal Care and Use

All animal study procedures were approved by the Institutional Animal Care and Use Committee and were conducted in accordance with the National Institutes of Health regulations of animal care covered in Principles of Laboratory Animal Care (National Institutes of Health).

## qEEG Recordings Procedure

Wistar rats using a telemetry system (Data Sciences International DSI, USA).^20^ Telemetric transmitters (F40-EET, Data Sciences International DSI, USA) were implanted intraperitoneally into male Wistar rats. EEG electrodes were placed supradurally above the hippocampus and two more electrodes were implanted into the neck muscle in order to measure the electromyography (EMG) signal required for differentiation between different vigilance states. Telemetric EEG recordings were performed during the light phase in the rats’ home cage environment. EEG activity was sampled at 500 Hz with a hardware filter cutoff of 50 Hz. Data were recorded continuously for 24 h, but only 1 h before compound treatment (baseline), the first 4 h after compound treatment plus an additional hour (+24 h time point) have been analyzed. Effects of treatment with **19d** (1, 10 mg/kg, p.o., n = 14, applied 2 h after starting the recording) were determined on motor activity, body temperature, vigilance states and EEG power spectra using the EEG analysis software NeuroScore (Data Sciences International DSI, USA). The rats were tested in a crossover study design with a two-day washout period between two treatments.^20^

## ERP Recordings Procedure

Male C57BL/6JRj mice (12–14 weeks old, 28–30 g, n = 13–19 per treatment arm) (Janvier Laboratories, Le Genest-Saint-Isle, France) were group housed prior to surgery (four animals per cage) and single housed after surgery under standard light-dark conditions (12 h/12 h, lights on 06:00–18:00) with food and tap water available ad libitum. C57BL/6JRj mice were used as they are well suited for neurophysiological cortical network investigations using EEG techniques (Schuelert et al., 2018). Five epidural EEG electrodes were placed 10 days prior to recording (PFC: anterior posterior (AP) 511.7 mm, medial lateral (ML): ± 1.0 mm; auditory cortex (AC): AP 5 2.7 mm, ML: ± 4.0 mm; reference electrode placement: AP 5 5.7 mm, ML: ± 0 mm).Blood samples were collected from satellite mice (n = 5 per dose group) to measure plasma drug exposure 60 min after subcutaneous administration.

Mice were placed in a plexiglass cage in an attenuated sound box and allowed to adapt to their environment for 20 min before subcutaneous administration of VU6024578 0.8, 4, and 10 mg/kg or vehicle (20% HP-β-CD in water). VU6024578 was dissolved in 20% HPβ-CD in water and then sonicated for 10 min, resulting in a clear solution. As a positive control the mGluR2/3 agonist LY379268 was administered subcutaneously at a dose of 3 mg/kg. After 15 min, all animals then received a subcutaneous dose of the MK-8010.15 mg/kg or vehicle (saline). All injections had a total volume of 10 mL/kg. A crossover treatment schedule was used such that each mouse received all treatment conditions separated by a 3-day washout period. Recordings in PFC and AC began 15 min after administration of either MK-801 or vehicle, and were conducted as described previously (Schuelert et al., 2018). Auditory stimuli were generated with an audio generator (MED Associates Inc.) and presented via a house speaker system in each of the sound-attenuated boxes (MED Associates Inc.). Using MED-specific software, the auditory stimulation protocols were programmed and a TTL signal was sent simultaneously with each tone to activate the LED infrared lights. An infrared sensor on the Neurologger recorded the infrared trigger signals and trigger signals were saved in an individual trigger channel. An ASSR session consisted of periodic train of single white noise clicks in a frequency of 40 Hz. Each train lasted 2 s with an interval of 10 s in between click trains. A total of 300 trains were presented. The intensity of the 40 Hz click train was adjusted to be 85 ± 1.0 dB. Sensory gating was assessed with a paired-click paradigm (Schuelert et al. 2008). 300 pairs of white noise clicks where presented with an interstimulus interval of 0.5 s between clicks and an interval of 8 s between each double-click.

## ERP Analysis

EEG parameters were analyzed as previously described (Schuelert et al., 2018).^27^ Briefly, four recording channels (one AC channel and one PFC channel for each hemisphere) and one trigger channel (to identify the timing of tone presentations) were analyzed per animal. The sampling rate of recording channels and trigger channels was set to 1000 Hz. Any EEG recording segments with artifacts were removed. Analyzer 2 software (Brain Products GmbH, Munich, Germany) was used for data analysis. Sensory gating of N1 amplitude between paired click stimuli was measured using the ratio between the N1 amplitude of click 1 and click 2 to evaluate the capacity of the sensory system to filter redundant stimuli. ASSR intertrial coherence (ITC) was assessed by presentation of a click train 2 s in length with a frequency of 40 Hz. The ASSR paradigm explores cortical capacity to support an entrained 40 Hz oscillation.

## Catalepsy

Adult male Sprague–Dawley rats were administered VU6024578 (1–10 mg/kg p.o.) or vehicle and returned to their home cage for 60 min. Control rats were injected with 1.5 mg/kg of haloperidol i.p. (dissolved in 8% lactic acid and taken to volume of 50 mL with sterile water) and were returned to their home cage for 90 min. Cataleptic behavior is determined by placing the forelimbs on a bar raised 6 cm above the table and recording the amount of time it takes for the rat to withdraw the forelimbs with a cutoff of 60 s. Data are expressed as mean latency to withdraw + SEM or percent inhibition of catalepsy + SEM.

## Rotarod

On the first day, the animals were placed on the rotarod apparatus which was rotating at a speed of 20 rpm. If the rats fall of the bar, the animals were placed back on the apparatus for a total of 2 min. On the day of testing, adult male rats were pretreated with VU6024578 (1–10 mg/kg) 65 min before being placed on the rotarod apparatus. Testing consisted of accelerating rotarod from 4 to 40 rpm over 5 min. The time and final speed when the rats fall off of the apparatus was recorded.

## Spontaneous Locomotion

Adult male Sprague–Dawley rats were administered VU6024578 (1–10 mg/kg p.o.) or vehicle and were tested in the rotorod and catalepsy assays. Seventy minutes later the animals were placed into the activity chambers (Med Associates) for 30 min.

## Data Analysis

The data for the studies were analyzed by a between-group 2 way analysis of variance for compound dose and time. Each dose group was compared with the vehicle control group. Total ambulation rotorod and catalepsy data was analyzed by one-way ANOVA. Each dose group was compared with the vehicle control group. The calculations were performed and graphed using GraphPad Prism (version 4.03, GraphPad, La Jolla, CA).

## Amphetamine-Induced Hyperlocomotion (Mouse)

Adult male C57/bl6 mice are placed in the activity chambers (Med Associates) to habituate. 90 min later the mice were administered VU6024578 (3–30 mg/kg i.p. Ten ml/kg) or vehicle (0.5% natrasol/0.015% Tween 80) and placed back in the activity chambers. Thirty minutes later the animals were administered Amphetamine (3 mg/kg) and placed back in the activity chambers for 1 h. The data for the studies were analyzed by a between-group 2 way analysis of variance for compound dose and time. Total ambulation from 125 to 180 min was analyzed by one-way ANOVA. Each dose group was compared with the vehicle control group. The calculations were performed and graphed using GraphPad Prism (version 4.03, GraphPad, La Jolla, CA).

## Amphetamine-Induced Hyperlocomotion (Rat)

Adult male Sprague–Dawley rats placed into the activity chambers (Med Associates). Thirty minutes after placement in the chambers, the rats were administered VU6024578 (1–10 mg/kg p.o.) or vehicle. Sixty minutes later the rats were injected with amphetamine (0.75 mg/kg s.c. One ml/kg) or vehicle (saline) and placed back in the activity chambers for 90 min. The data for the studies were analyzed by a between-group 2 way analysis of variance for compound dose and time. Each dose group was compared with the vehicle control group. The calculations were performed and graphed using GraphPad Prism (version 4.03, GraphPad, La Jolla, CA). The AUC was analyzed by a 1-way ANOVA with a Dunnett’s post hoc test.

## Novel Object Recognition

The animals are habituated to the novel object arenas for 1 day prior to testing. This is accomplished by placing the animal in the arena with no objects for 10 min and then placed back into the home cage. The arena is then cleaned with 70% ethanol in between animals. The day of the test the animals are habituated to the dosing room for 2 h. The animals were administered VU6024578 (1–10 mg/kg p.o.) or vehicle (10% Tween 80) and were placed back into their homecage for 60 min. The animals were then injected with MK-801 (0.075 mg/kg 1 mL/kg s.c.) and placed back in the homecage for 30 min. The animals are then placed into the arena with two identical familiar objects for 10 min and then placed back into the home cage. The animals are then placed back into the arena with one of the familiar objects replaced with a novel object at 120 min after the familiar object exposure. The activity of the animals are recorded and the time spent exploring each of the objects is scored by a blinded observer. Recognition index is determined by subtracting the time spent with the novel object form the time spent with the familiar object divided by total time spent exploring.

## Supporting Information Available

Additional experimental details, methods for the synthesis and characterization of all compounds, *in vitro* and *in vivo* DMPK protocols, supplemental figures and molecular string formulas (PDF)
SMILES notation of compounds (CSV)

## ABBREVIATIONS

AHL: amphetamine-induced hyperlocomotion
DMPK: drug metabolism and pharmacokinetics
AE: adverse event
MED: minimum effective dose
PAM: positive allosteric modulator
PBL: plasma/brain level
mGlu_1_: metabotropic glutamate receptor subtype 1
NOR: novel object recognition
