# Supplementary material for: Discovery of VU6024578/BI02982816: An mGlu1 Positive Allosteric Modulator with Efficacy in Preclinical Antipsychotic and Cognition Models
Source: J Med Chem. 2024 Dec 12;67(24):22291–312. doi: 10.1021/acs.jmedchem.4c02554 (PMC11684029; doi:10.1021/acs.jmedchem.4c02554)

Supporting Information

Discovery of VU6024578/BI02982816: an mGlu$_1$ Positive Allosteric Modulator (PAM) with efficacy in preclinical antipsychotic and cognition models

Carson W. Reed, Jacob F. Kalbfleisch, Jeremy A. Turkett, Trevor A. Trombley, Anthony F. Nastase, Paul K. Spearing, Daniel H. Haymer, Mohammad Moshin Sarwar, Marc Quitalig, Jonathan W. Dickerson, Annie L. Blobaum, Olivier Boutaud, Patrizia Voehringer, Niklas Schuelert, Hyekyung P. Cho, Colleen M. Niswender, Jerri M. Rook, Henning Priepke, Daniel Ursu, P. Jeffrey Conn, Bruce J. Melancon and Craig W. Lindsley*

Table of Contents

Table S1. Eurofins lead profiling screen data…………………………………...…………......S2

Figure S1. Human and rat liver microsome incubation of **19d** with 20 μM glutathione……....S6

Figure S2. Exposure of **19d** and its effect in rat locomotor battery assays……………………..S7

Table S2. Satellite PK exposure of **19d** in MK-801 induced disruption of rat NOR…………...S8

Table S3. Satellite PK exposure of **19d** in rat AHL assay………………………………………S8

Table S4. Satellite PK exposure of **19d** in mouse AHL assay…………………………………..S8

Figure S3. Rat PO PK PBL snapshot of **19d**…………………………………………………S9

HPLC Traces for Final Compounds……………………………………………………………S10

^{1}H NMR and ^{13}C NMR Spectra for Final Compounds………………………………………S21

Table S1. Eurofins Lead Profiling Screen Data

This is a radioligand binding panel of 80 targets including GPCRs, ion channels, transporters and nuclear hormones. Biochemical assay results are presented as the percent inhibition of specific binding at a 10 µM concentration of **VU6024578** (Compound **19d**). Results showing an inhibition or stimulation higher than 50% are considered to represent significant effect and are highlighted in the table below.

Target / Protein	Species	% Inhibition at 10 µM
Adenosine A1 (antagonist radioligand)	human	8
Adenosine A2A (agonist radioligand)	human	-1
Adenosine A3 (agonist radioligand)	human	23
Adrenergic alpha 1 (non-selective) (antagonist radioligand)	rat	19
Adrenergic alpha 2 (non-selective) (antagonist radioligand)	rat	1
Adrenergic beta 1 (agonist radioligand)	human	1
Adrenergic beta 2 (antagonist radioligand)	human	-1
AT1 (antagonist radioligand)	human	-1
AT2 (agonist radioligand)	human	2
BZD (central) (agonist radioligand)	-	5
BZD (peripheral) (antagonist radioligand)	-	96
BB (non-selective) (agonist radioligand)	rat	-20
B2 (agonist radioligand)	human	7
CGRP (agonist radioligand)	human	1
CB1 (agonist radioligand)	human	0
CCK1 (CCKA) (agonist radioligand)	human	7
CCK2 (CCKB) (agonist radioligand)	human	-4

D1 (antagonist radioligand)	human	3
D2S (antagonist radioligand)	human	6
D3 (antagonist radioligand)	human	-6
D4.4 (antagonist radioligand)	human	22
D5 (antagonist radioligand)	human	-1
ETA (agonist radioligand)	human	-6
ETB (agonist radioligand)	human	-27
GABA (non-selective) (agonist radioligand)	rat	-3
GAL1 (agonist radioligand)	human	-4
GAL2 (agonist radioligand)	human	-9
PDGF (agonist radioligand)	mouse	-2
CXCR2 (IL-8B) (agonist radioligand)	human	3
CCR1 (agonist radioligand)	human	-1
TNF-alpha (agonist radioligand)	human	2
H1 (antagonist radioligand)	human	6
H2 (antagonist radioligand)	human	-31
MC4 (agonist radioligand)	human	14
MT1 (ML1A) (agonist radioligand)	human	17
M1 (antagonist radioligand)	human	14
M2 (antagonist radioligand)	human	18
M3 (antagonist radioligand)	human	2
M4 (antagonist radioligand)	human	11
M5 (antagonist radioligand)	human	3
NK1 (agonist radioligand)	human	46

NK2 (agonist radioligand)	human	11
NK3 (antagonist radioligand)	human	-8
Y1 (agonist radioligand)	human	-7
Y2 (agonist radioligand)	human	-18
NTS1 (NT1) (agonist radioligand)	human	-1
delta (DOP) (agonist radioligand)	human	8
kappa (KOP) (agonist radioligand)	human	11
mu (MOP) (agonist radioligand)	human	14
NOP (ORL1) (agonist radioligand)	human	-1
PAC1 (PACAP) (agonist radioligand)	human	-2
PPARgamma (agonist radioligand)	human	2
PCP (antagonist radioligand)	rat	2
EP2 (agonist radioligand)	human	41
EP4 (agonist radioligand)	human	62
IP (PGI2) (agonist radioligand)	human	-7
P2X (agonist radioligand)	-	-6
P2Y (agonist radioligand)	rat	23
5-HT1A (agonist radioligand)	human	51
5-HT1B (antagonist radioligand)	human	11
5-HT2A (antagonist radioligand)	human	-1
5-HT2B (agonist radioligand)	human	67
5-HT2C (antagonist radioligand)	human	3
5-HT3 (antagonist radioligand)	human	2
5-HT5a (agonist radioligand)	human	-2

5-HT6 (agonist radioligand)	human	-7
5-HT7 (agonist radioligand)	human	-4
sigma (non-selective) (agonist radioligand)	human	-11
sst (non-selective) (agonist radioligand)	mouse	-12
GR (agonist radioligand)	human	0
VPAC1 (VIP1) (agonist radioligand)	human	-4
V1a (agonist radioligand)	human	9
Ca2+ channel (L, verapamil site) (phenylalkylamine) (antagonist radioligand)	rat	-16
KV channel (antagonist radioligand)	rat	8
SKCa channel (antagonist radioligand)	rat	2
Na+ channel (site 2) (antagonist radioligand)	-	14
Cl- channel (GABA-gated) (antagonist radioligand)	-	9
norepinephrine transporter (antagonist radioligand)	human	11
dopamine transporter (antagonist radioligand)	human	37
5-HT transporter (antagonist radioligand)	human	47

In Vitro Glutathione Conjugates of 19d

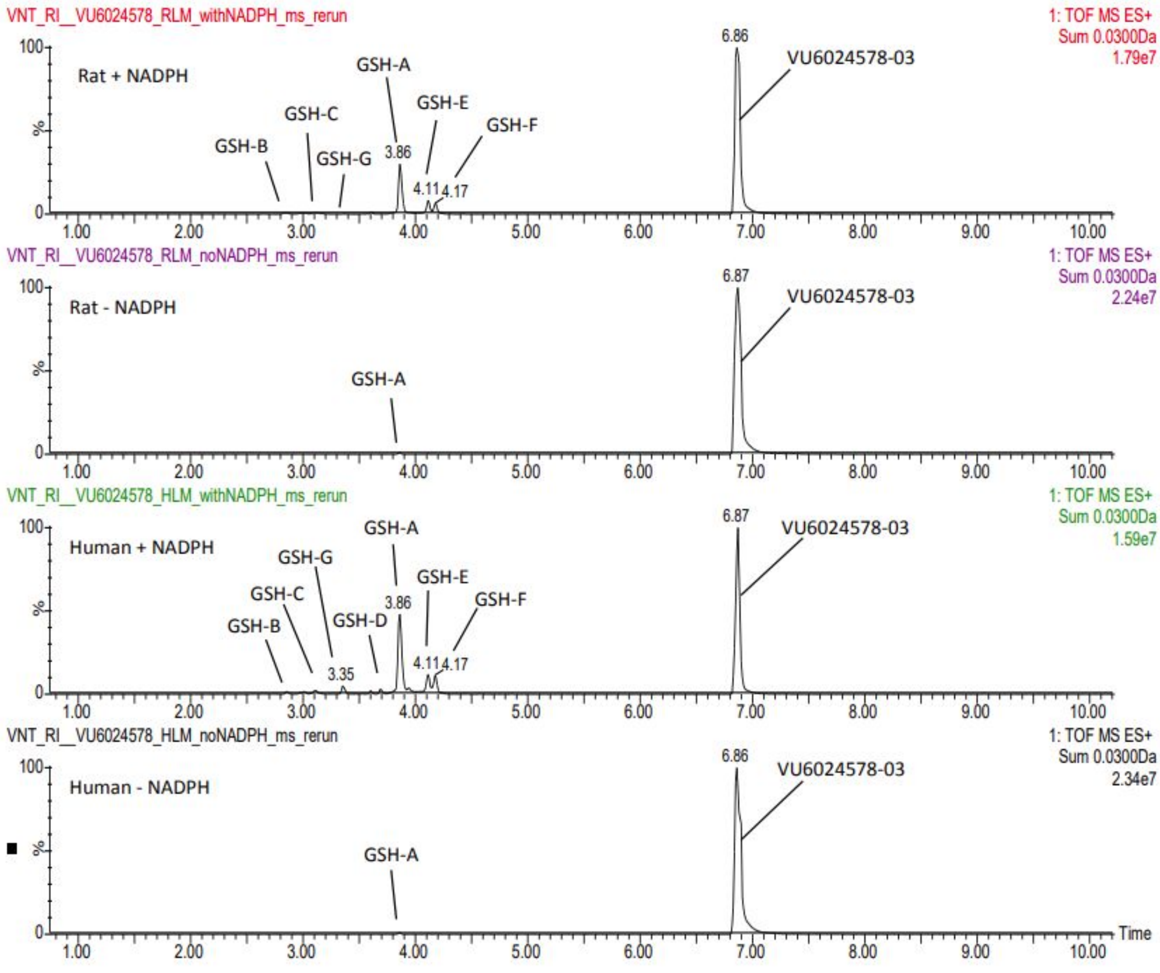

Peak ID	Tentative Metabolite Identification	Ion Found *m/z*	Retention Time (min)	Species/Matrix
VU6024578-03	Parent (P)	396.1232	6.87	RLM, HLM
GSH-A	P + cysteinylglycine – 2 H	572.1491	3.86	RLM, HLM
GSH-B	P + O + 2 H + glutathione	721.2158	2.85	RLM, HLM
GSH-C	P + O + 2 H + glutathione	721.2160	3.10	RLM, HLM
GSH-D	P + cysteinylglycine	574.1636	3.69	HLM
GSH-E	P + cysteinylglycine – 4 H	570.1345	4.11	RLM, HLM
GSH-F	P + cysteinylglycine – 4 H	570.1337	4.17	RLM, HLM
GSH-G	P + O + cysteinylglycine – 2 H	588.1435	3.35	RLM, HLM

RLM – rat liver microsomes, HLM – human liver microsomes

Figure S1. 19d was incubated in rat and human liver microsomes for 1 hr (with and without NADPH) with 20 μM glutathione. Extracted ion chromatograms revealed multiple glutathione conjugates, most likely due to reactive intermediate formation from the 2-furanyl southern heterocycle.

A

mGlu1 PAM Rat Locomotor Battery Dose Range (PO, 100 min post-dose)							
VU#	Dose (mg/kg)	Plasma (ng/mL)	Plasma Total (μM)	Plasma Free (nM)	Brain (ng/g)	Brain Total (μM)	Brain Free (nM)
VU6024578	1	331	0.84	34.3	358	0.90	30.8
	3	1187	3.00	123	851	2.15	73.1
	5.6	1795	4.54	186	1347	3.40	116
	10	2102	5.31	218	1561	3.94	134

B

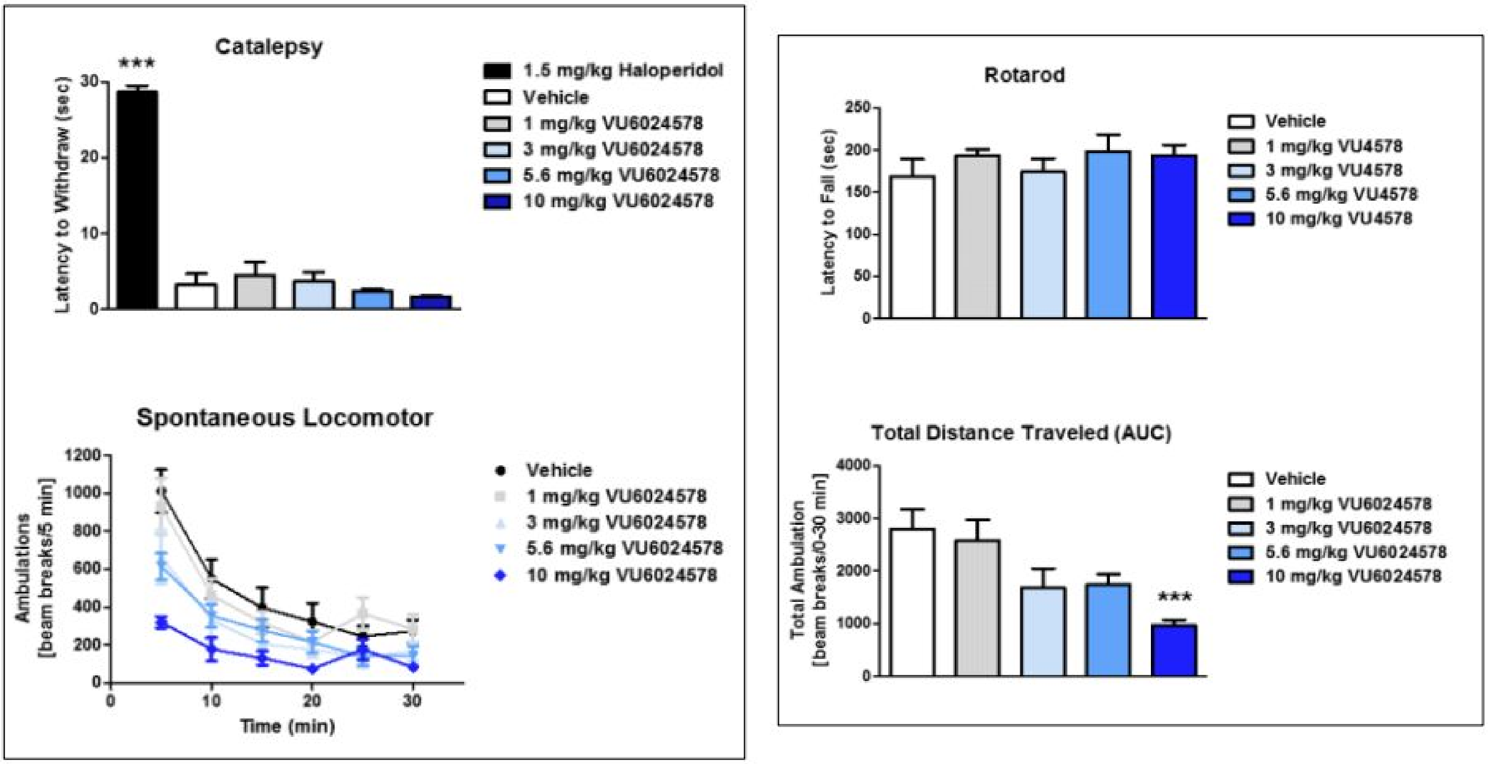

Figure S2. 19d assessed in rat locomotor battery **A:** Exposure of **19d** in the locomotor battery experiment. **B:**Doses of **19d** up to 10 mg/kg showed lack of effect in catalepsy, spontaneous locomotion, and rotarod assays.

mGlu1 PAM Rat PO Rat MK-801 HL Dose Response (Sample @ 4 hr)							
VU#	Dose (mg/kg)	Plasma (ng/mL)	Plasma Total (µM)	Plasma Free (nM)	Brain (ng/g)	Brain Total (µM)	Brain Free (nM)
VU6024578	1	258	0.65	26.7	145	0.37	12.5
	3	1074	2.71	111	352	0.89	30.2
	5.6	1234	3.12	128	487	1.23	41.8
	10	2501	6.32	259	847	2.14	72.8

Table S2. Satellite PK exposure of **19d** in rat MK-801 induced disruption of novel object recognition assay.

Measured conc. [nM]		
Dose (mg/kg)	Pretreatment Time (hr)	Brain Unbound (nM) @EOT
1	2.5	16.9
3	2.5	31.9
5.6	2.5	50.5
10	2.5	66.7

Table S3. Satellite PK exposure (unbound brain concentration at 2.5 hr EOT) of **19d** dosed PO in rat AHL assay.

VU6024578-09 Mouse AHL after IP administration							
VU#	Dose (mg/kg)	Plasma (ng/mL)	Plasma Total (µM)	Plasma Free (nM)	Brain (ng/g)	Brain Total (µM)	Brain Free (nM)
VU6024578-09	3,PO	53	0.13	8	34	0.08	7
	10, PO	245	0.62	36	174	0.44	36
	30, PO	749	1.89	110	329	0.83	67

Table S4. Satellite PK exposure of 19d in mouse AHL

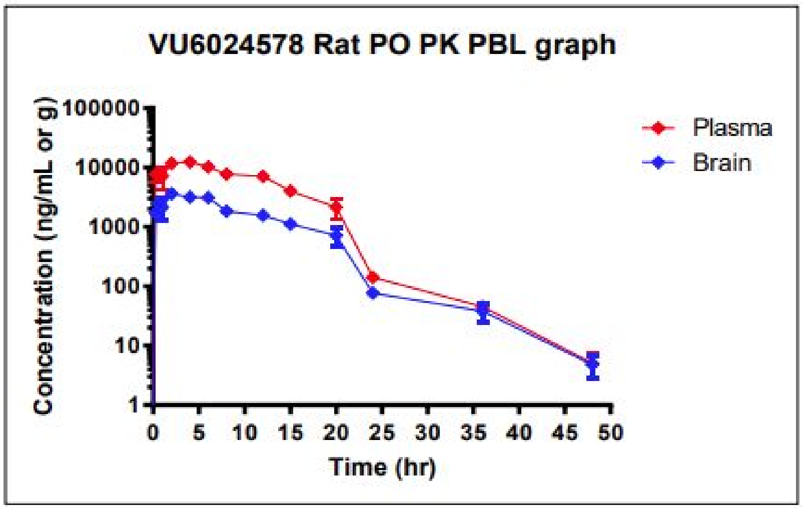

mGlu1 PAM PO PK and PBL Snapshot								
VU#	Time Point	Plasma (ng/mL)	Plasma Total (µM)	Plasma Unbound (nM)	Brain (ng/g)	Brain Total (µM)	Brain Unbound (nM)
VU6024578-06	0.25	7687	19.42	796	1721	4.35	148
	0.5	7627	19.27	790	2509	6.34	216
	1	7273	18.38	753	2146	5.42	184
	2	11800	29.8	1222	3645	9.21	313
	4	12400	31.3	1284	3192	8.06	274
	6	10230	25.8	1060	3115	7.87	268
	8	7770	19.6	805	1837	4.64	158
	12	7100	17.9	735	1561	3.94	134
	15	4020	10.2	416	1121	2.83	96.3
	20	2148	5.43	223	716	1.81	61.5
	24	142	0.36	14.7	76.5	0.19	6.57
	36	45.2	0.11	4.68	38.1	0.10	3.27
	48	5.07	0.01	0.53	4.81	0.01	0.41

Figure S3. Rat PO PK PBL snapshot with **19d.** Rat received **19d** (60 mg/kg PO, 10 ml/kg, 10% tween 80) and plasma and brain were obtained at 0.25. 0.5, 1, 2, 4, 6, 8, 12, 15, 20, 24, 36 and 48 hours post administration.

HPLC Traces for Final Compounds

Compound 6

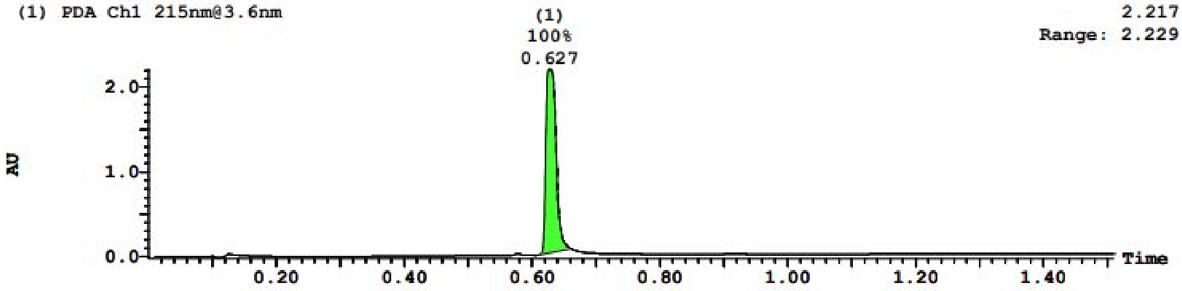

Peak Number	Time	Area %Total	BPM	Mass Found
1	0.627	100.00	316	315.0000

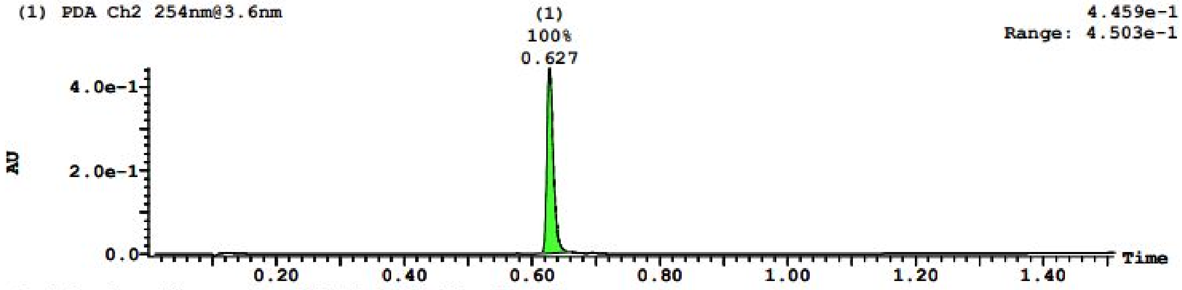

Peak Number	Time	Area %Total	BPM	Mass Found
1	0.627	100.00	316	315.0000

Compound 9a

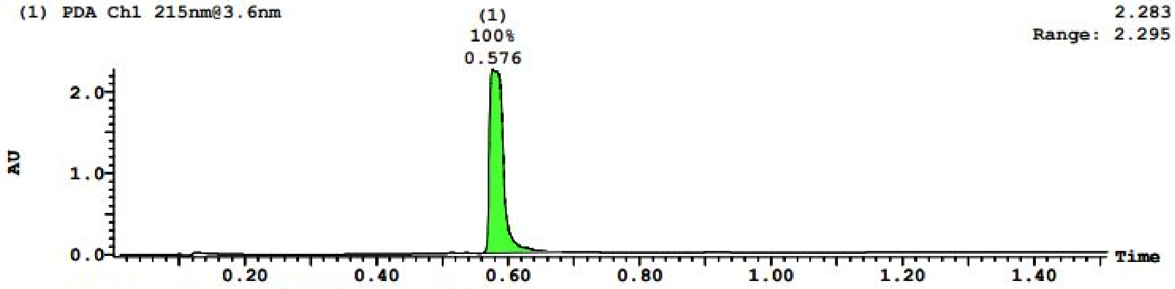

Peak Number	Time	Area %Total	BPM	Mass Found
1	0.576	100.00	312	311.0000

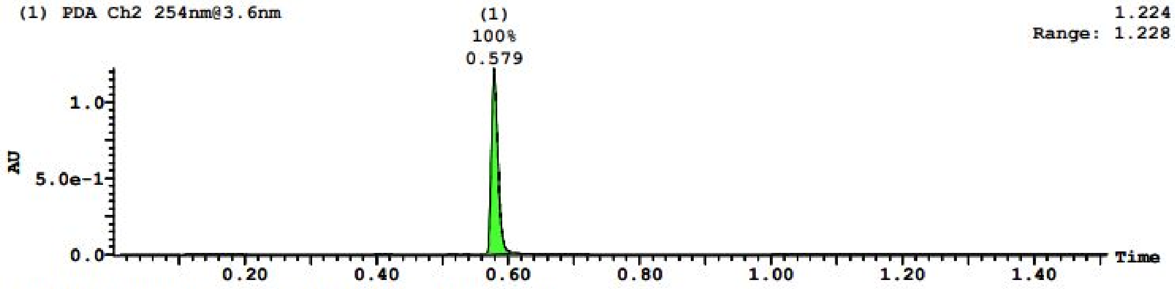

Peak Number	Time	Area %Total	BPM	Mass Found
1	0.579	100.00	312	311.0000

Compound 9b

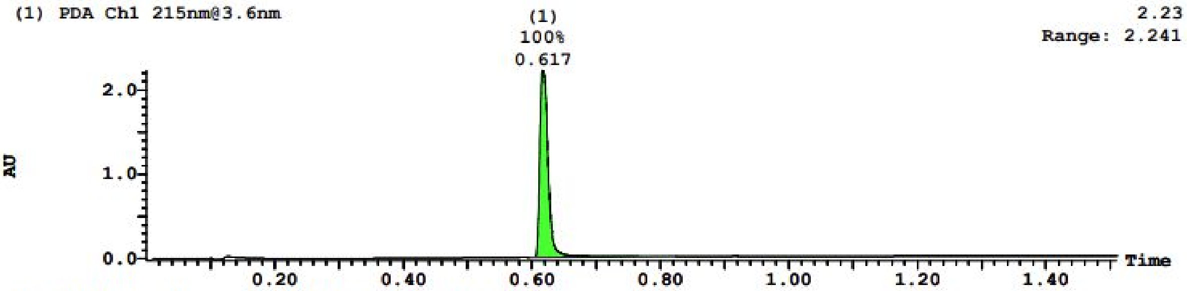

Peak Number	Time	Area %Total	BPM	Mass Found
1	0.617	100.00	296	295.0000

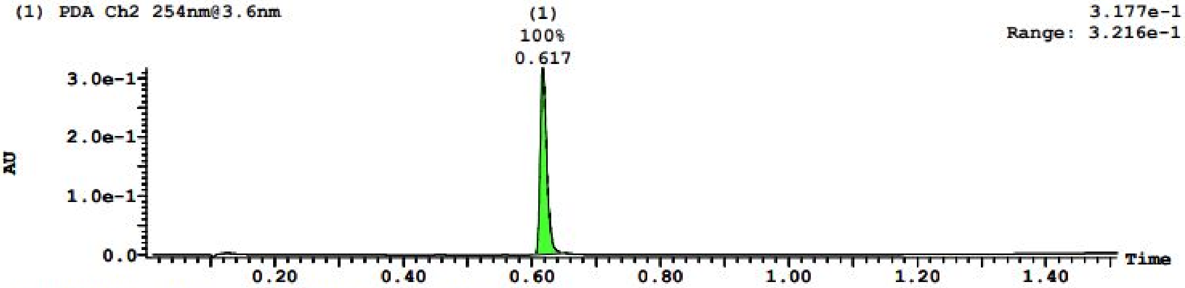

Peak Number	Time	Area %Total	BPM	Mass Found
1	0.617	100.00	296	295.0000

Compound 9c

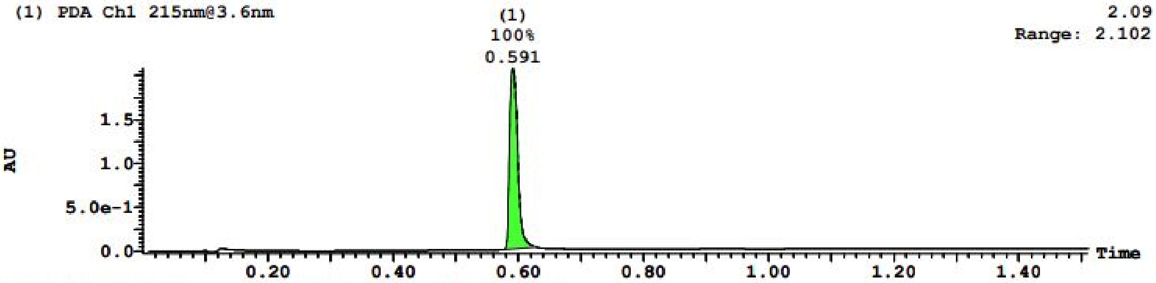

Peak Number	Time	Area %Total	BPM	Mass Found
1	0.591	100.00	300	299.0000

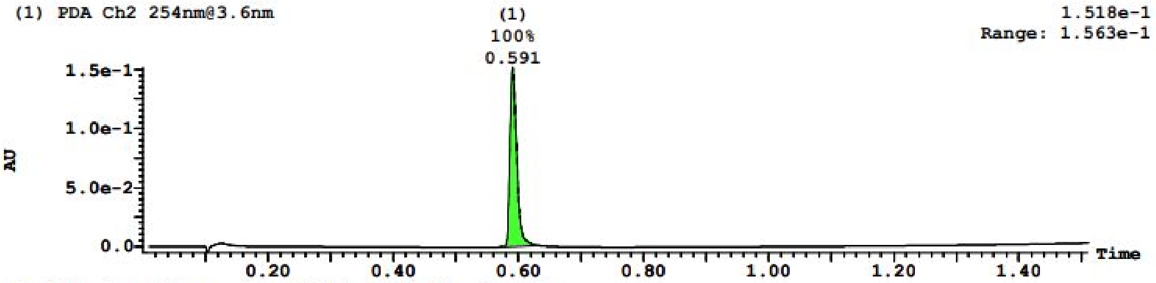

Peak Number	Time	Area %Total	BPM	Mass Found
1	0.591	100.00	300	299.0000

Compound 9d

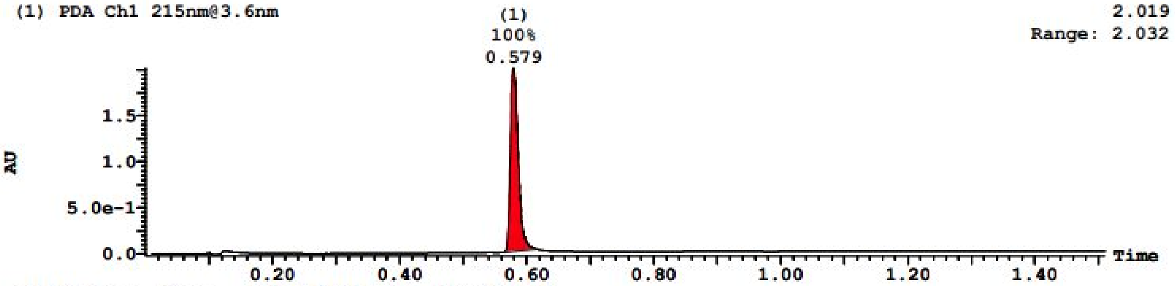

Peak Number	Time	Area %Total	BPM	Mass Found
1	0.579	100.00	320	Not Found

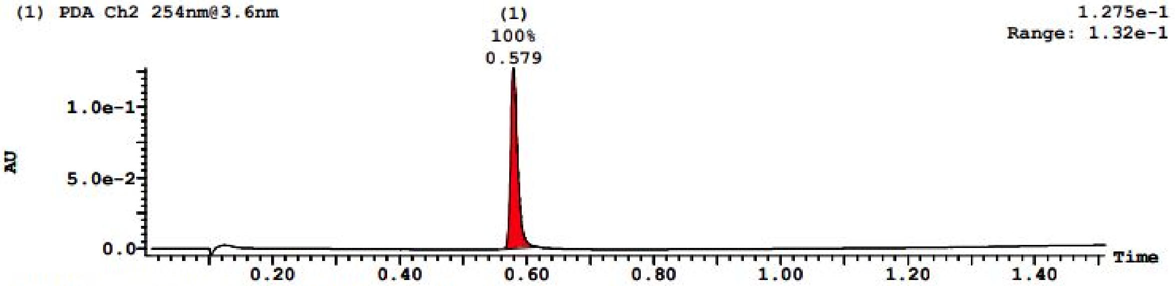

Peak Number	Time	Area %Total	BPM	Mass Found
1	0.579	100.00	320	Not Found

Compound 9f

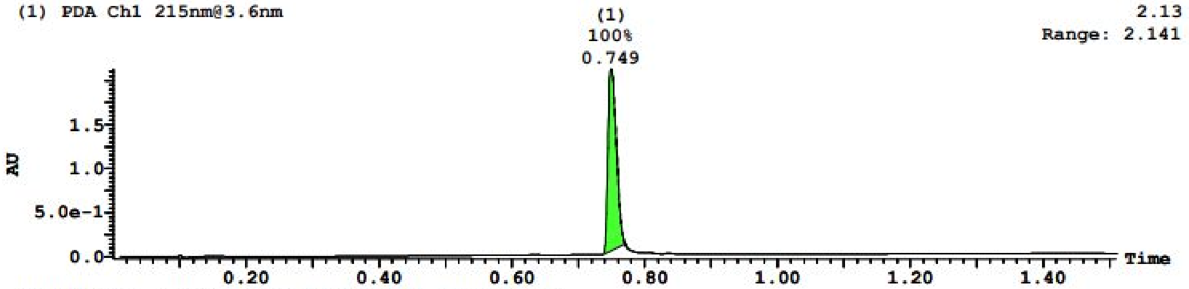

Peak Number	Time	Area %Total	BPM	Mass Found
1	0.749	100.00	362	361.0000

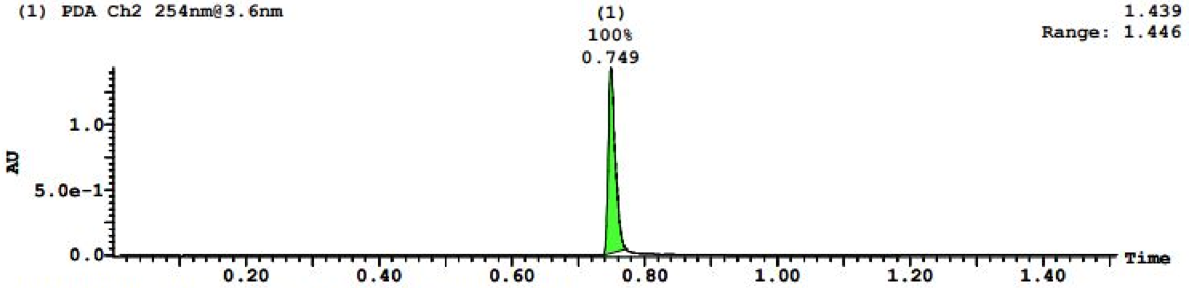

Peak Number	Time	Area %Total	BPM	Mass Found
1	0.749	100.00	362	361.0000

Compound 9g

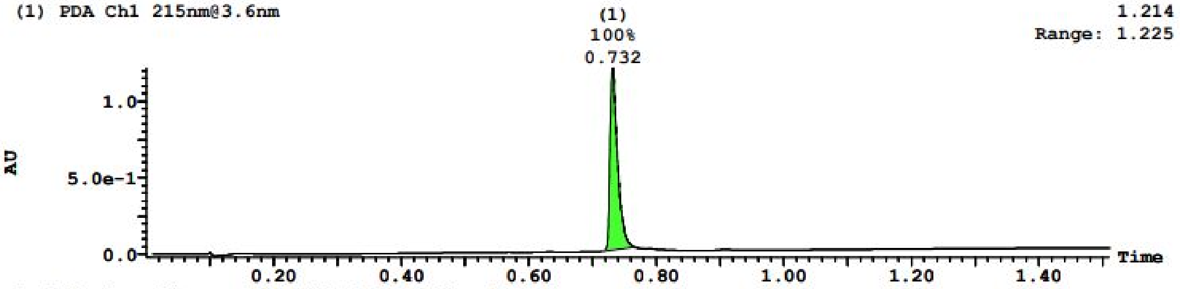

Peak Number	Time	Area %Total	BPM	Mass Found
1	0.732	100.00	368	367.0000

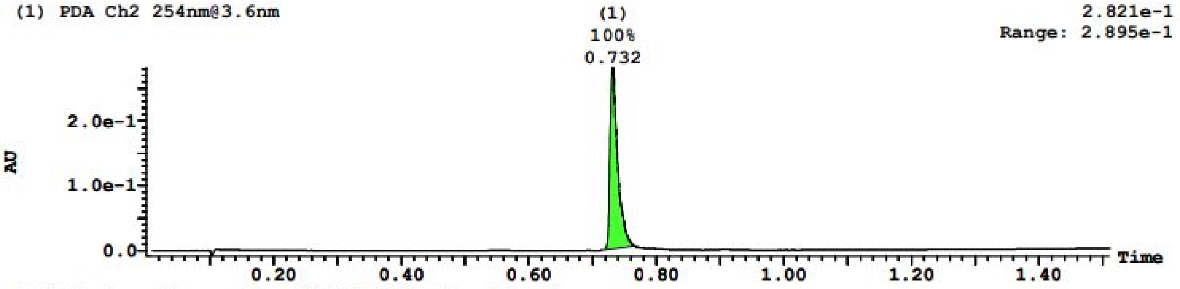

Peak Number	Time	Area %Total	BPM	Mass Found
1	0.732	100.00	368	367.0000

Compound 9h

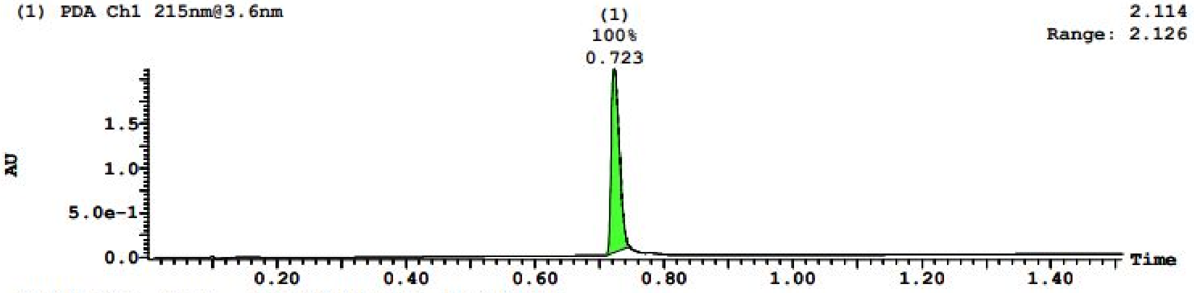

Peak Number	Time	Area %Total	BPM	Mass Found
1	0.723	100.00	368	367.0000

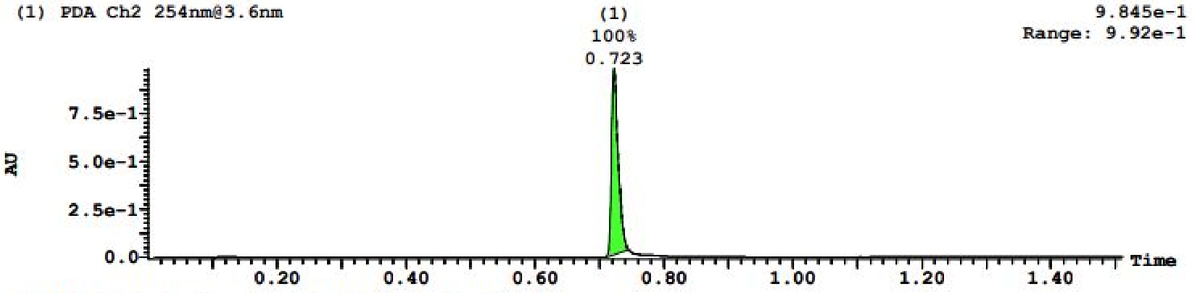

Peak Number	Time	Area %Total	BPM	Mass Found
1	0.723	100.00	368	367.0000

Compound 9i

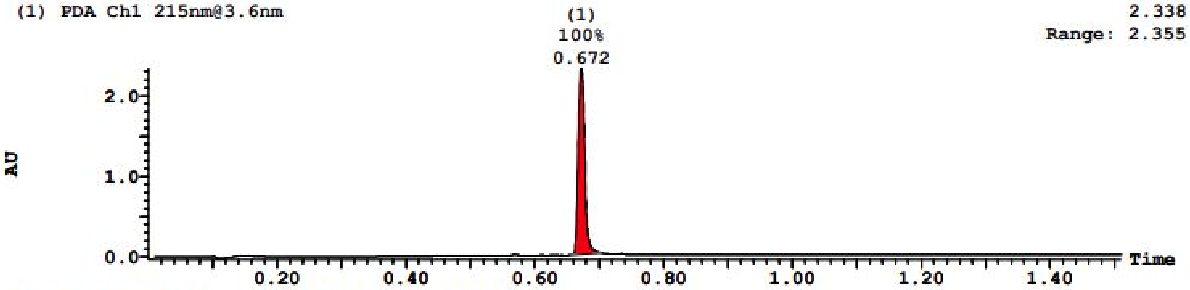

Peak Number	Time	Area %Total	BPM	Mass Found
1	0.672	100.00	369	Not Found

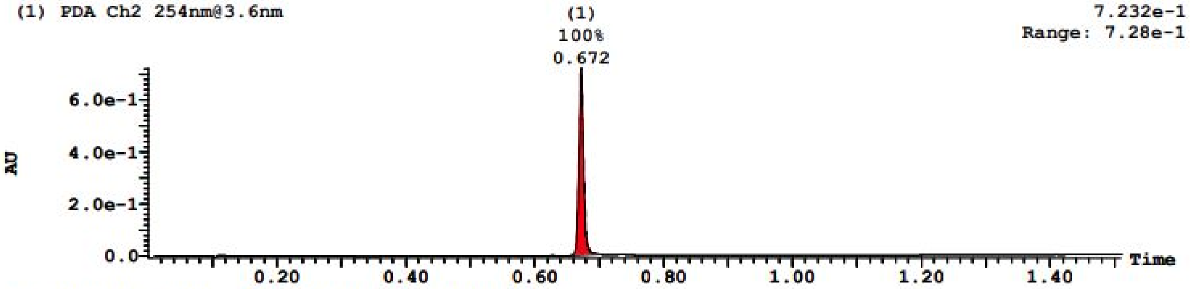

Peak Number	Time	Area %Total	BPM	Mass Found
1	0.672	100.00	369	Not Found

Compound 9j

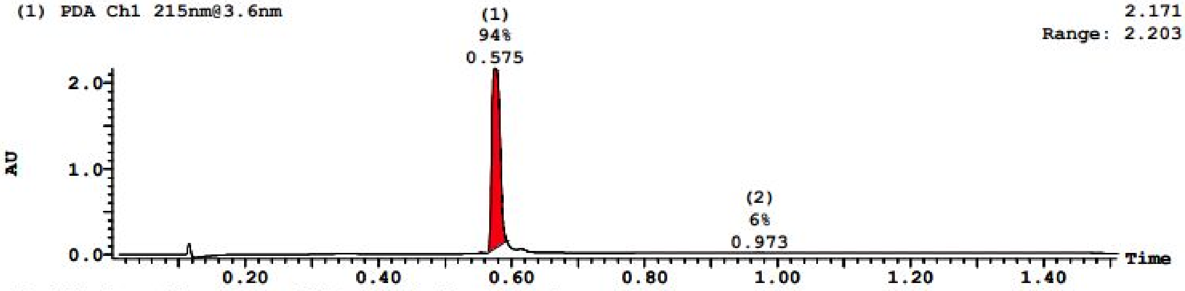

Peak Number	Time	Area %Total	BPM	Mass Found
1	0.575	94.48	366	Not Found
2	0.973	5.52	251	Not Found

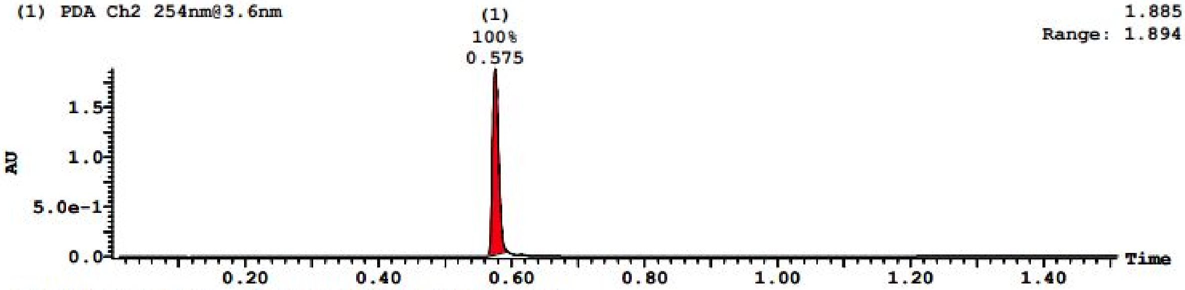

Peak Number	Time	Area %Total	BPM	Mass Found
1	0.575	100.00	366	Not Found

Compound 9k

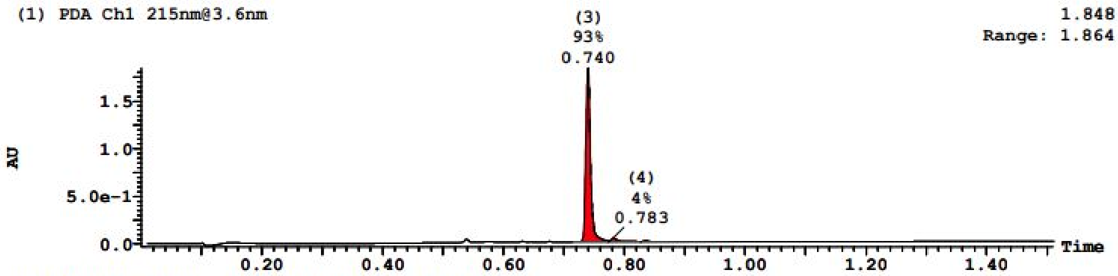

Peak Number	Time	Area %Total	BPM	Mass Found
3	0.740	92.89	352	Not Found
4	0.783	3.87	406	Not Found
5	0.836	3.24	392	Not Found

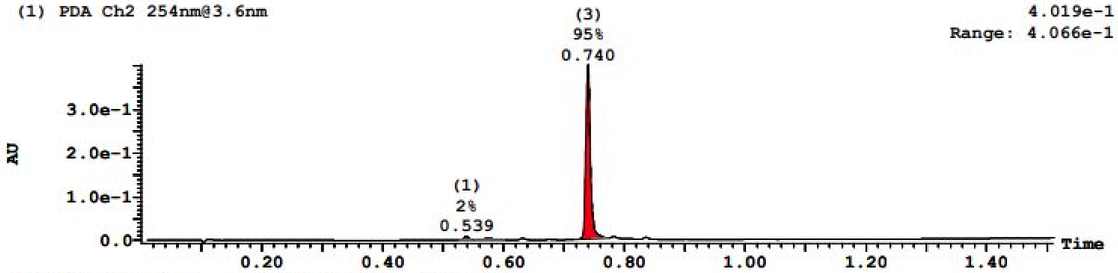

Peak Number	Time	Area %Total	BPM	Mass Found
1	0.539	2.23	330	Not Found
2	0.572	2.50	368	Not Found
3	0.740	95.27	352	Not Found

Compound 15a

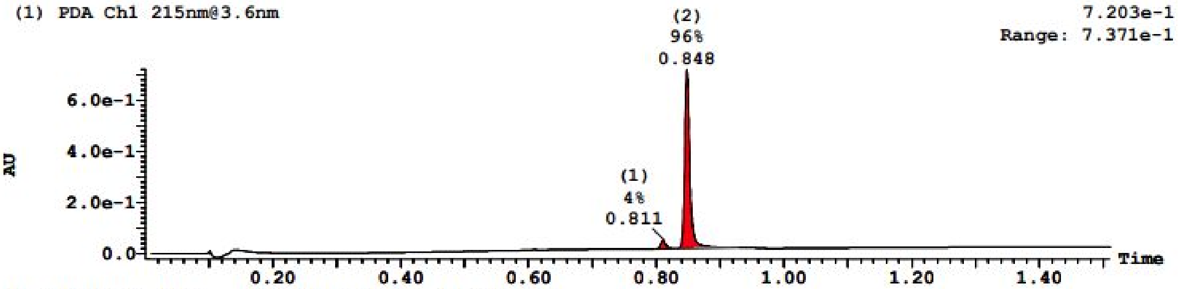

Peak Number	Time	Area %Total	BPM	Mass Found
1	0.811	4.32	346	Not Found
2	0.848	95.68	379	Not Found

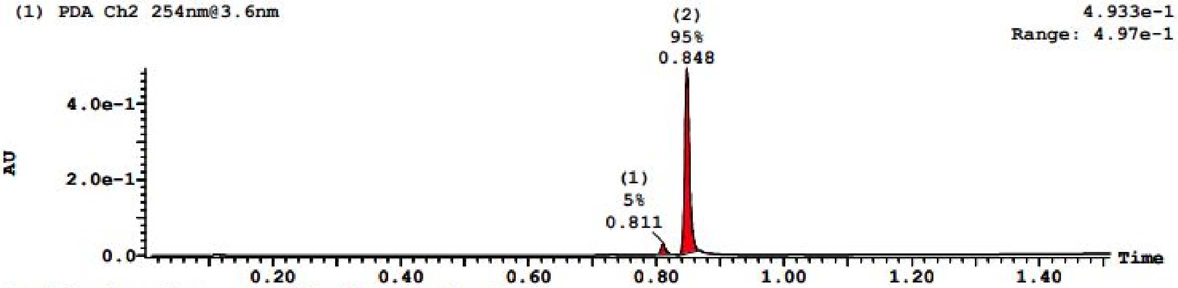

Peak Number	Time	Area %Total	BPM	Mass Found
1	0.811	5.49	346	Not Found
2	0.848	94.51	379	Not Found

Compound 15b

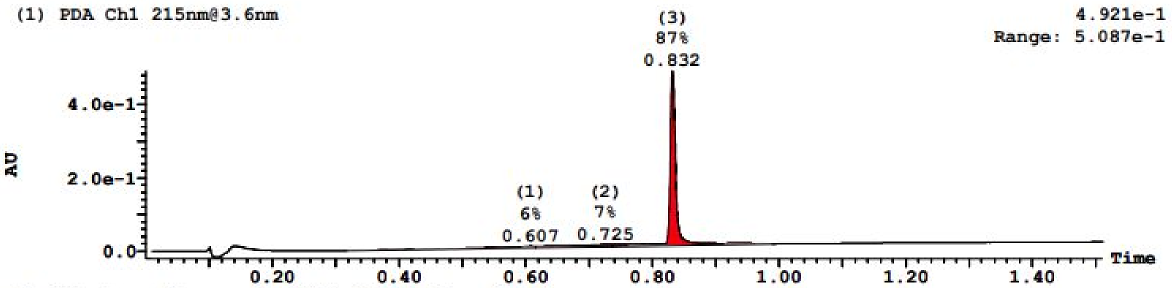

Peak Number	Time	Area %Total	BPM	Mass Found
1	0.607	5.93	345	Not Found
2	0.725	7.28	279	Not Found
3	0.832	86.79	380	Not Found

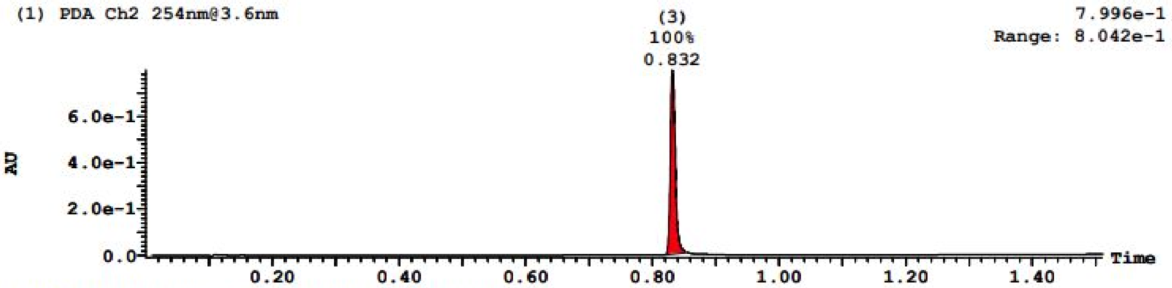

Peak Number	Time	Area %Total	BPM	Mass Found
3	0.832	100.00	380	Not Found

Compound 15c

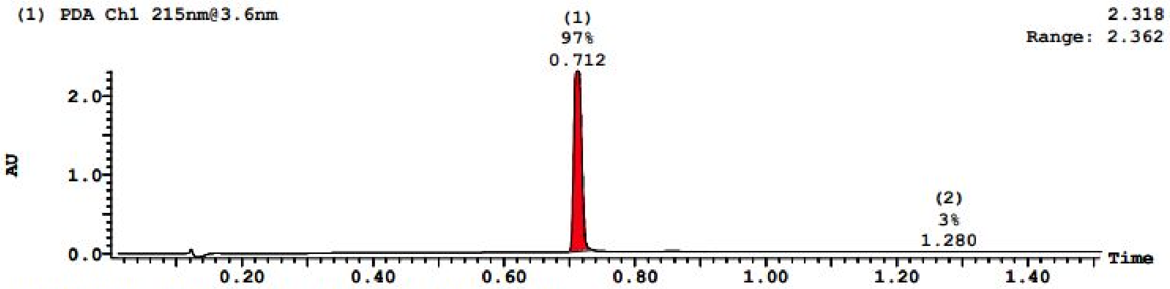

Peak Number	Time	Area %Total	BPM	Mass Found
1	0.712	97.01	344	Not Found
2	1.280	2.99	279	Not Found

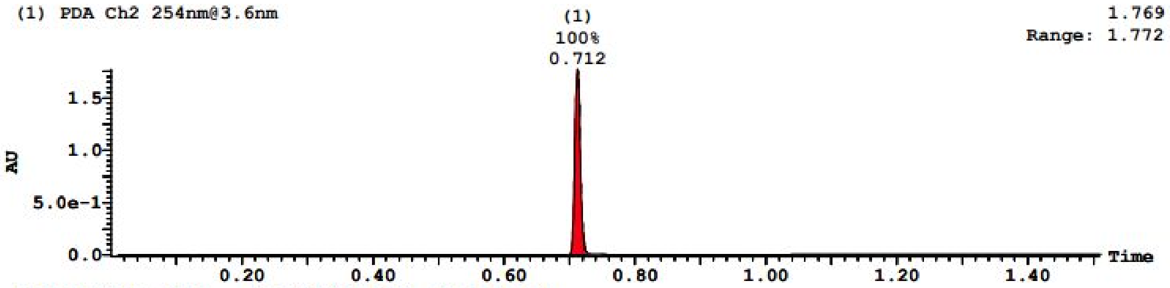

Peak Number	Time	Area %Total	BPM	Mass Found
1	0.712	100.00	344	Not Found

Compound 15d

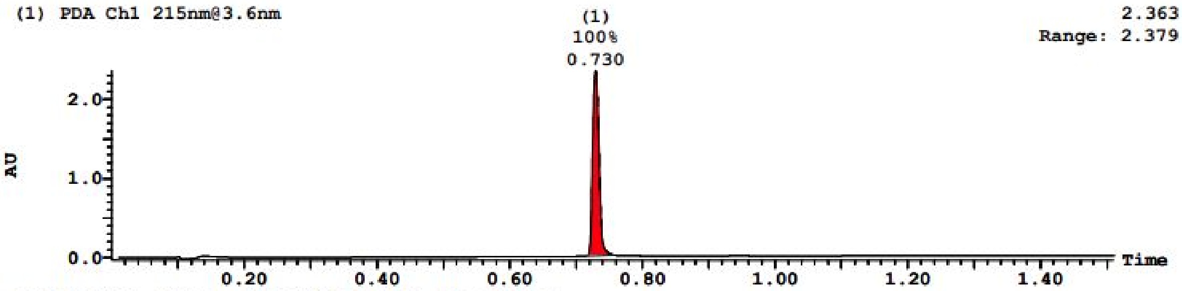

Peak Number	Time	Area %Total	BPM	Mass Found
1	0.730	100.00	344	Not Found

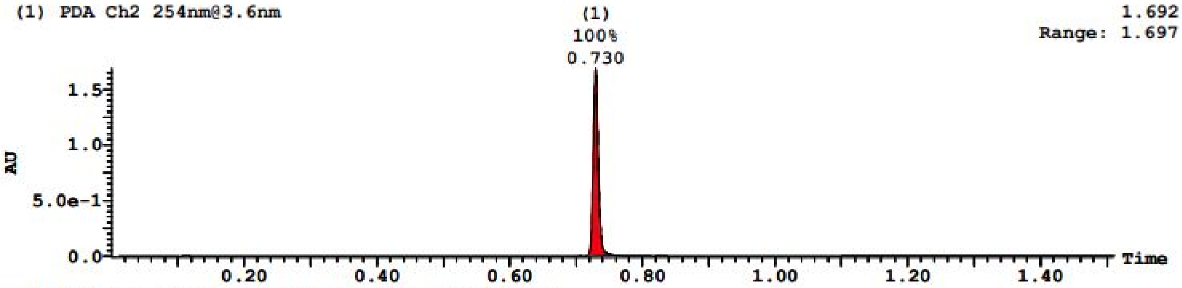

Peak Number	Time	Area %Total	BPM	Mass Found
1	0.730	100.00	344	Not Found

Compound 15e

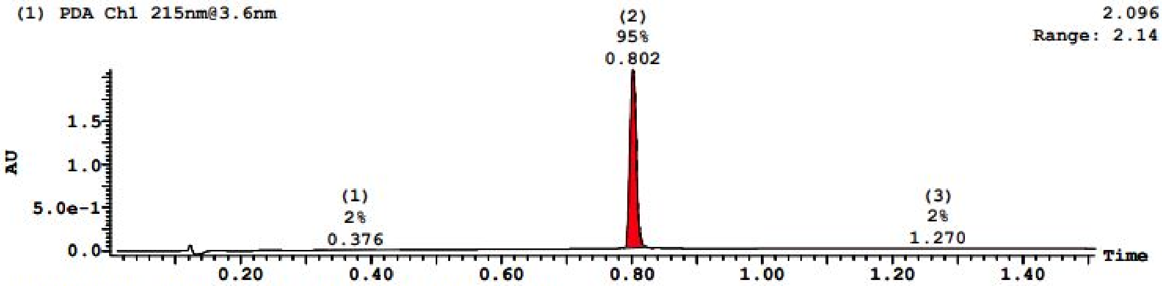

Peak Number	Time	Area %Total	BPM	Mass Found
1	0.376	2.31	192	Not Found
2	0.802	95.42	401	Not Found
3	1.270	2.27	251	Not Found

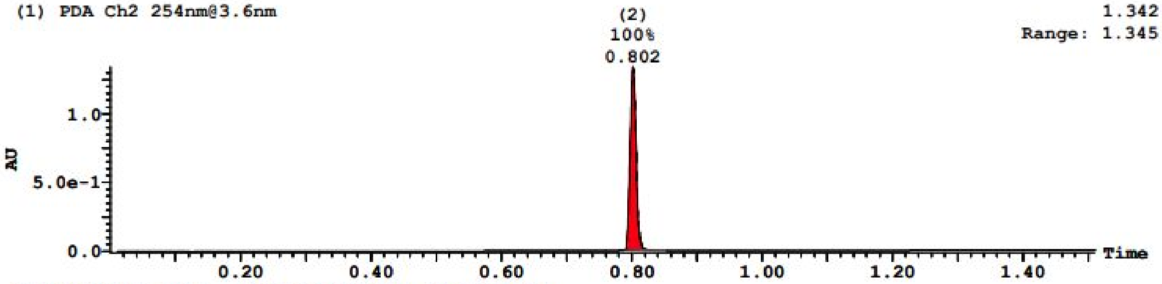

Peak Number	Time	Area %Total	BPM	Mass Found
2	0.802	100.00	401	Not Found

Compound 15f

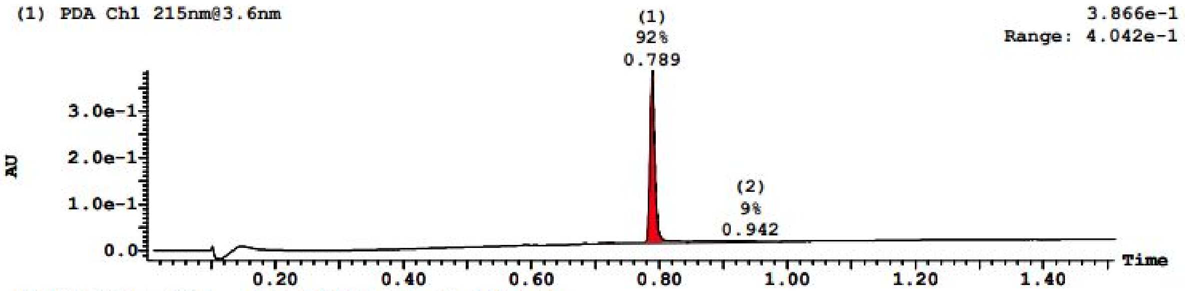

Peak Number	Time	Area %Total	BPM	Mass Found
1	0.789	91.50	384	Not Found
2	0.942	8.50	399	Not Found

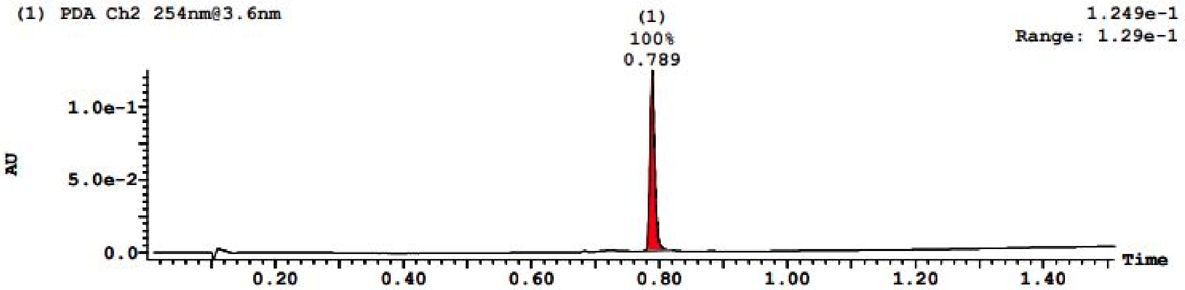

Peak Number	Time	Area %Total	BPM	Mass Found
1	0.789	100.00	384	Not Found

Compound 19a

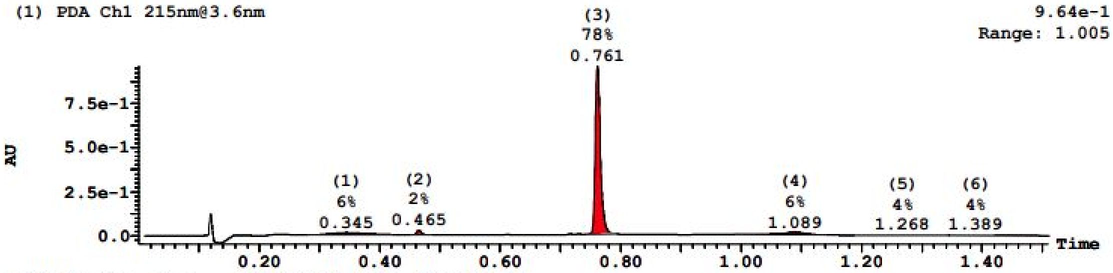

Peak Number	Time	Area %Total	BPM	Mass Found
1	0.345	6.16	159	Not Found
2	0.465	1.83	292	Not Found
3	0.761	78.22	398	Not Found
4	1.089	6.39	159	Not Found
5	1.268	3.76	227	Not Found
6	1.389	3.64	235	Not Found

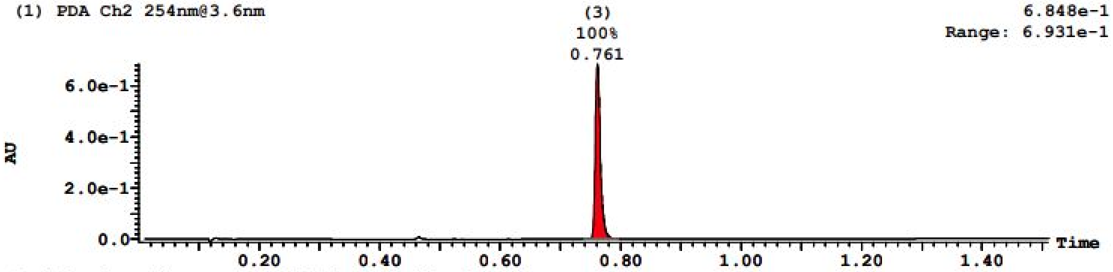

Peak Number	Time	Area %Total	BPM	Mass Found
3	0.761	100.00	398	Not Found

Compound 19b

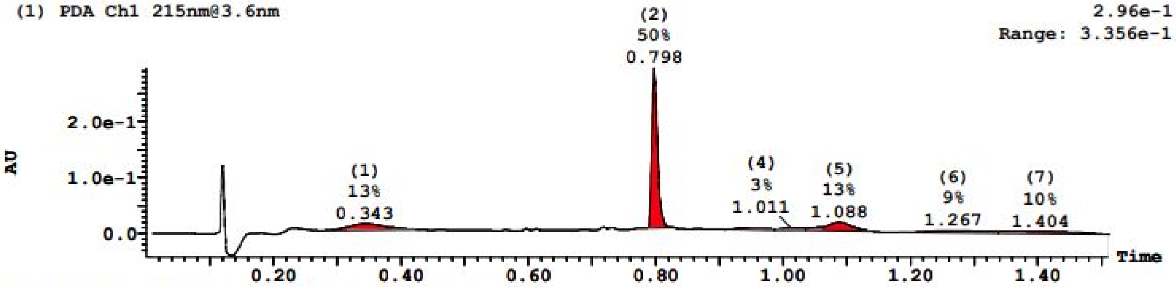

Peak Number	Time	Area %Total	BPM	Mass Found
1	0.343	12.61	279	Not Found
2	0.798	50.42	410	Not Found
3	0.933	2.18	189	Not Found
4	1.011	2.87	147	Not Found
5	1.088	12.99	227	Not Found
6	1.267	8.59	159	Not Found
7	1.404	10.34	227	Not Found

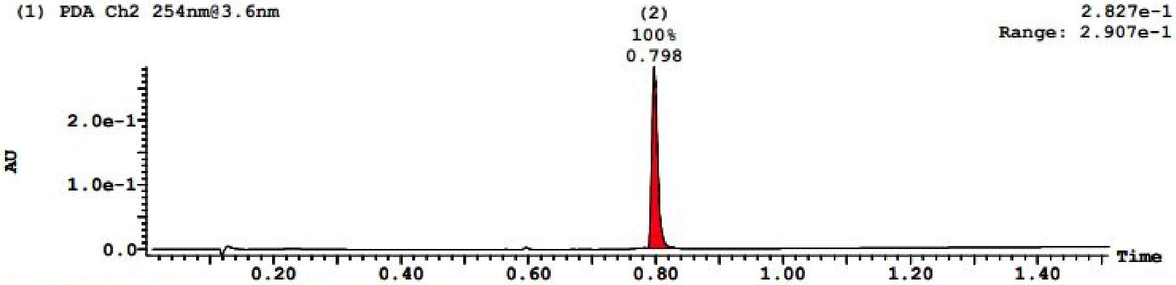

Peak Number	Time	Area %Total	BPM	Mass Found
2	0.798	100.00	410	Not Found

Compound 19c

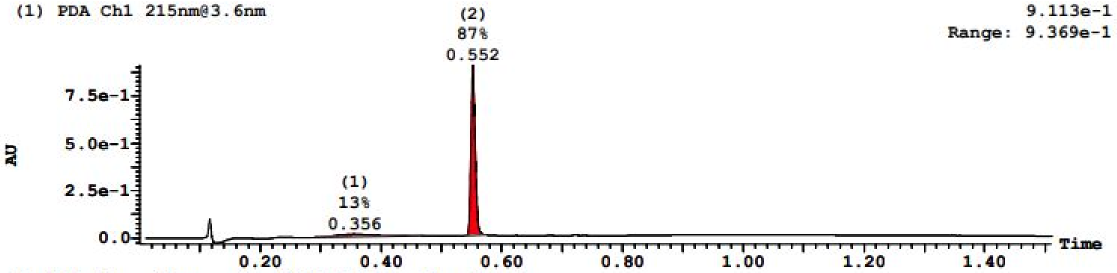

Peak Number	Time	Area %Total	BPM	Mass Found
1	0.356	12.61	145	Not Found
2	0.552	87.39	393	Not Found

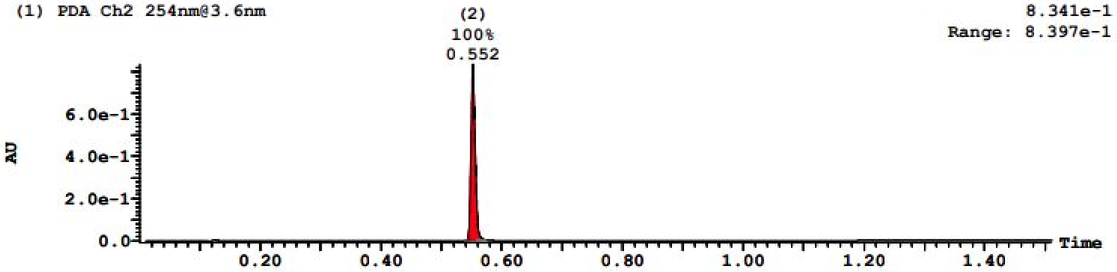

Peak Number	Time	Area %Total	BPM	Mass Found
2	0.552	100.00	393	Not Found

Compound 19d

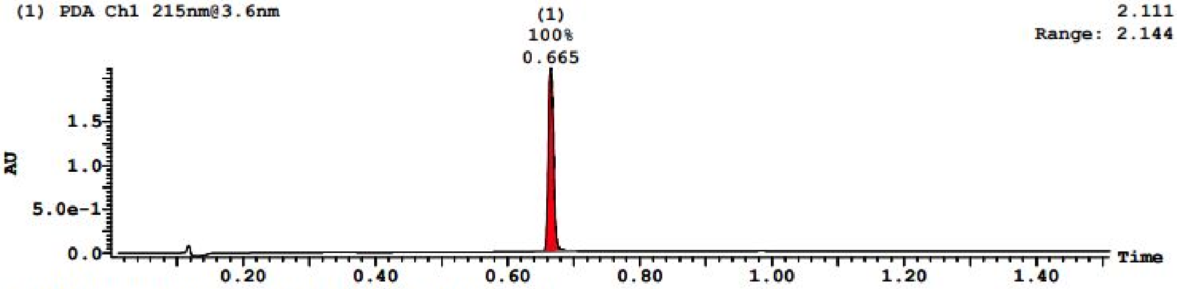

Peak Number	Time	Area %Total	BPM	Mass Found
1	0.665	100.00	396	Not Found

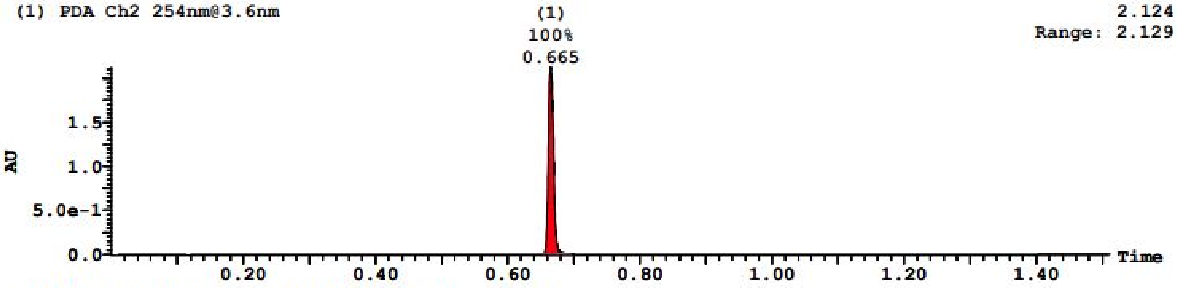

Peak Number	Time	Area %Total	BPM	Mass Found
1	0.665	100.00	396	Not Found

^{1}H NMR Spectra for Compound 6

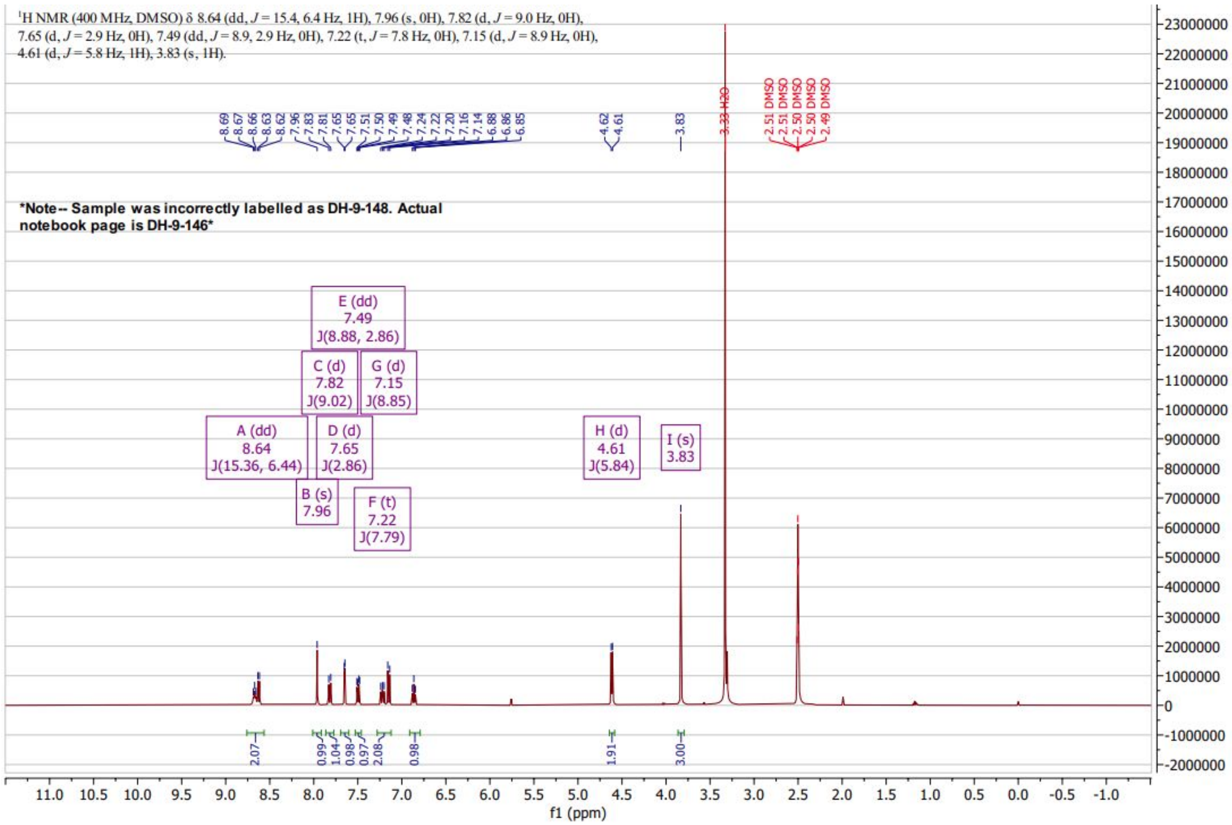

^{13}C NMR Spectra for Compound 6

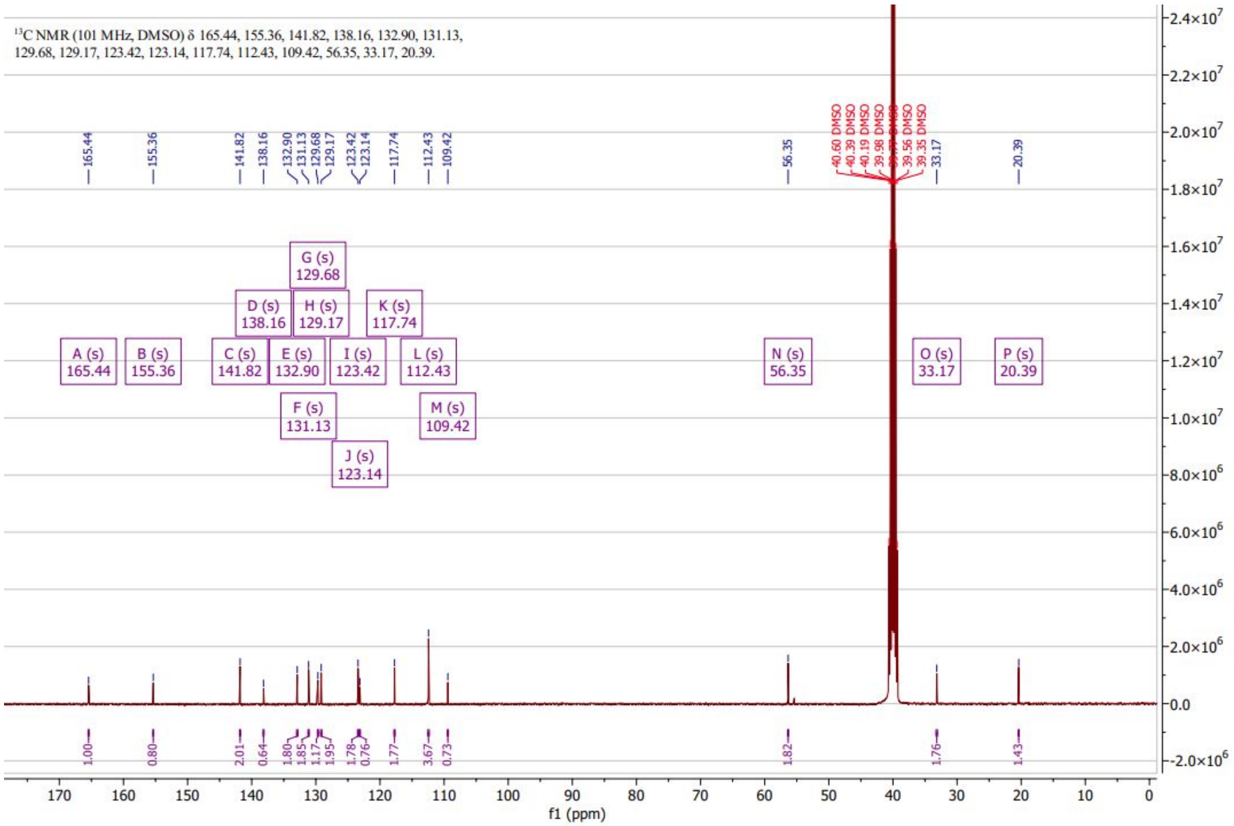

^{1}H NMR Spectra for Compound 9a

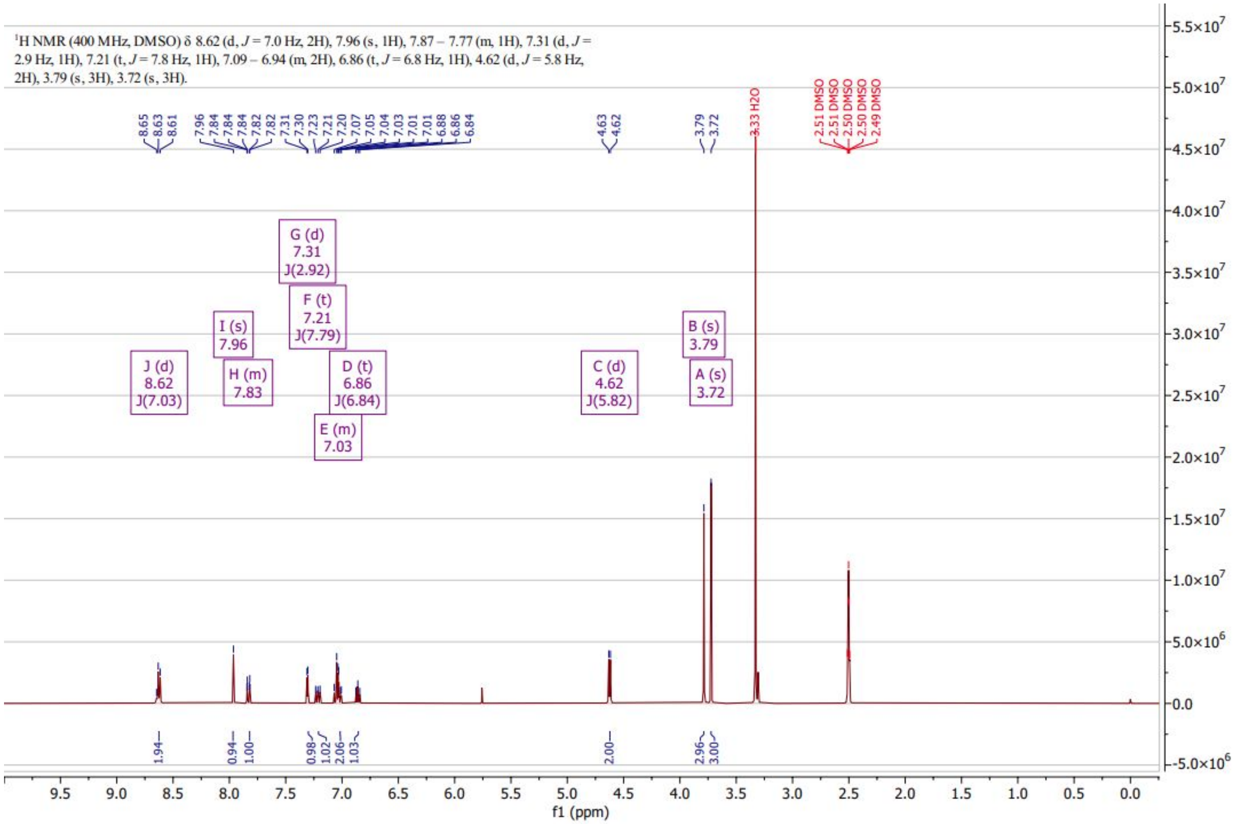

^{13}C NMR Spectra for Compound 9a

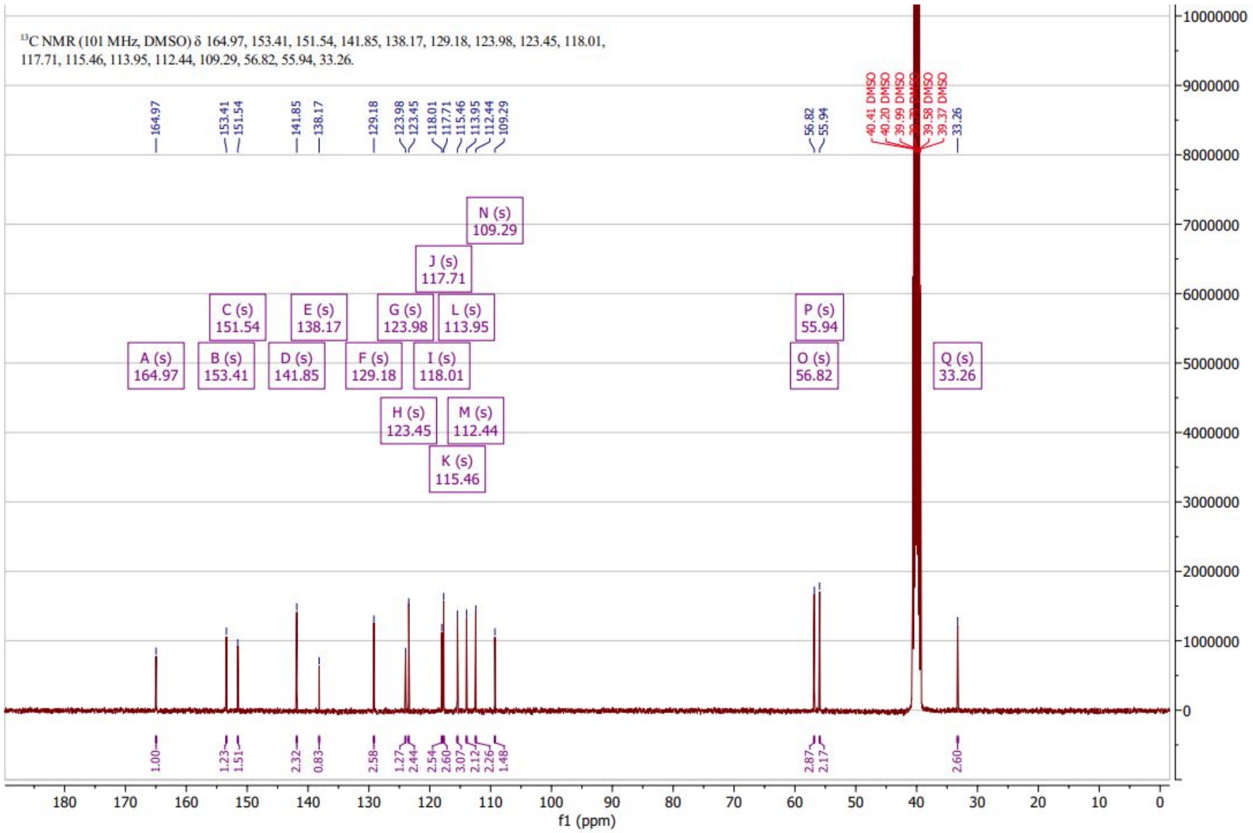

^{1}H NMR Spectra for Compound 9b

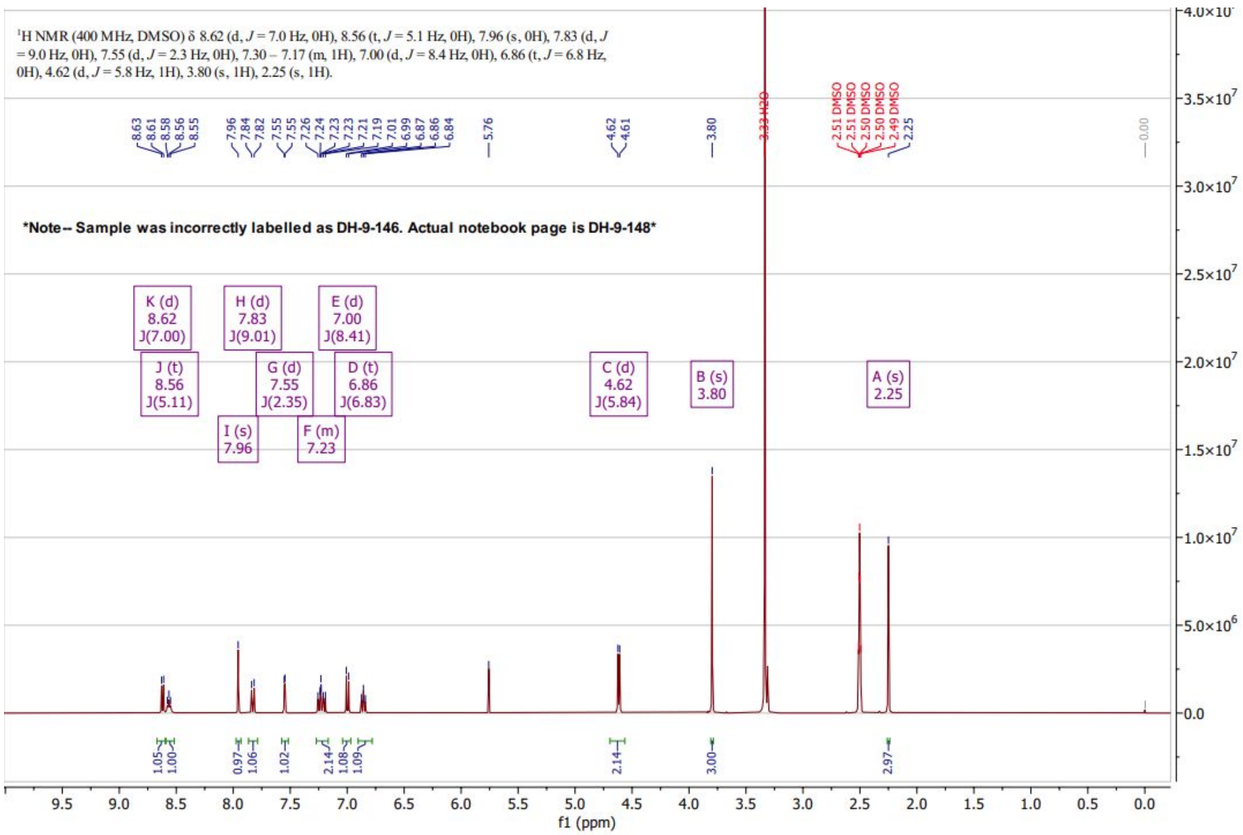

^{13}C NMR Spectra for Compound 9b

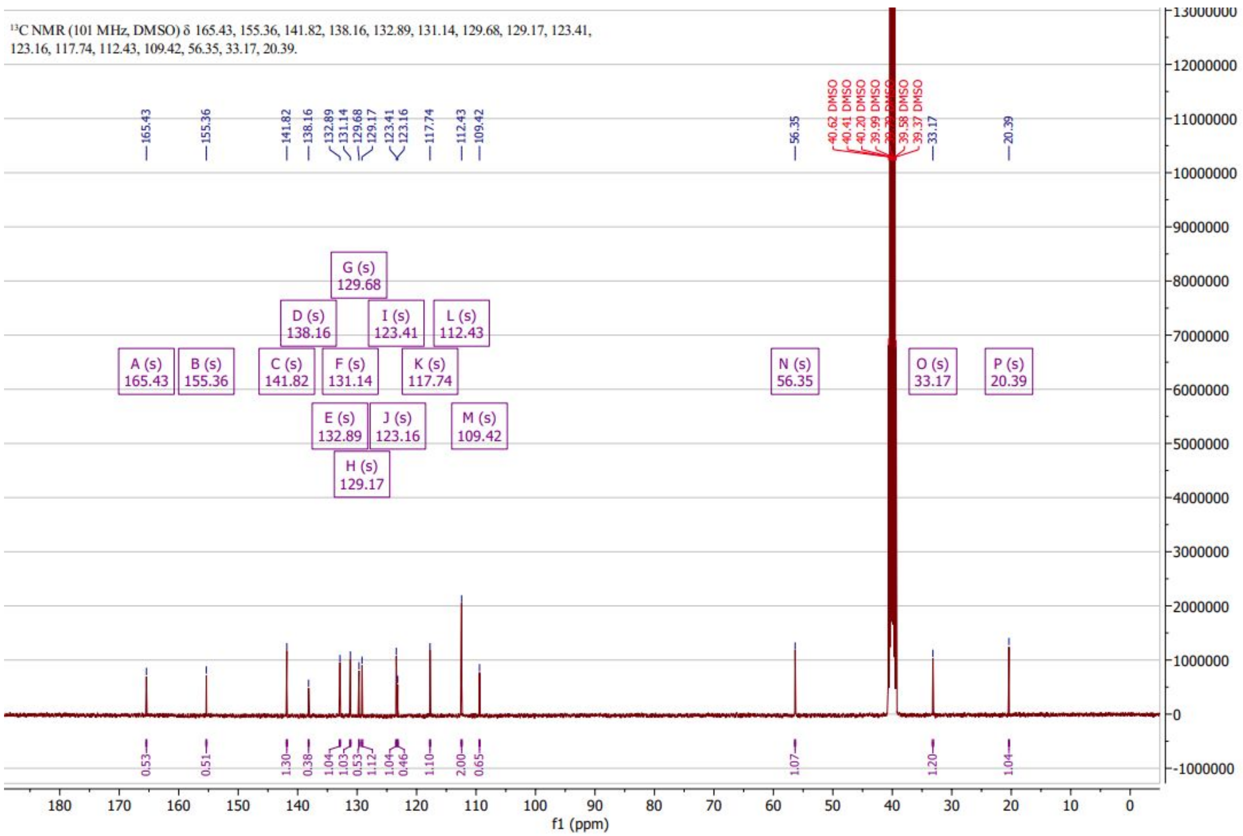

^{1}H NMR Spectra for Compound 9c

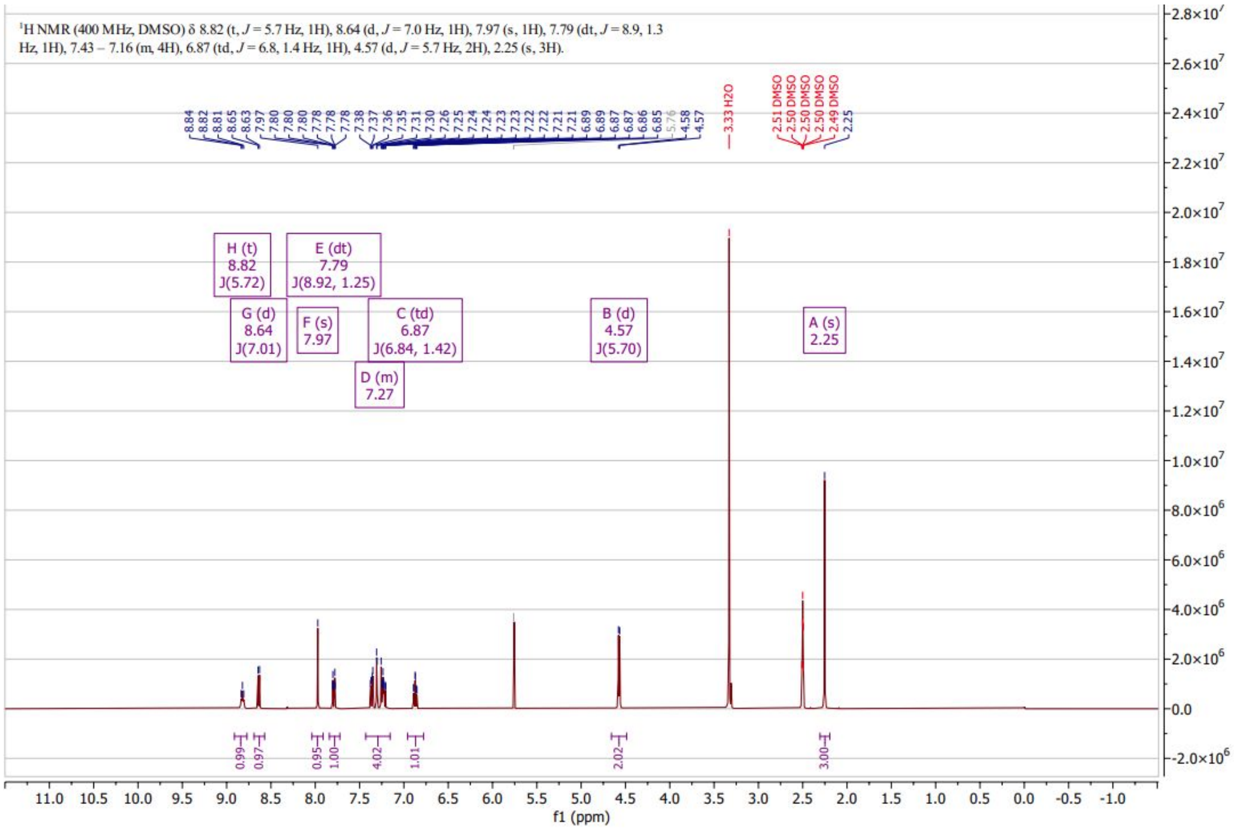

^{13}C NMR Spectra for Compound 9c

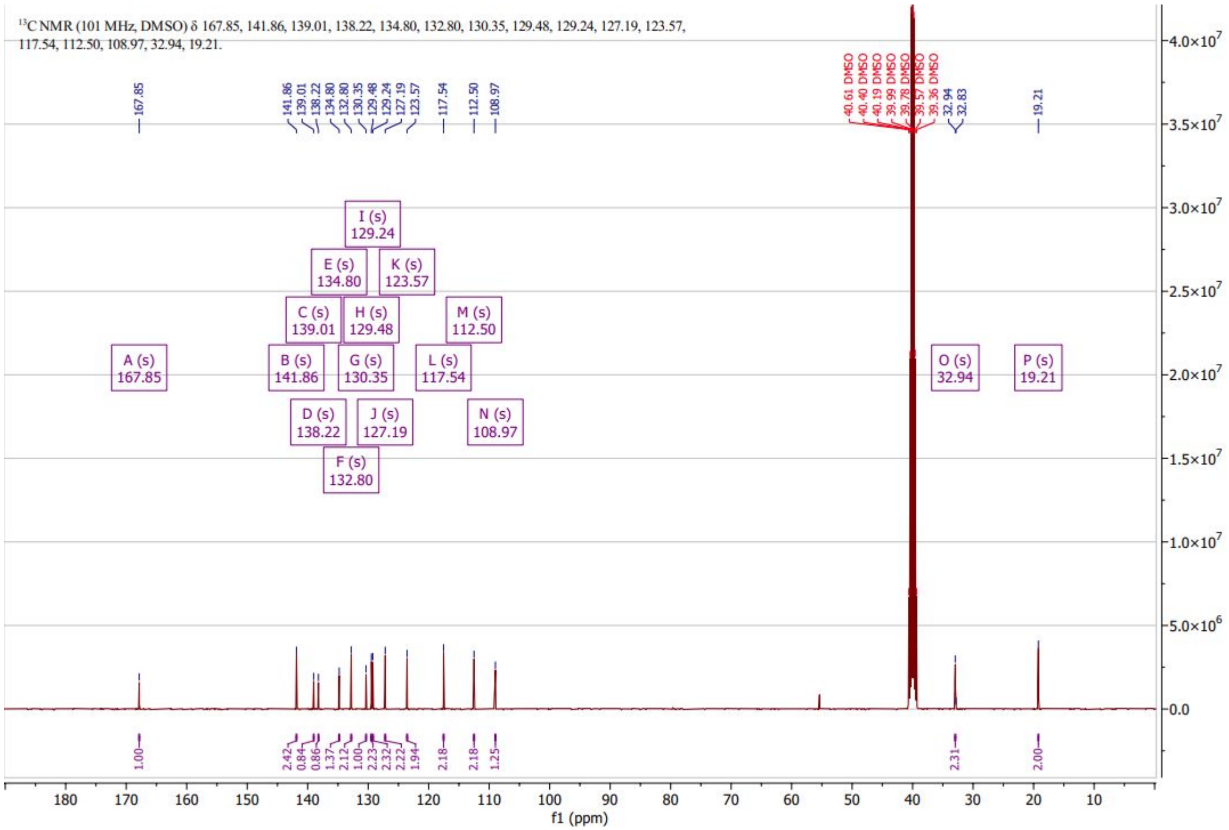

¹H NMR Spectra for Compound 9d

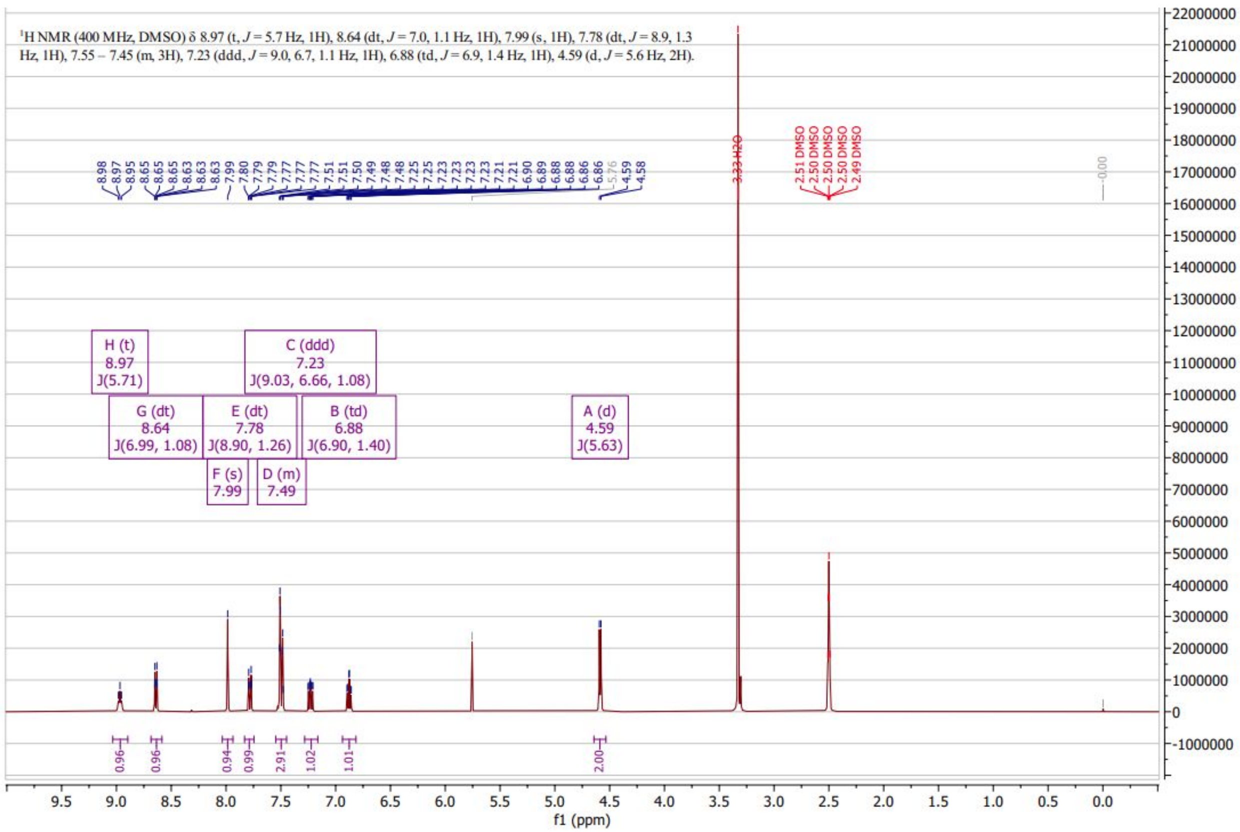

^{13}C NMR Spectra for Compound 9d

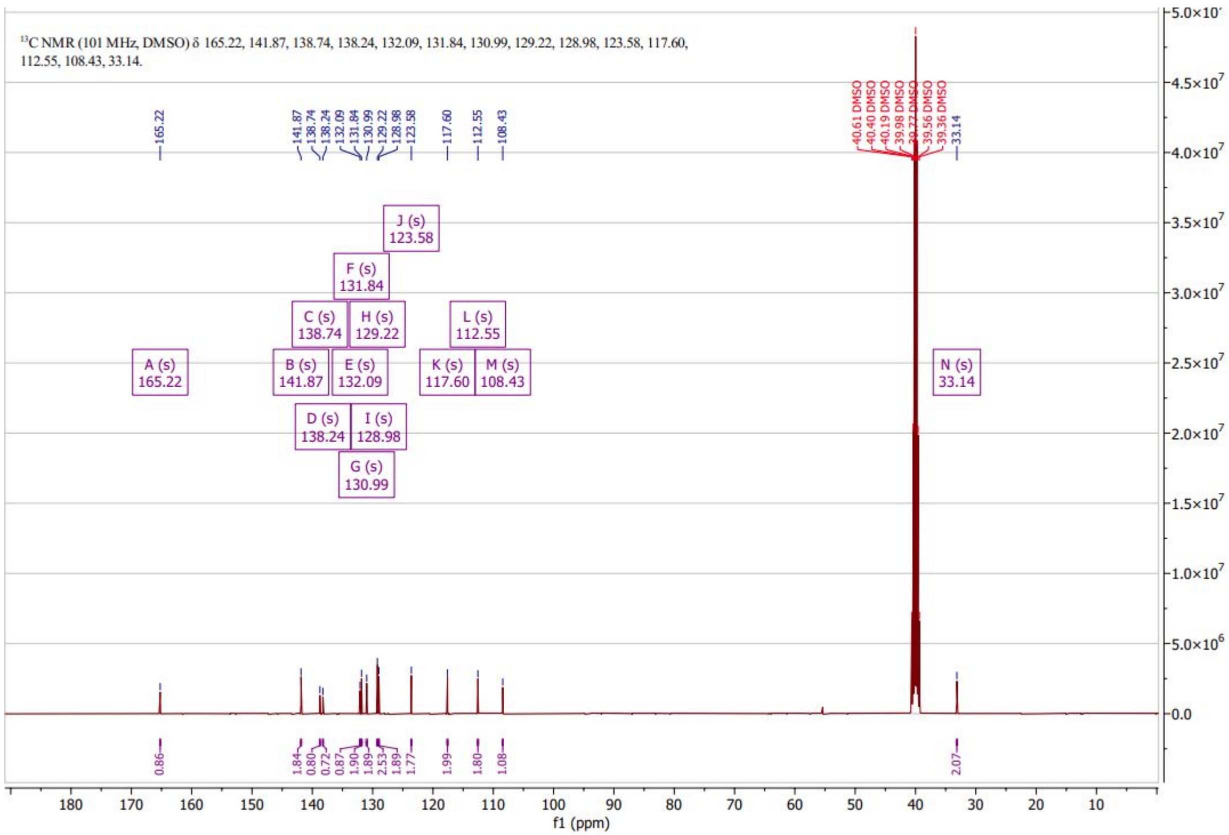

^{1}H NMR Spectra for Compound 9f

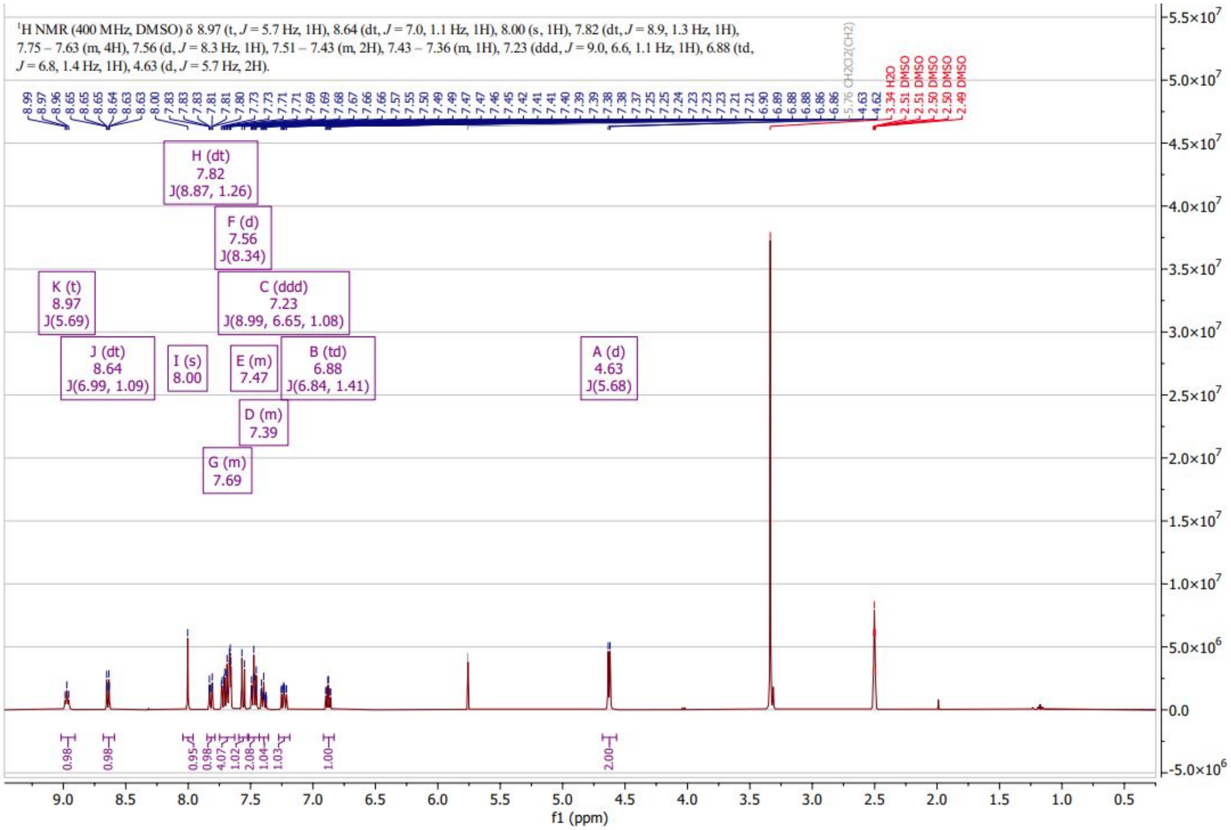

^{13}C NMR Spectra for Compound 9f

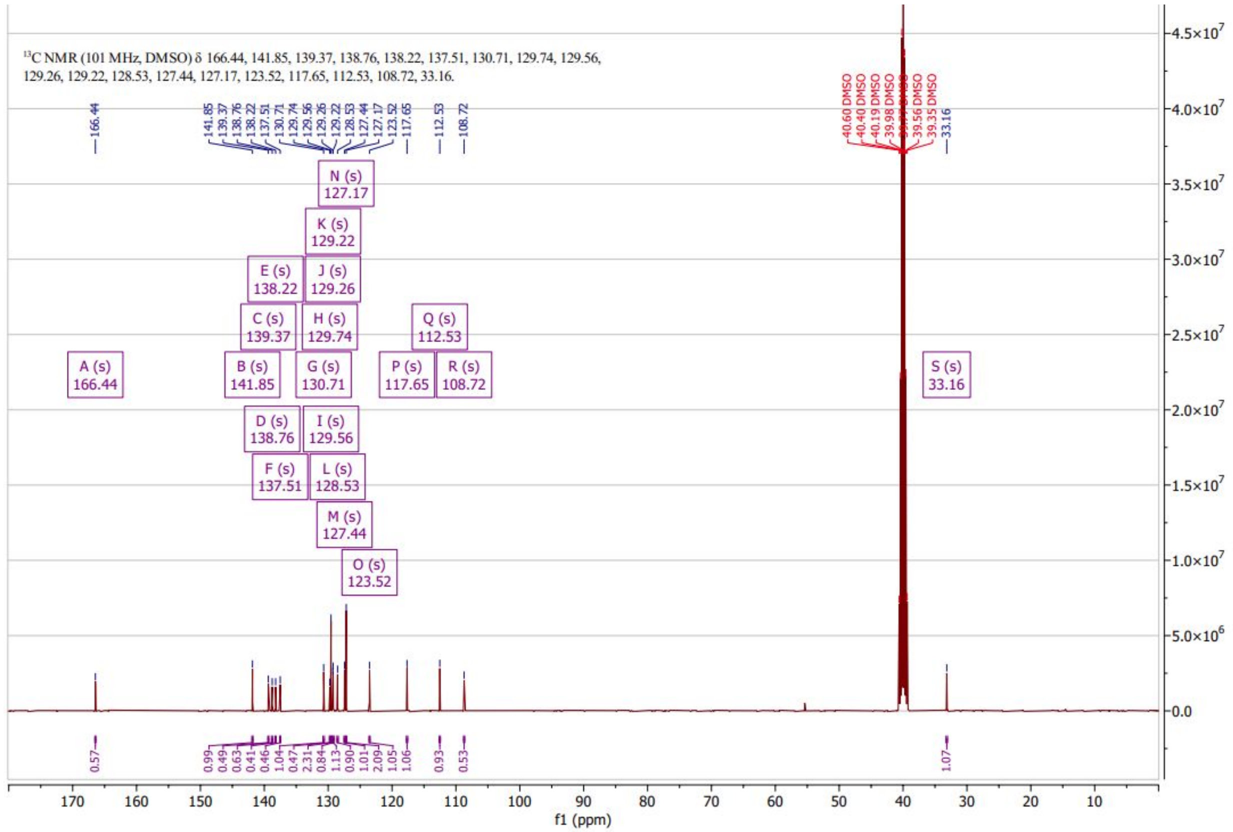

^{1}H NMR Spectra for Compound 9g

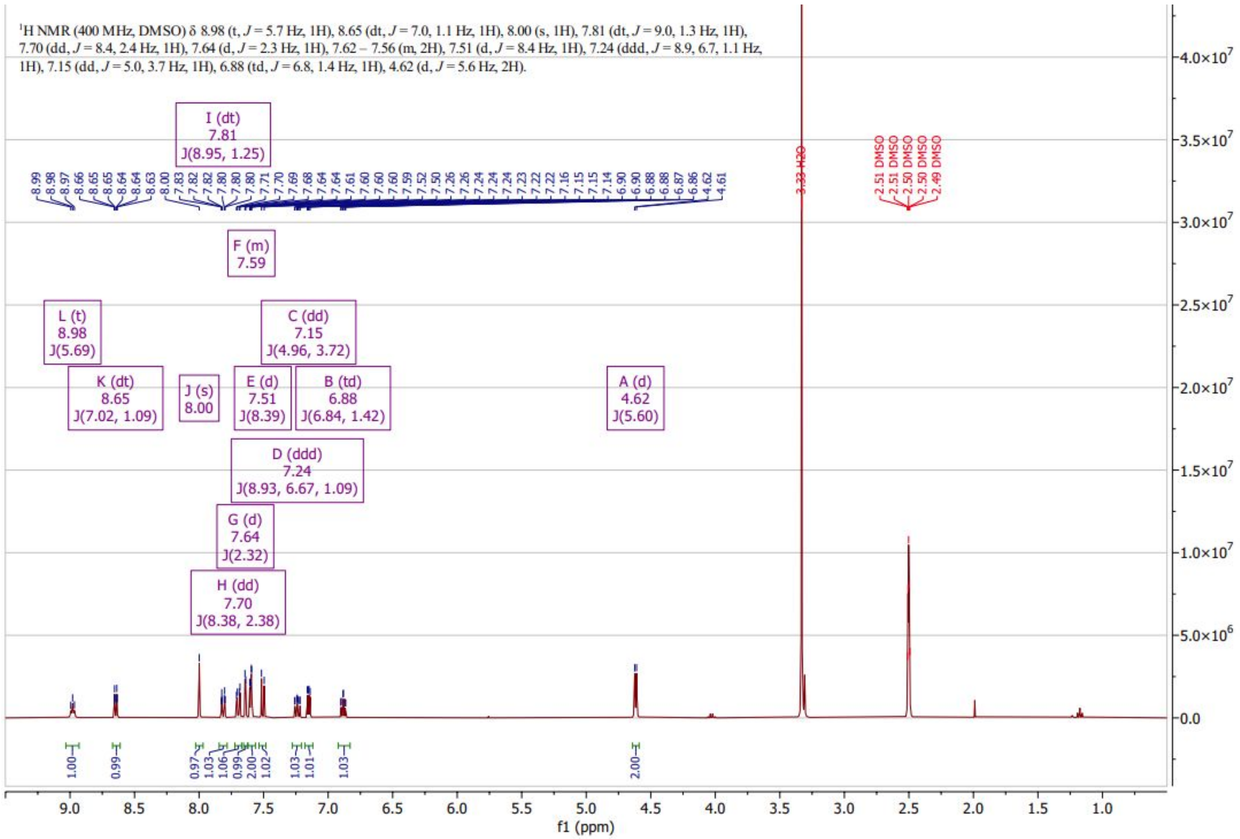

^{13}C NMR Spectra for Compound 9g

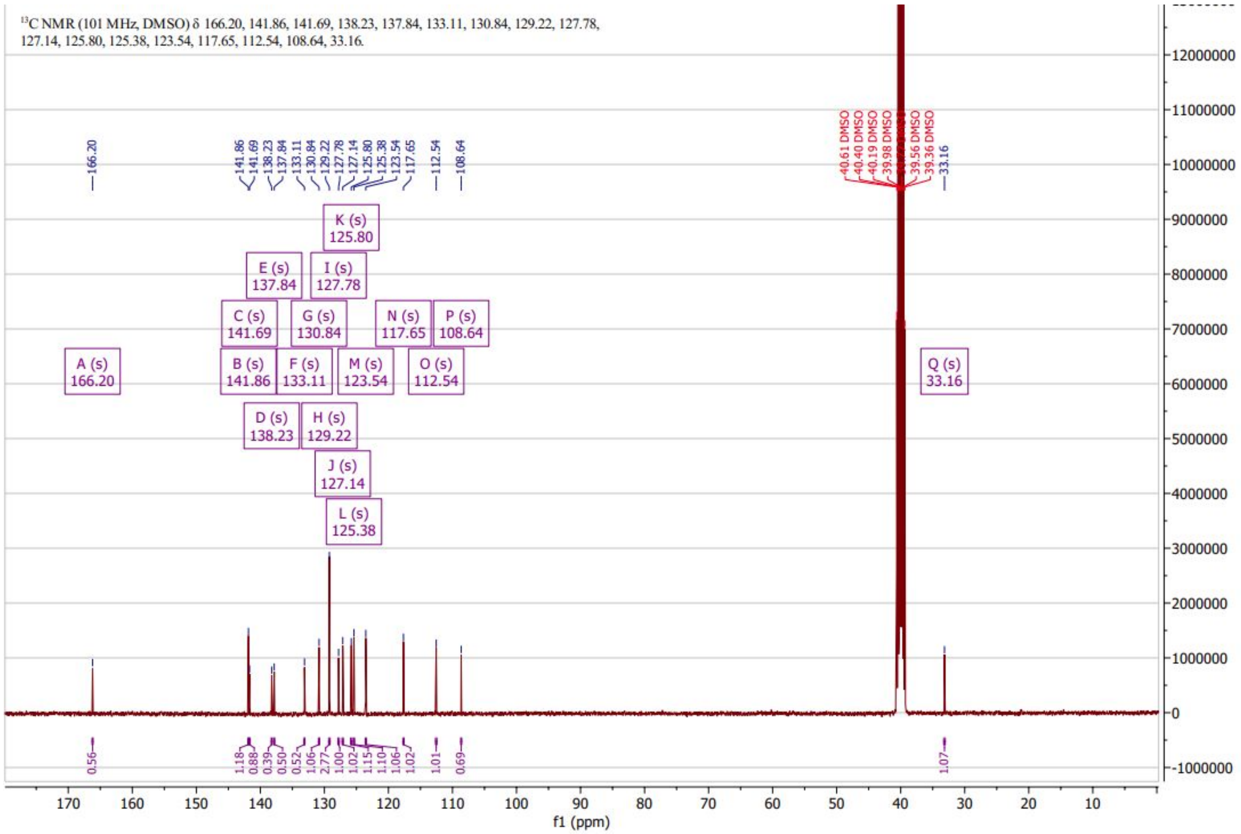

^{1}H NMR Spectra for Compound 9h

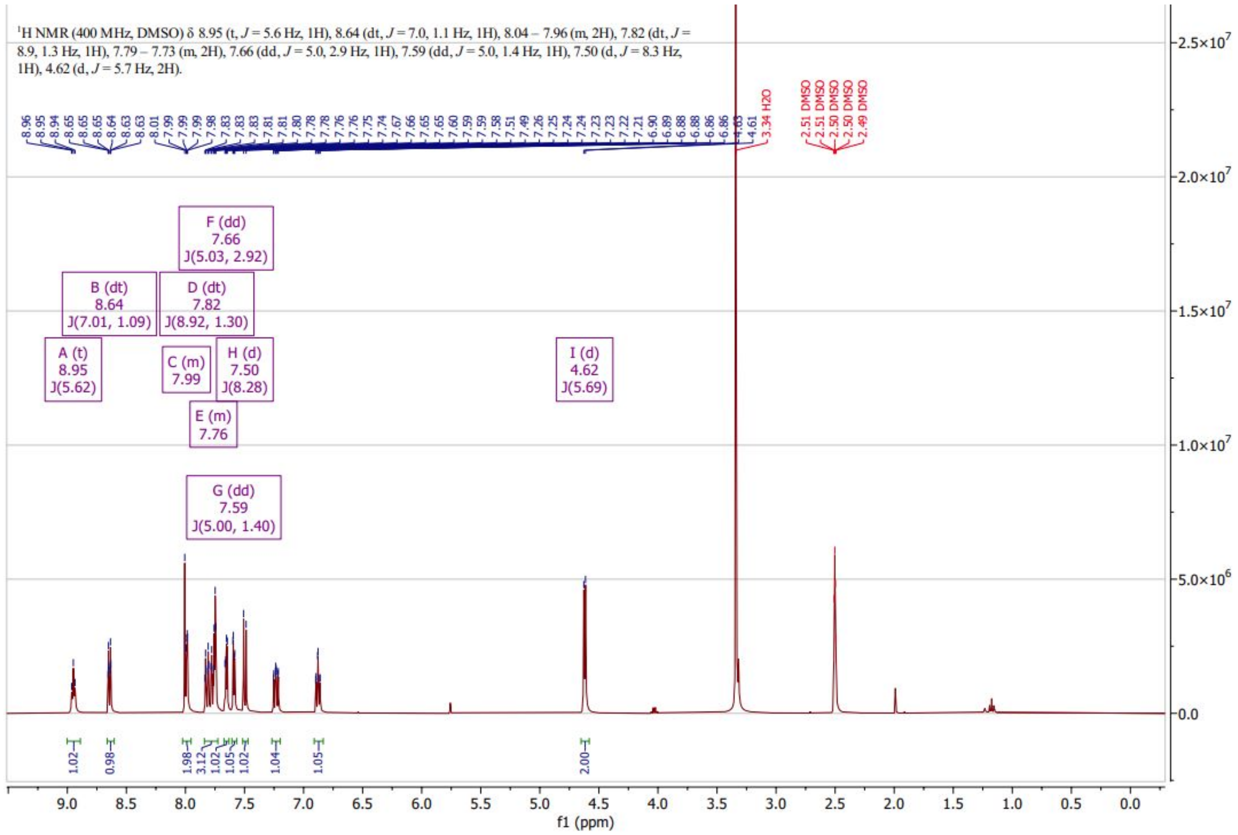

^{13}C NMR Spectra for Compound 9h

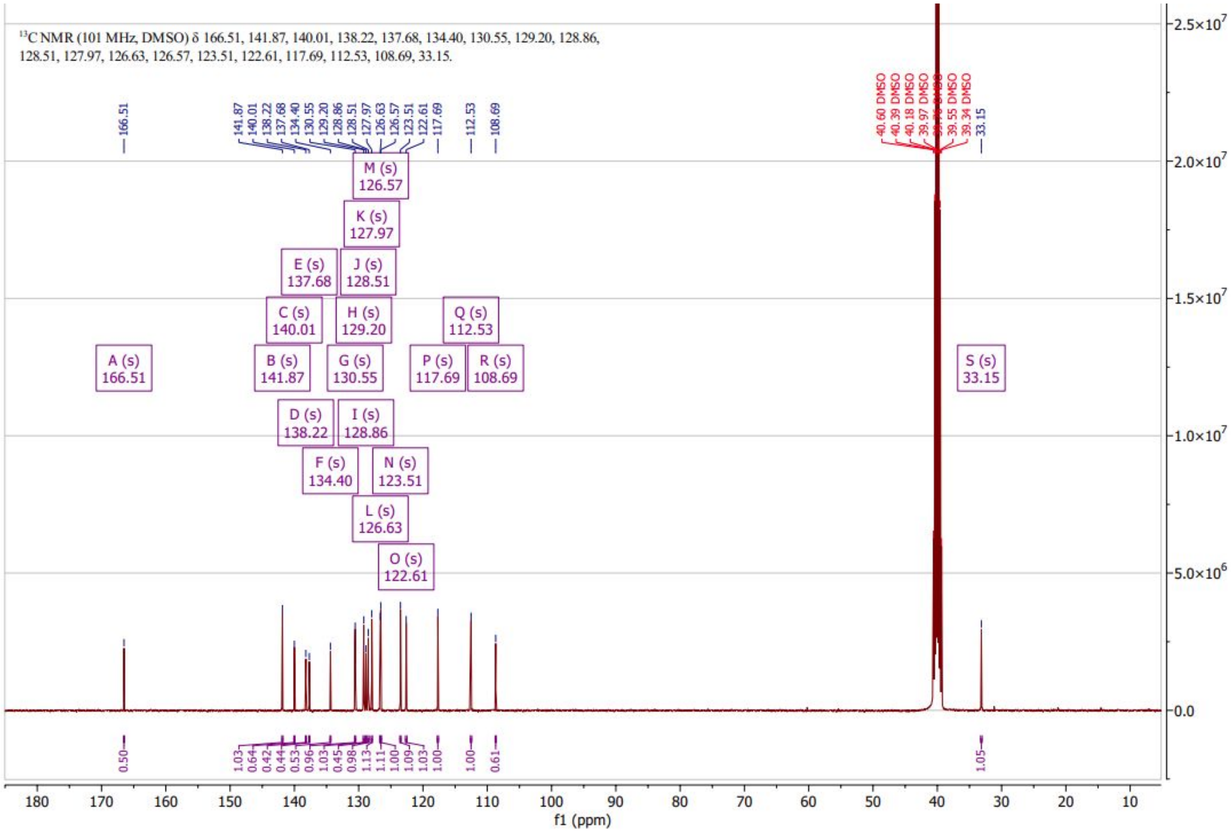

^{1}H NMR Spectra for Compound 9i

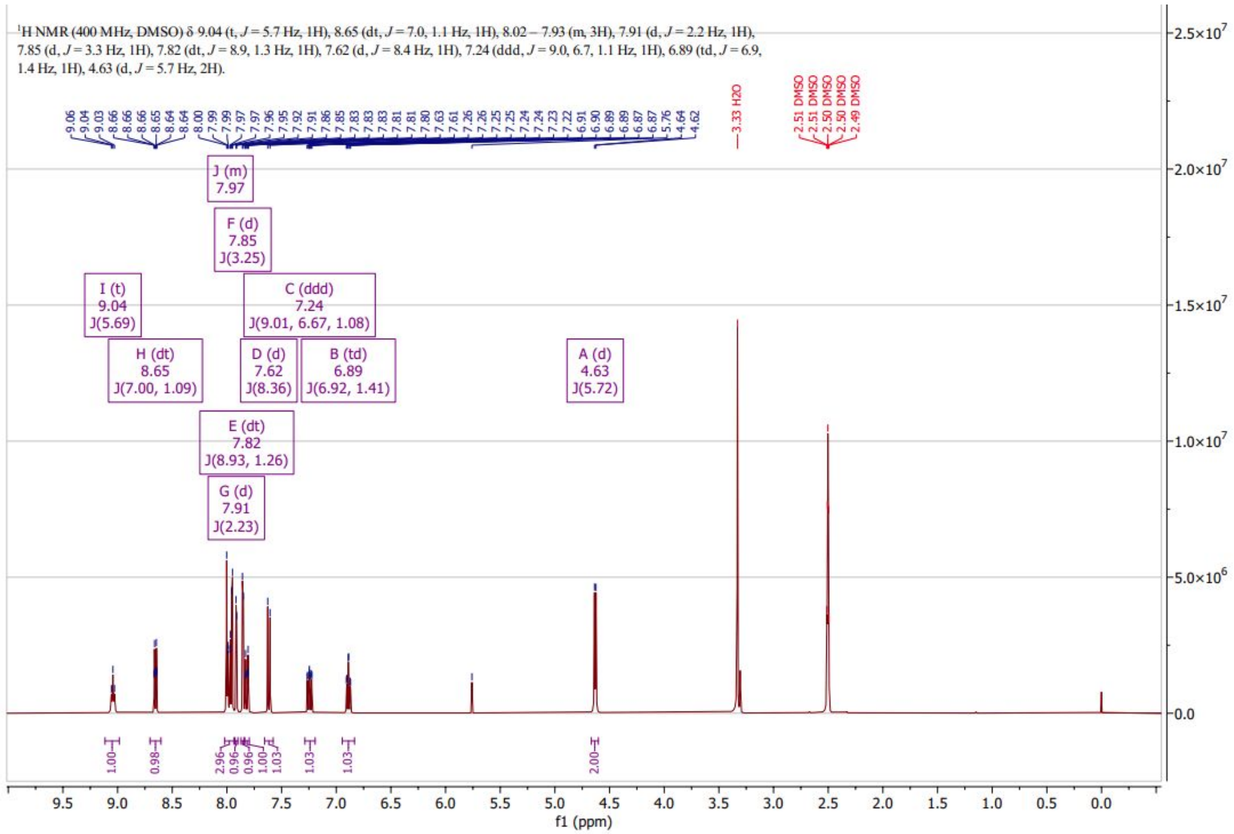

^{13}C NMR Spectra for Compound 9i

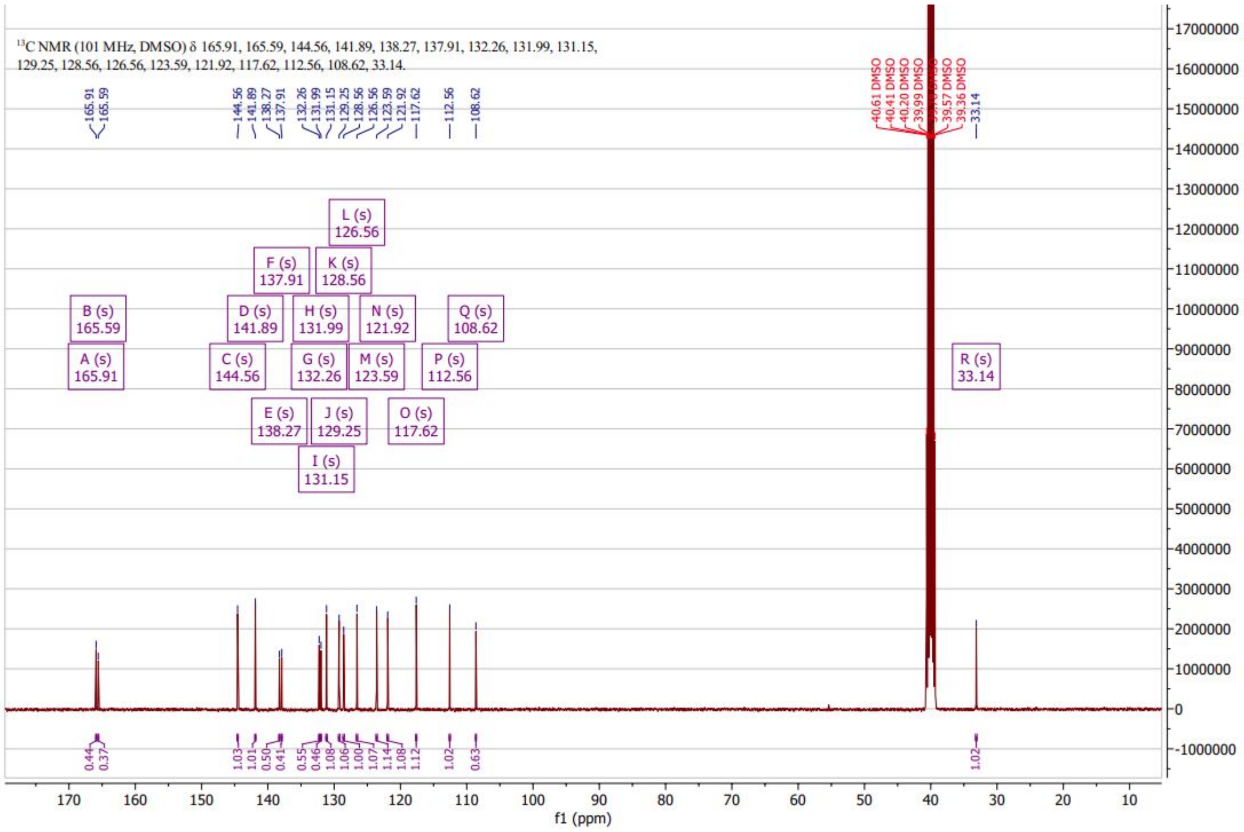

^{1}H NMR Spectra for Compound 9j

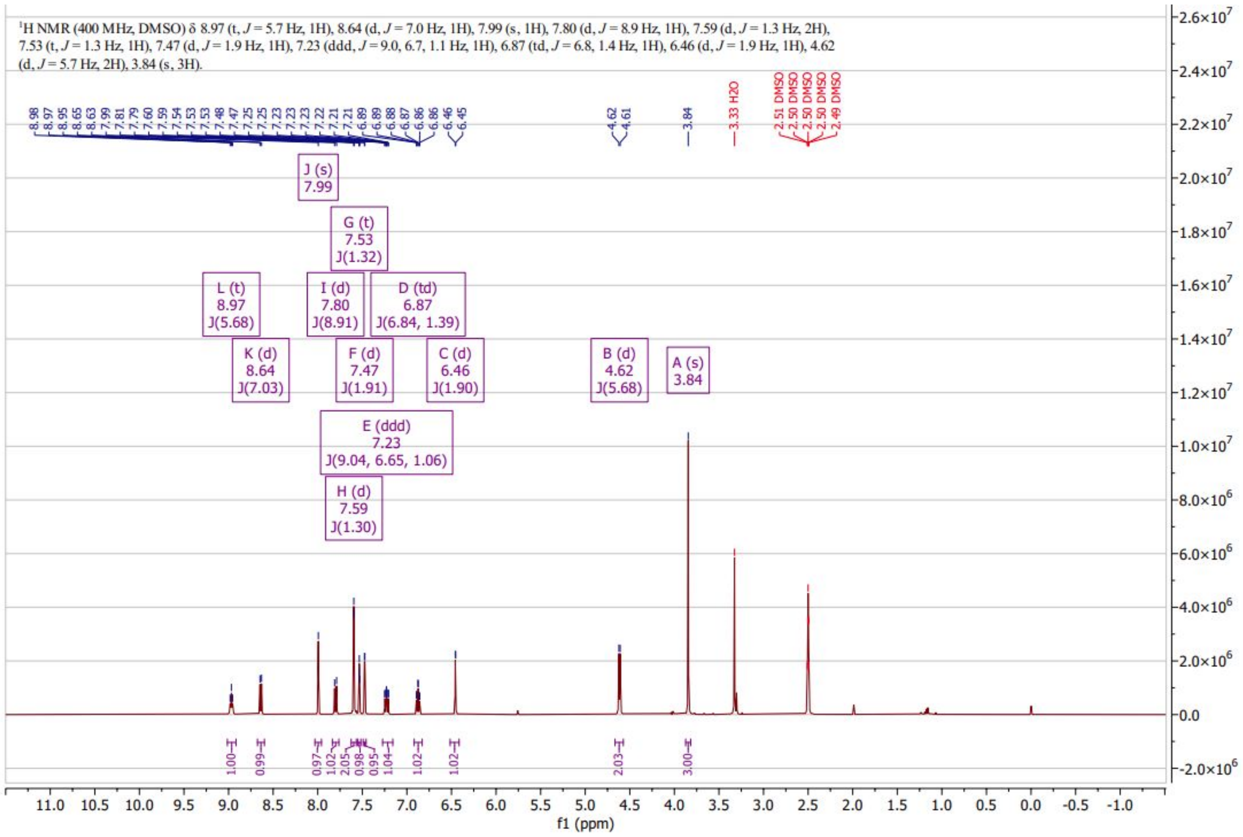

^{13}C NMR Spectra for Compound 9j

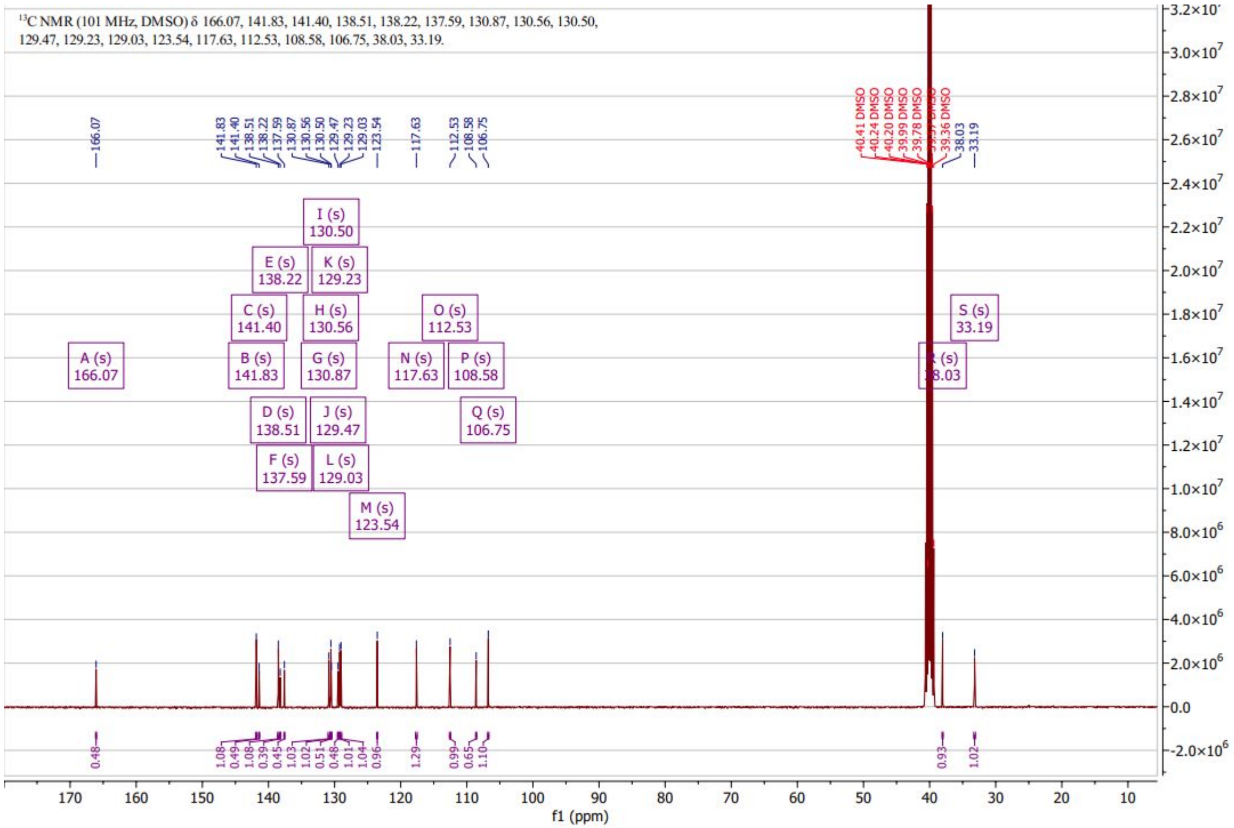

^{1}H NMR Spectra for Compound 9k

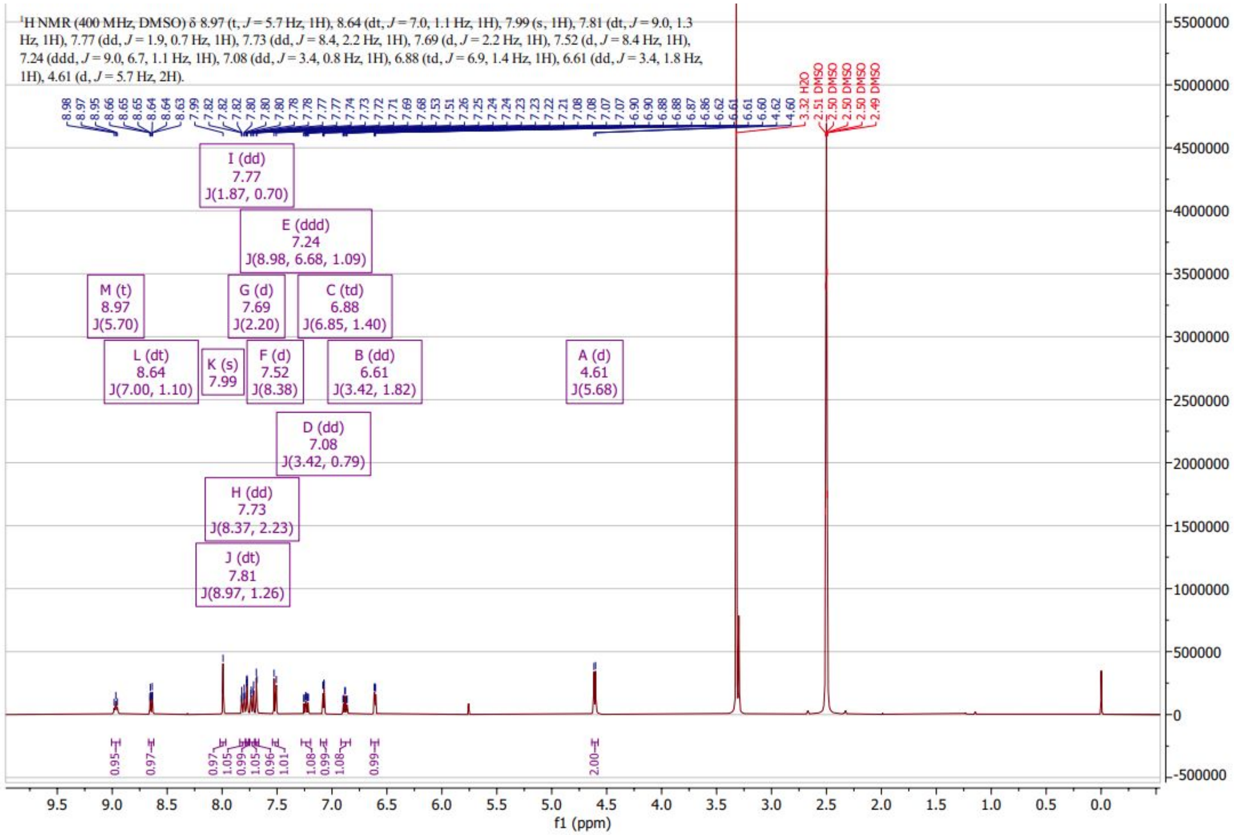

^{13}C NMR Spectra for Compound 9k

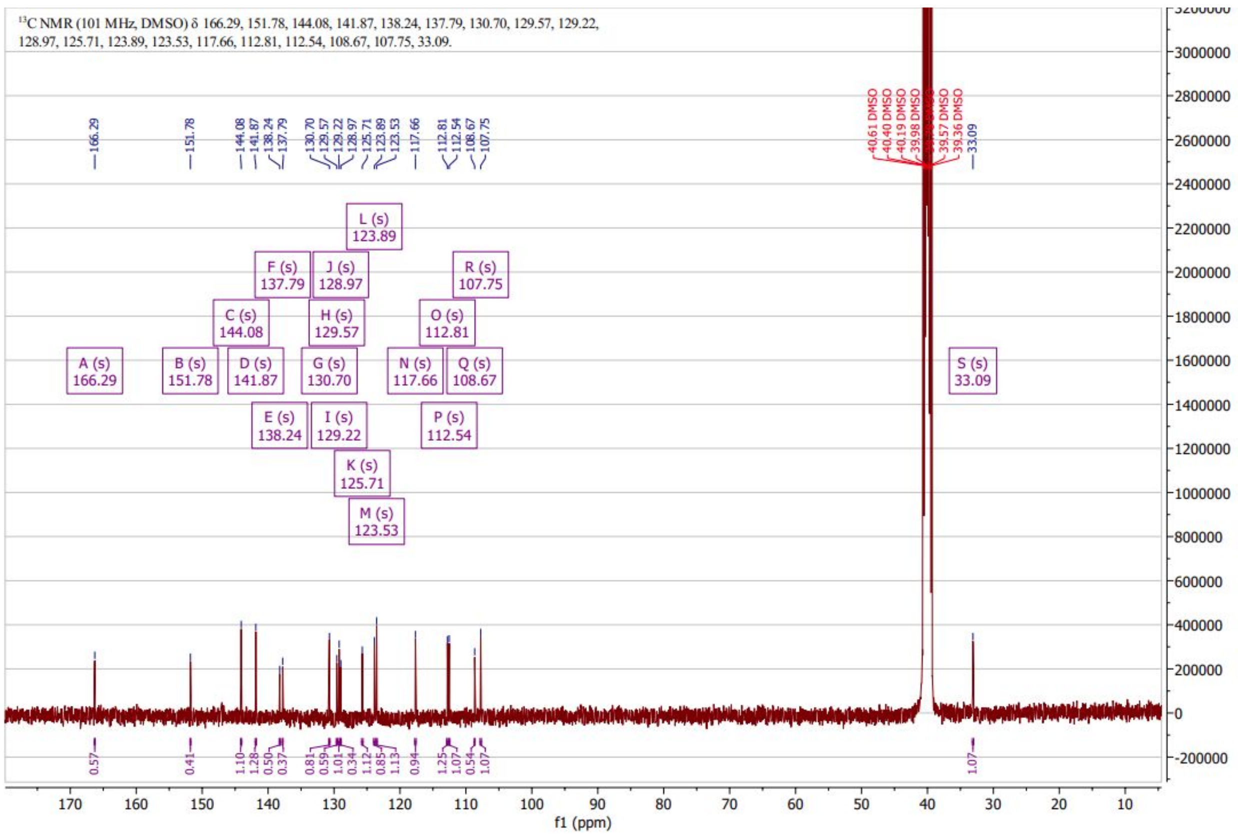

^{1}H NMR Spectra for Compound 15a

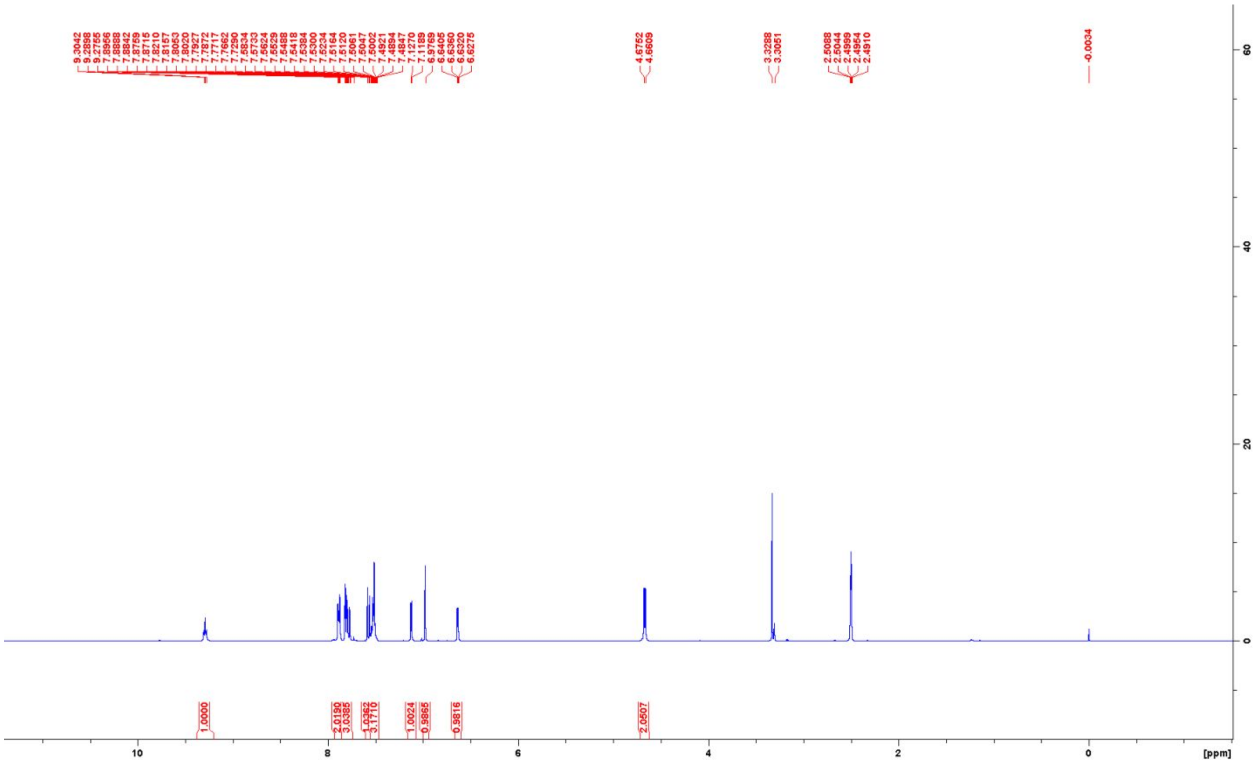

^{13}C NMR Spectra for Compound 15a

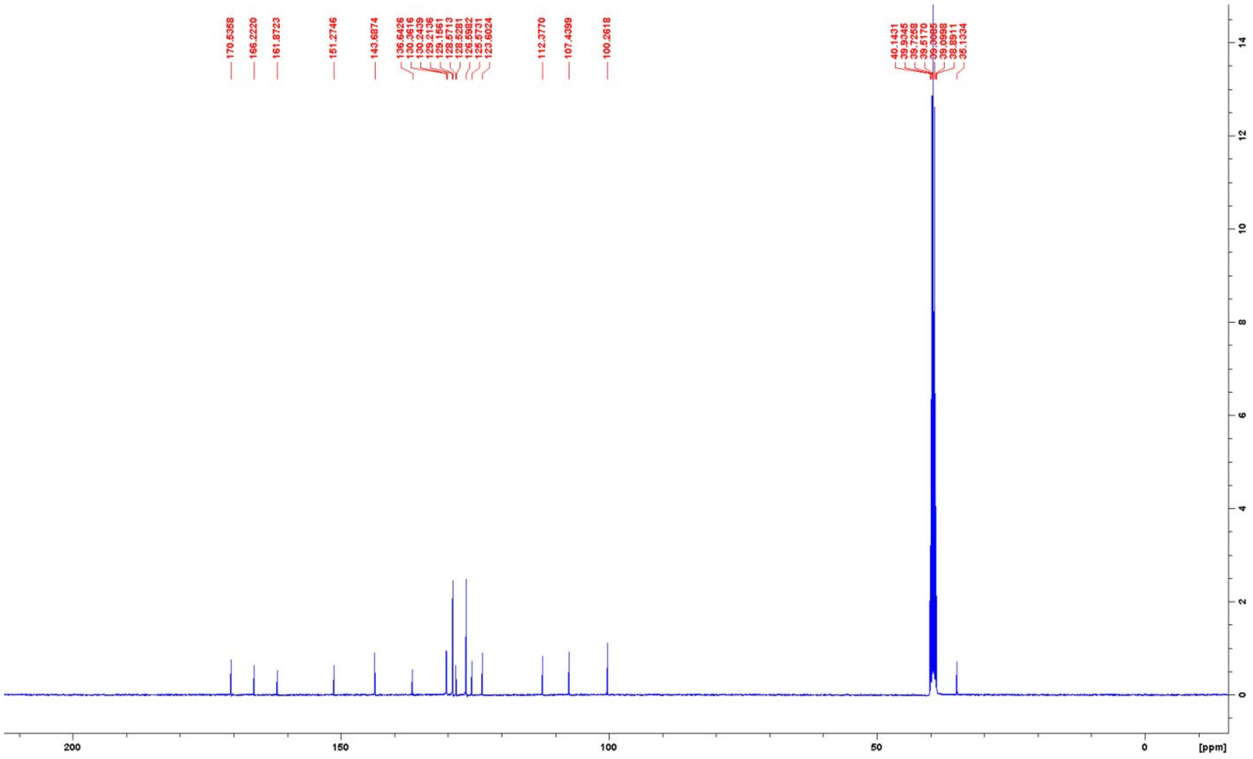

^{1}H NMR Spectra for Compound 15b

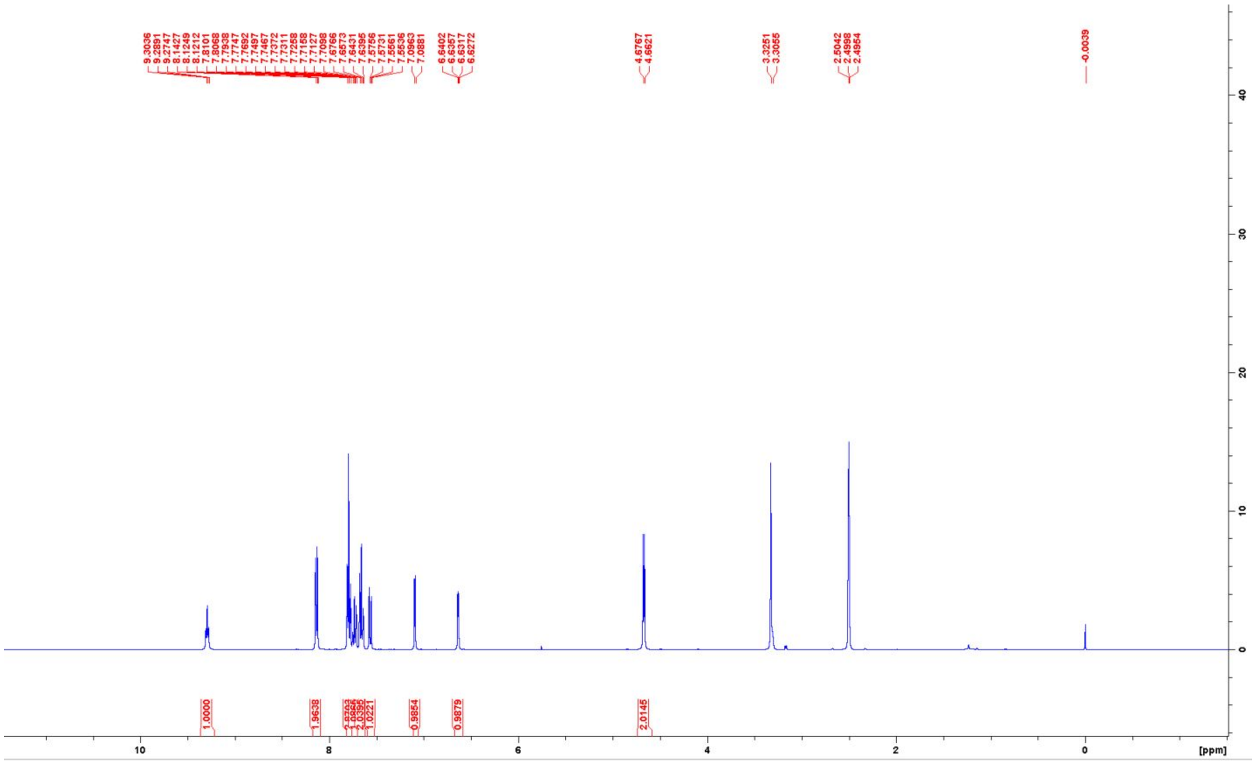

^{13}C NMR Spectra for Compound 15b

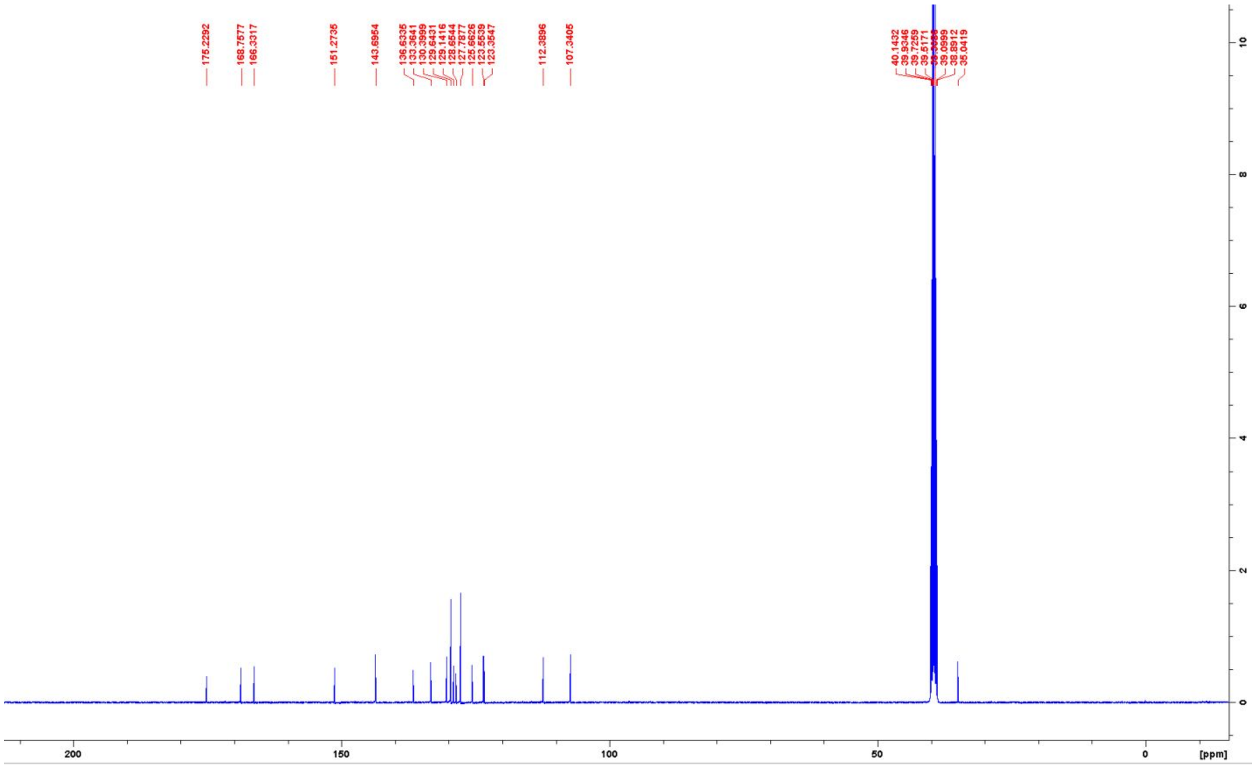

^{1}H NMR Spectra for Compound 15c

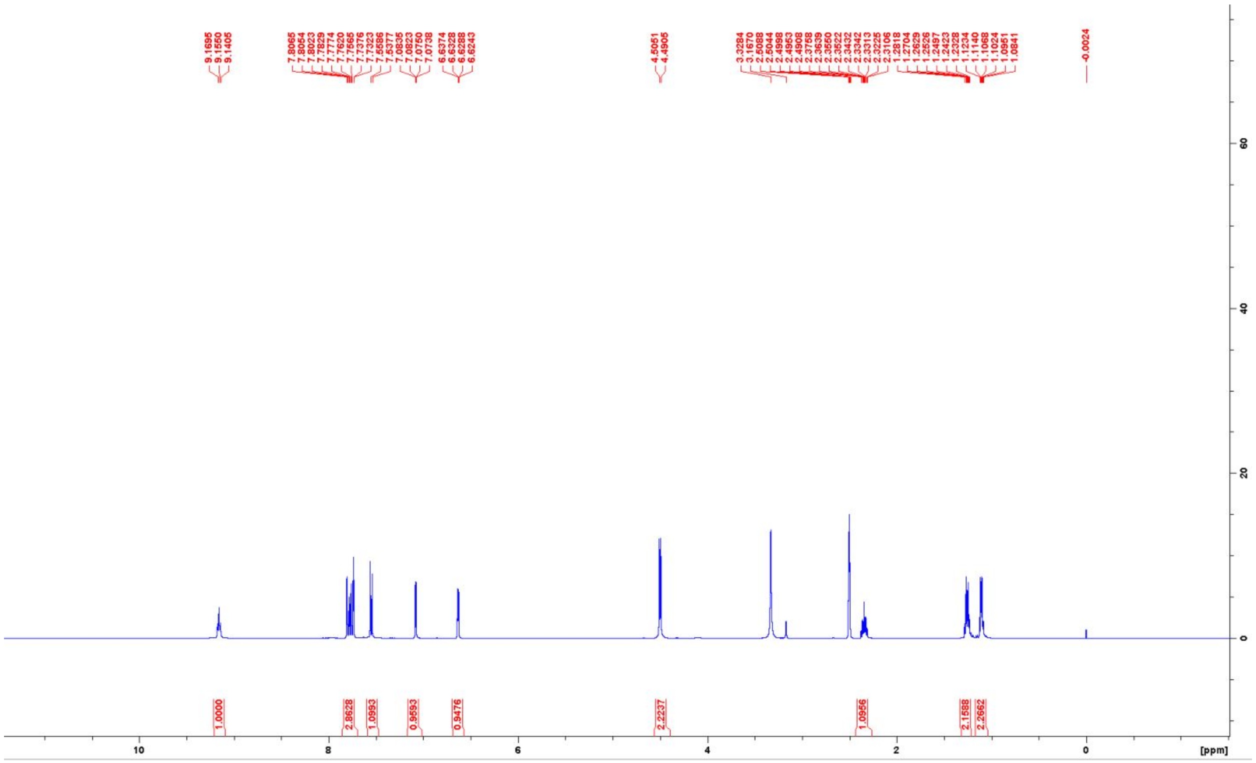

^{13}C NMR Spectra for Compound 15c

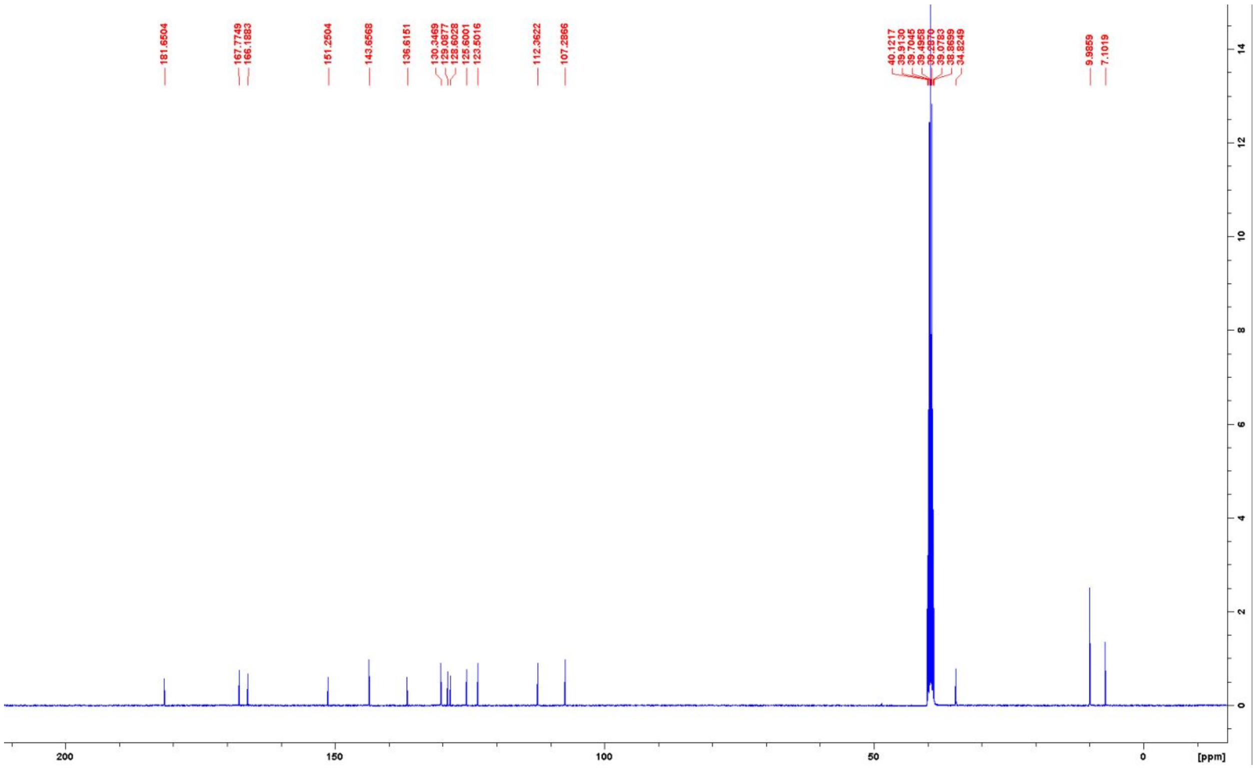

^{1}H NMR Spectra for Compound 15d

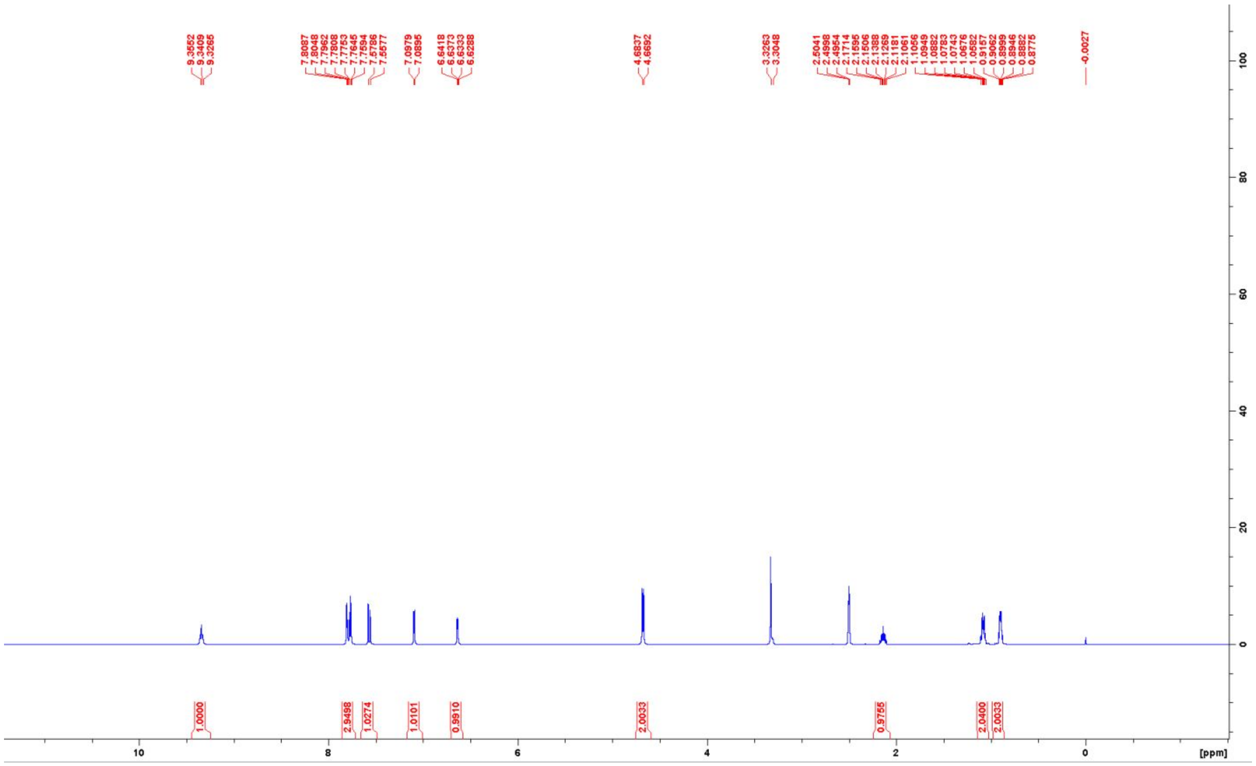

^{13}C NMR Spectra for Compound 15d

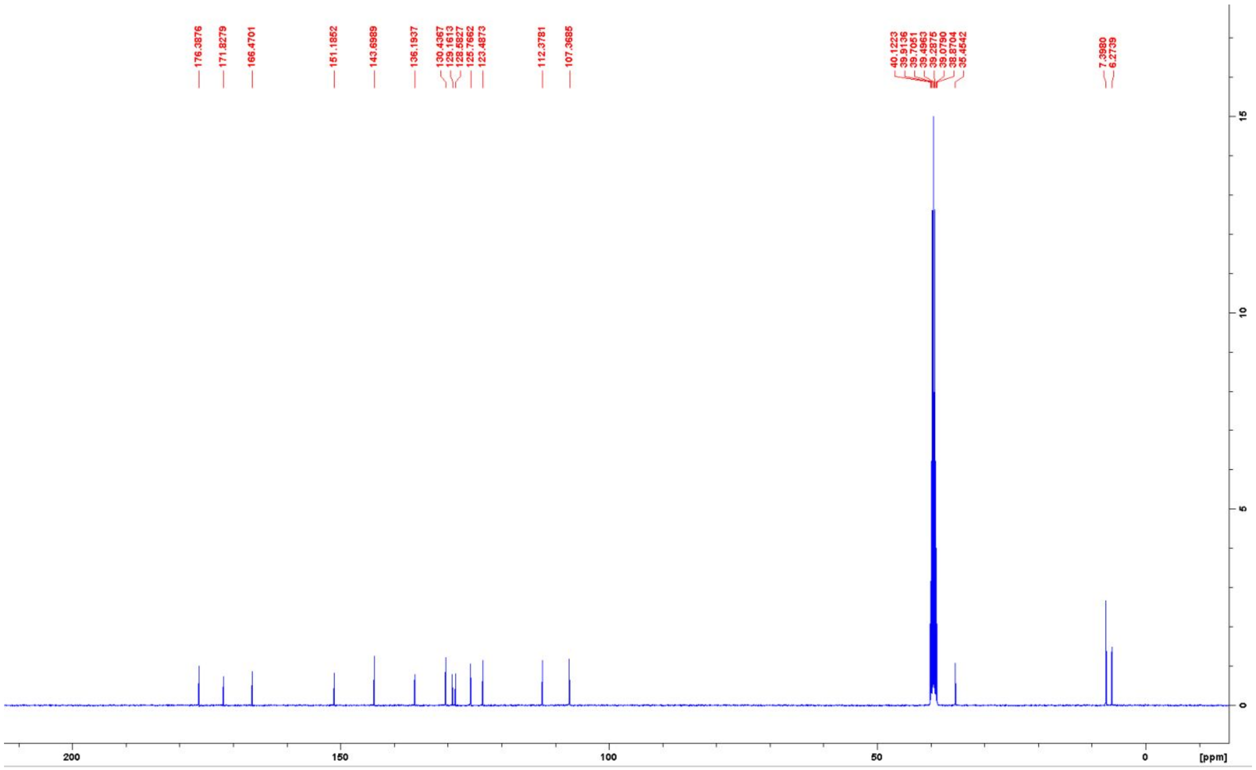

^{1}H NMR Spectra for Compound 15e

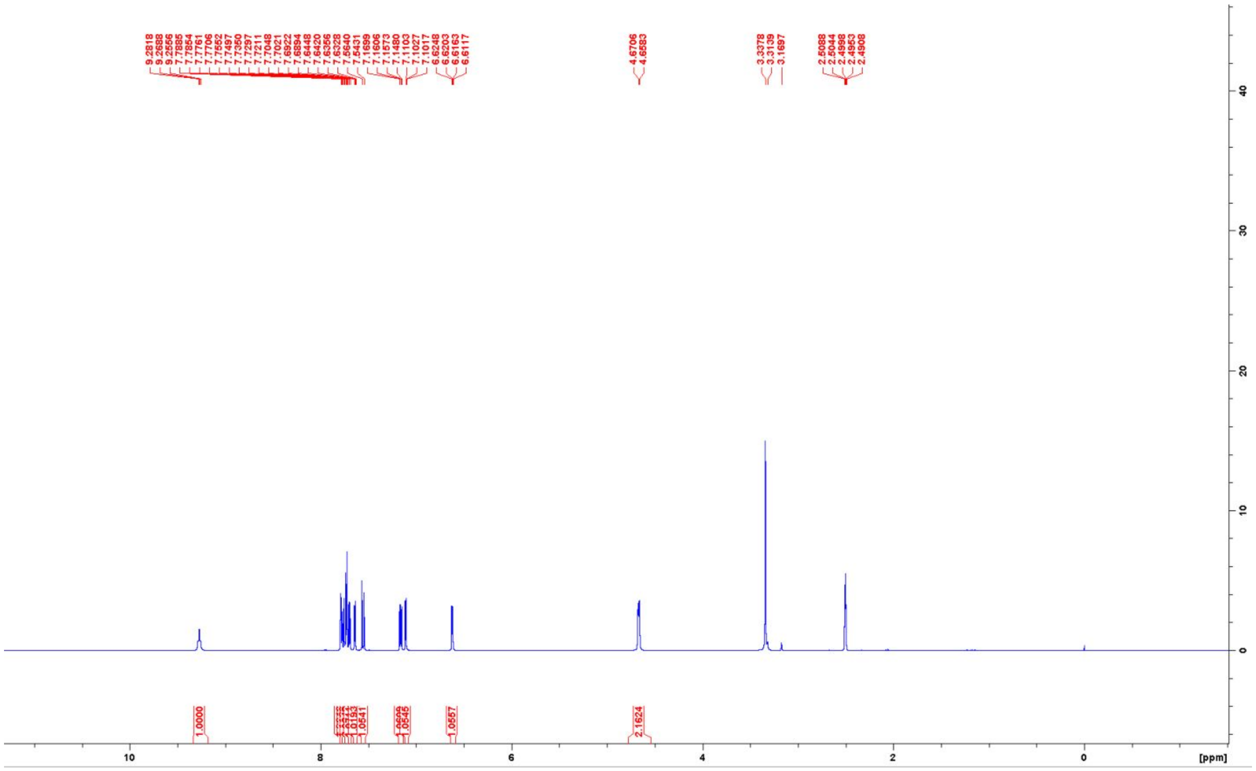

^{13}C NMR Spectra for Compound 15e

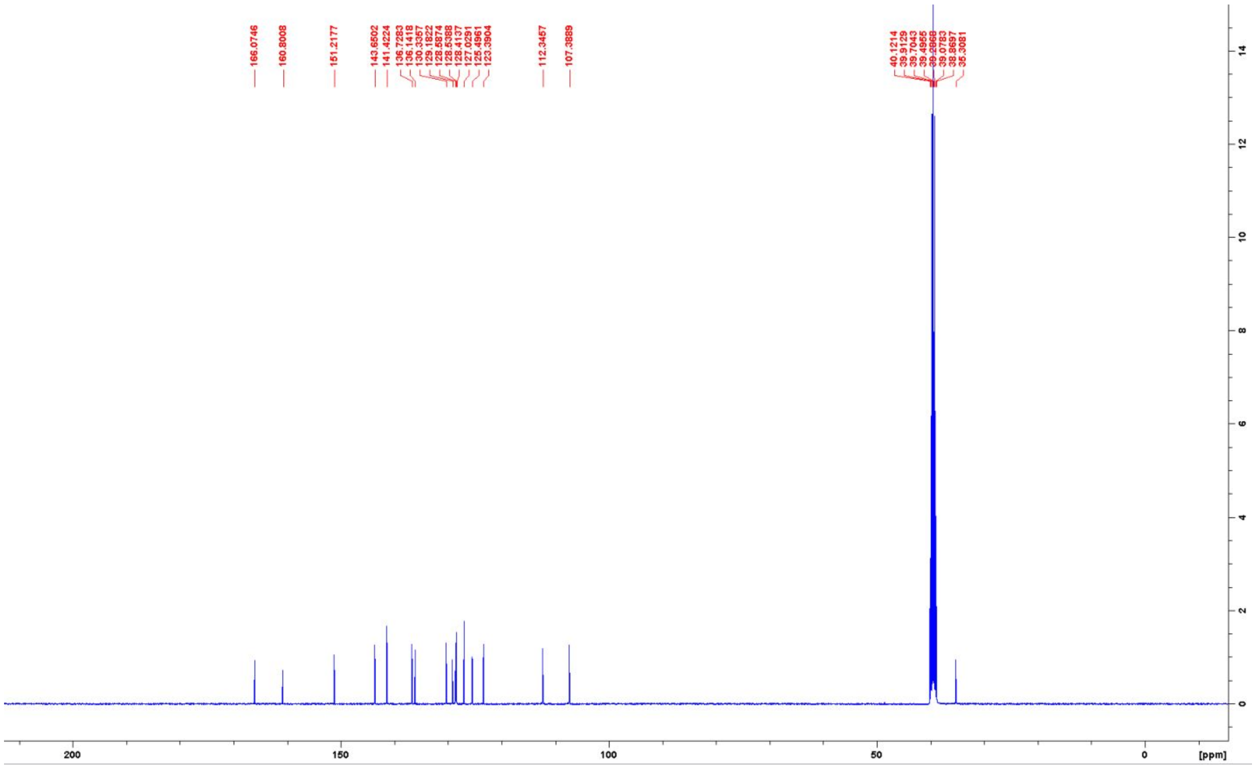

^{1}H NMR Spectra for Compound 15f

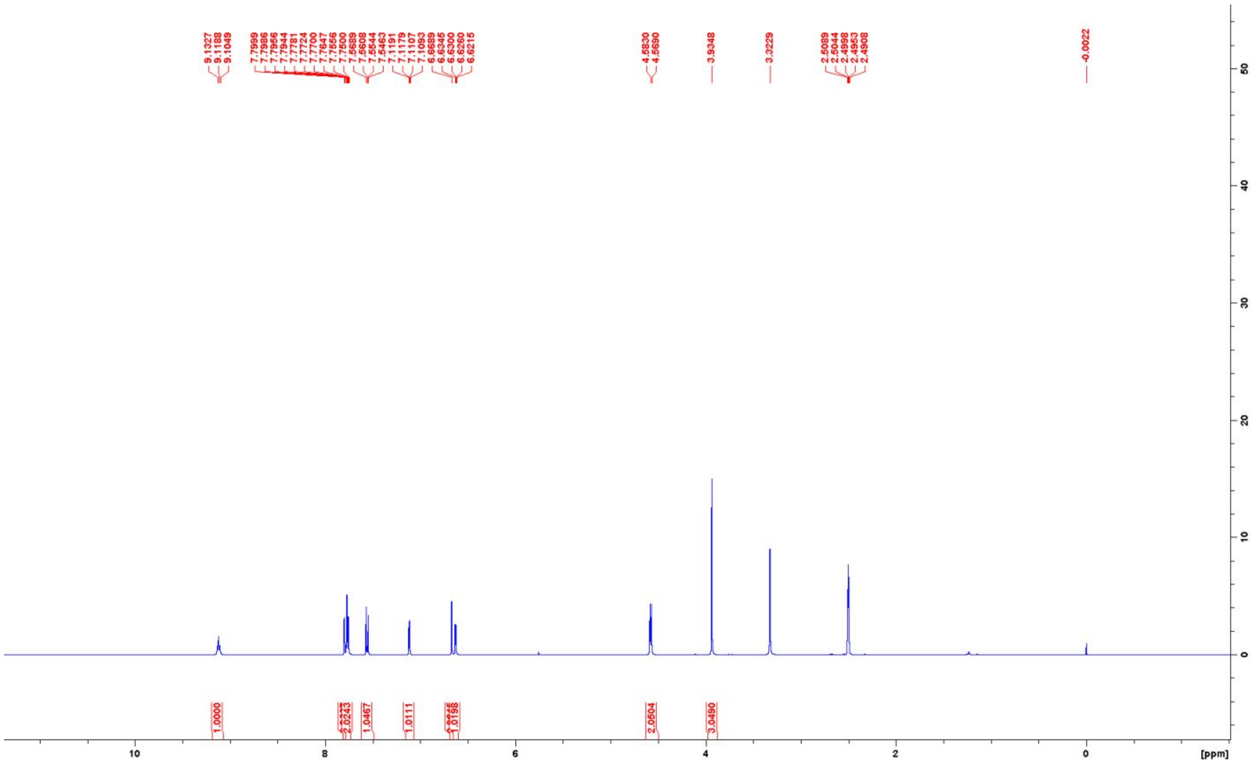

^{13}C NMR Spectra for Compound 15f

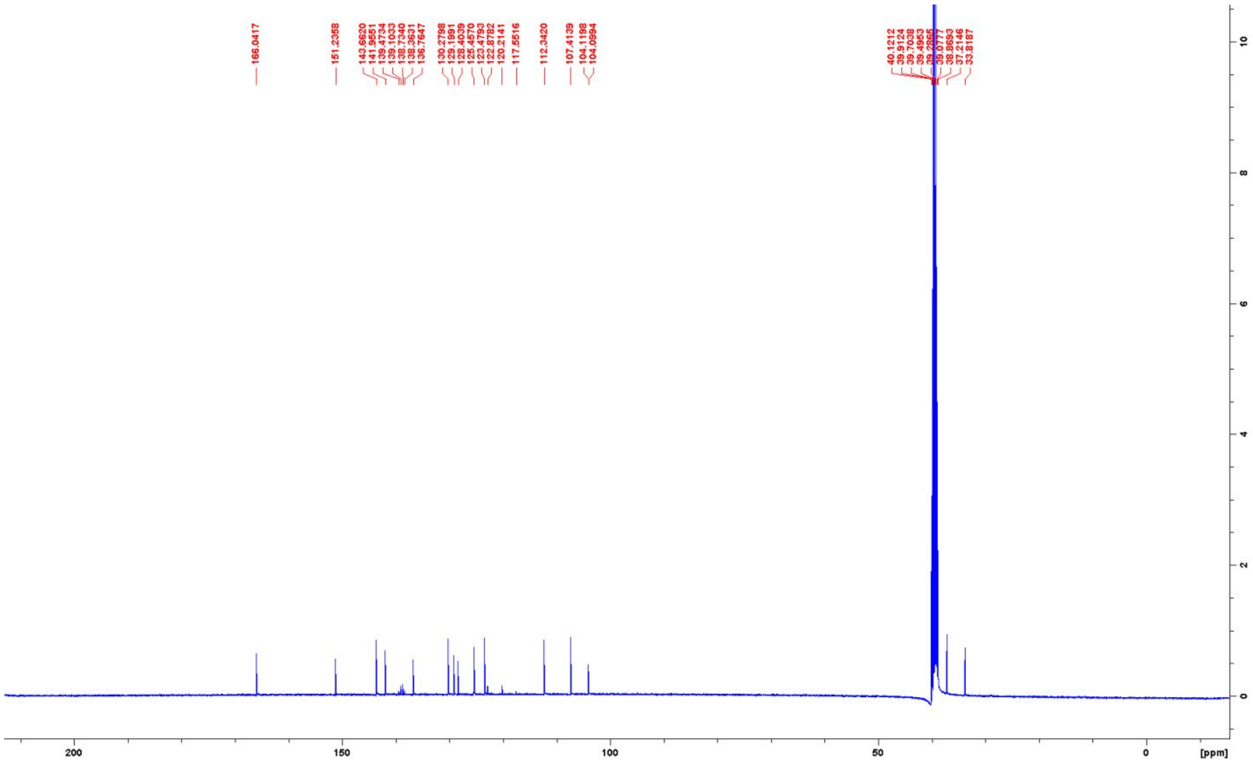

^{1}H NMR Spectra for Compound 19a

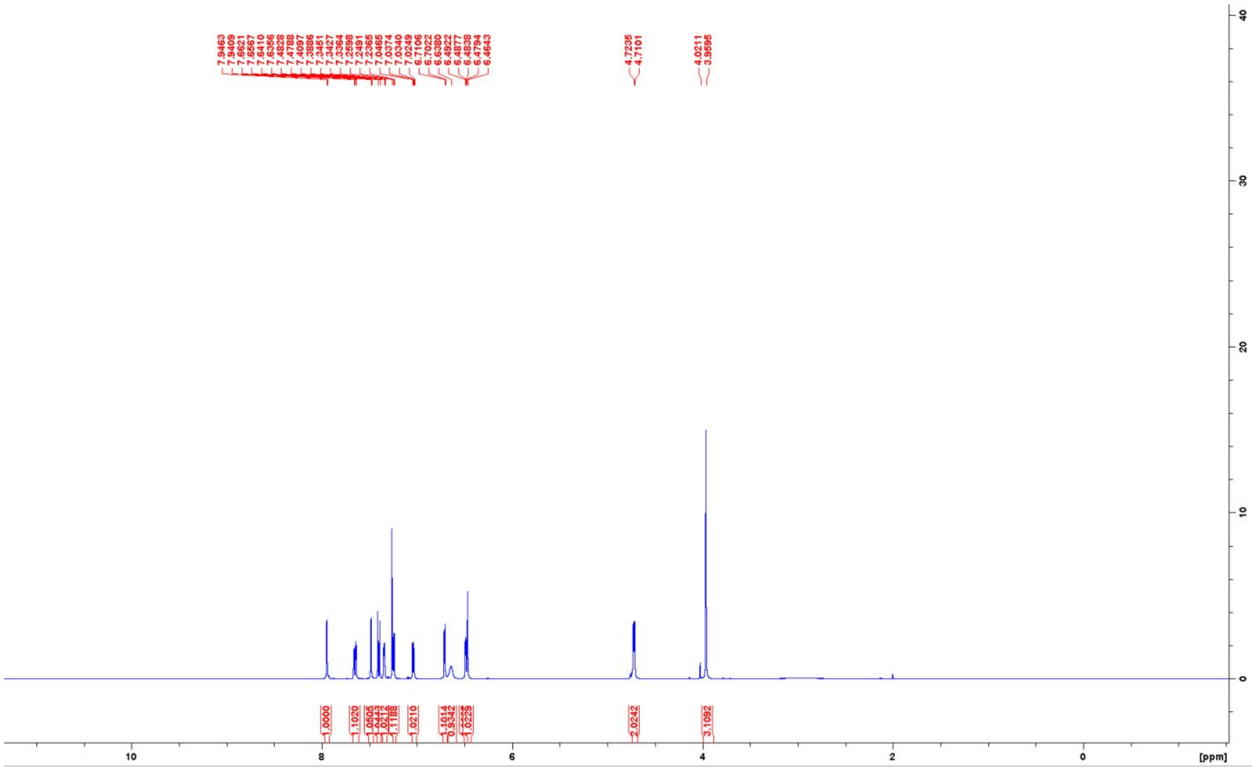

^{13}C NMR Spectra for Compound 19a

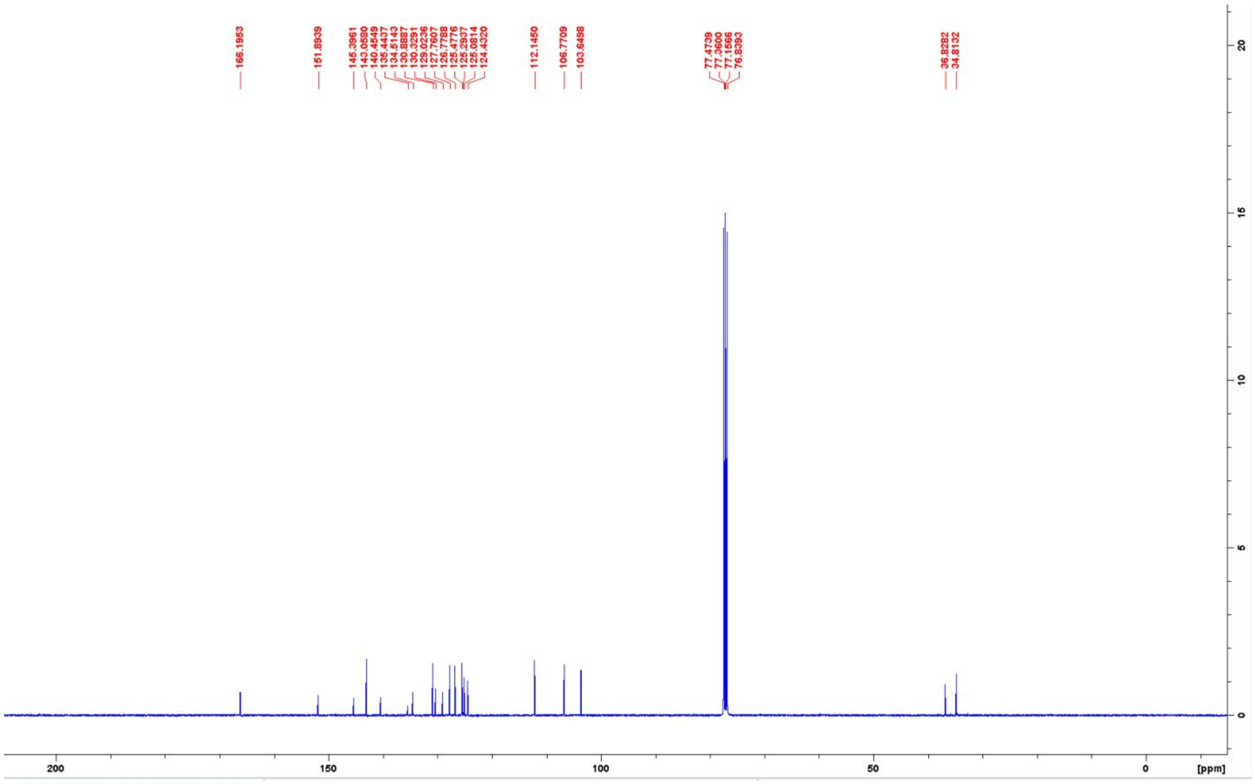

^{1}H NMR Spectra for Compound 19b

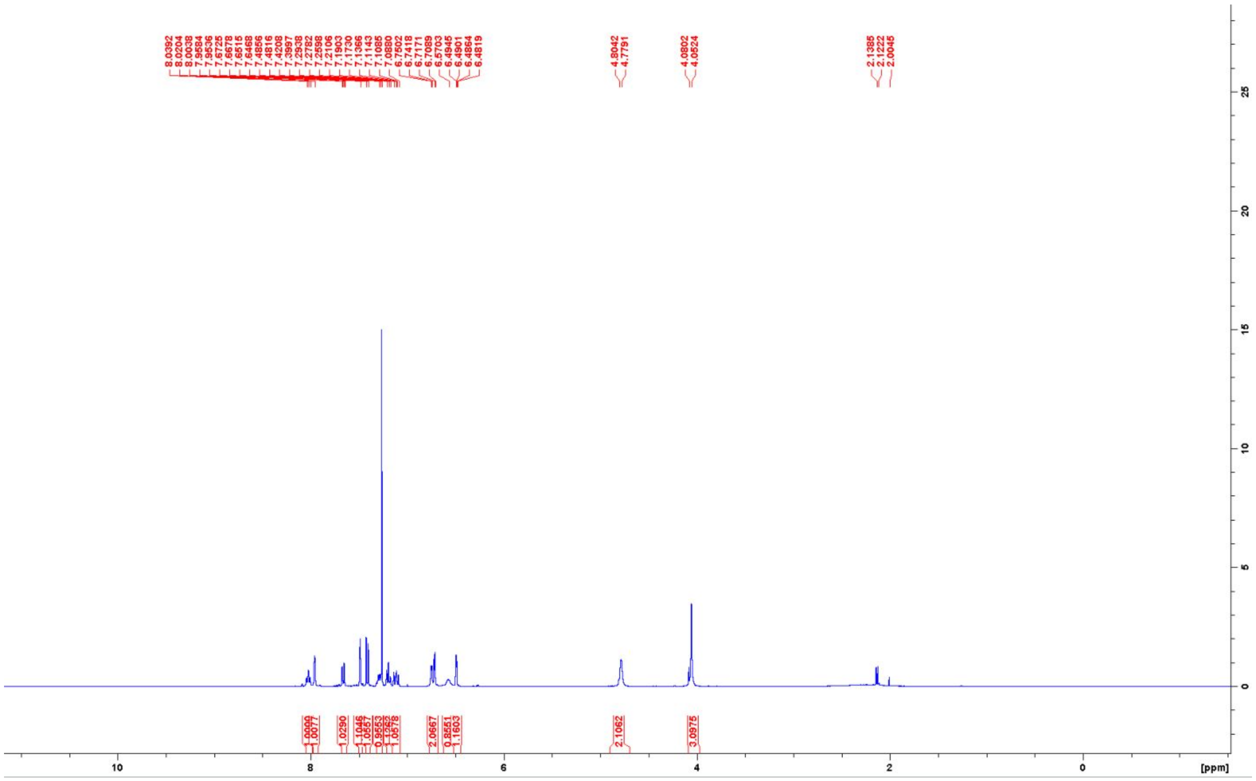

^{13}C NMR Spectra for Compound 19b

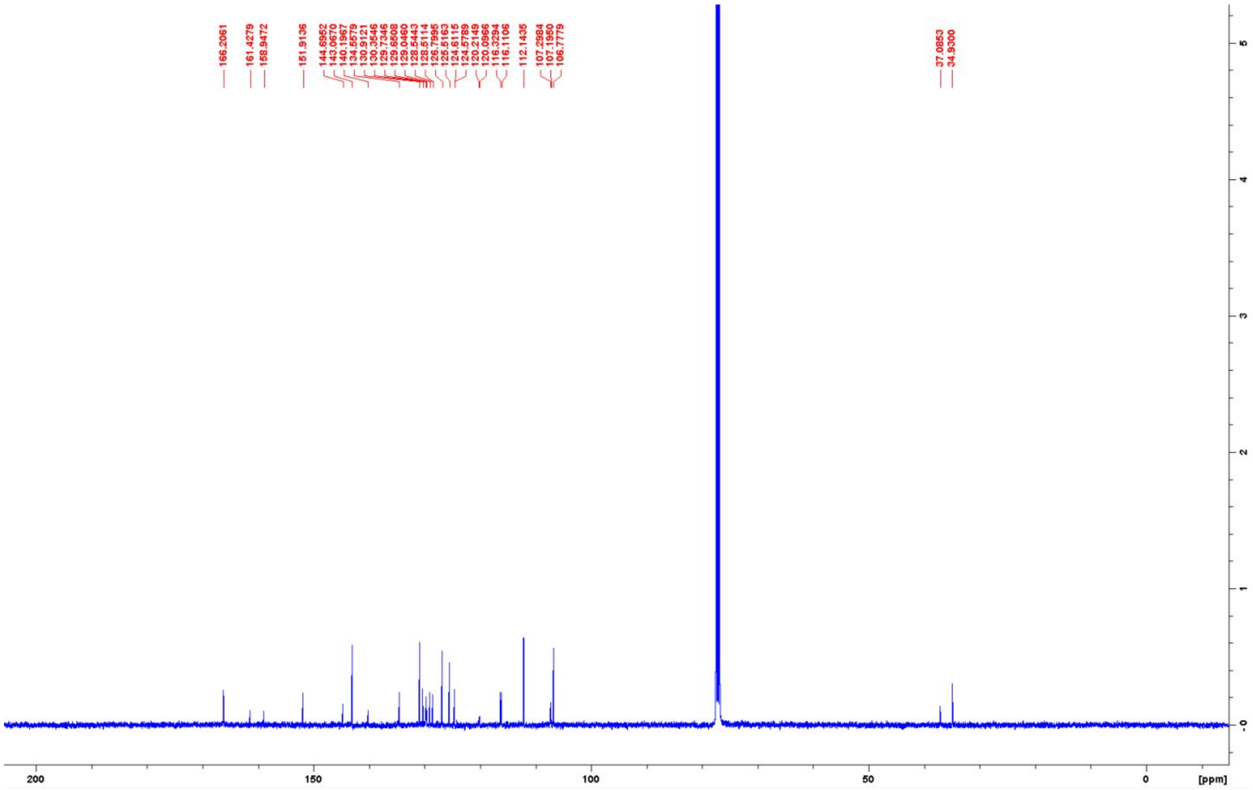

^{1}H NMR Spectra for Compound 19c

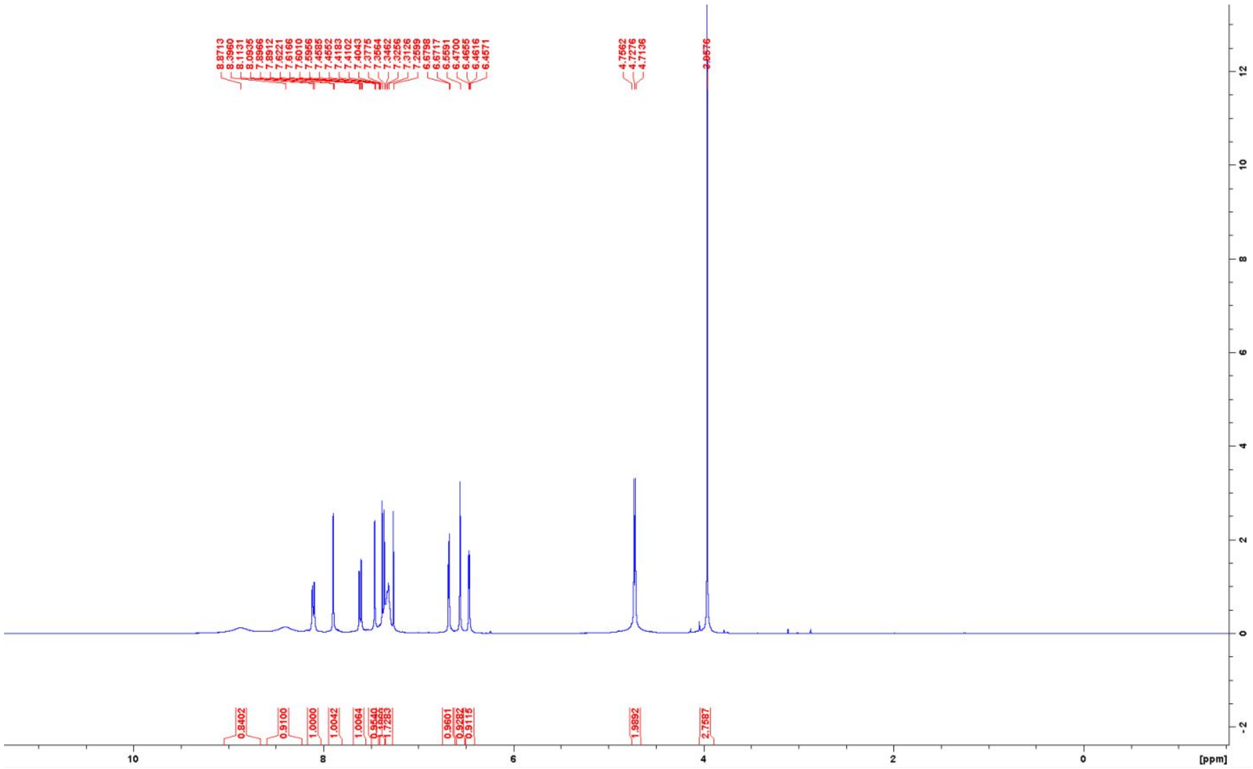

^{13}C NMR Spectra for Compound 19c

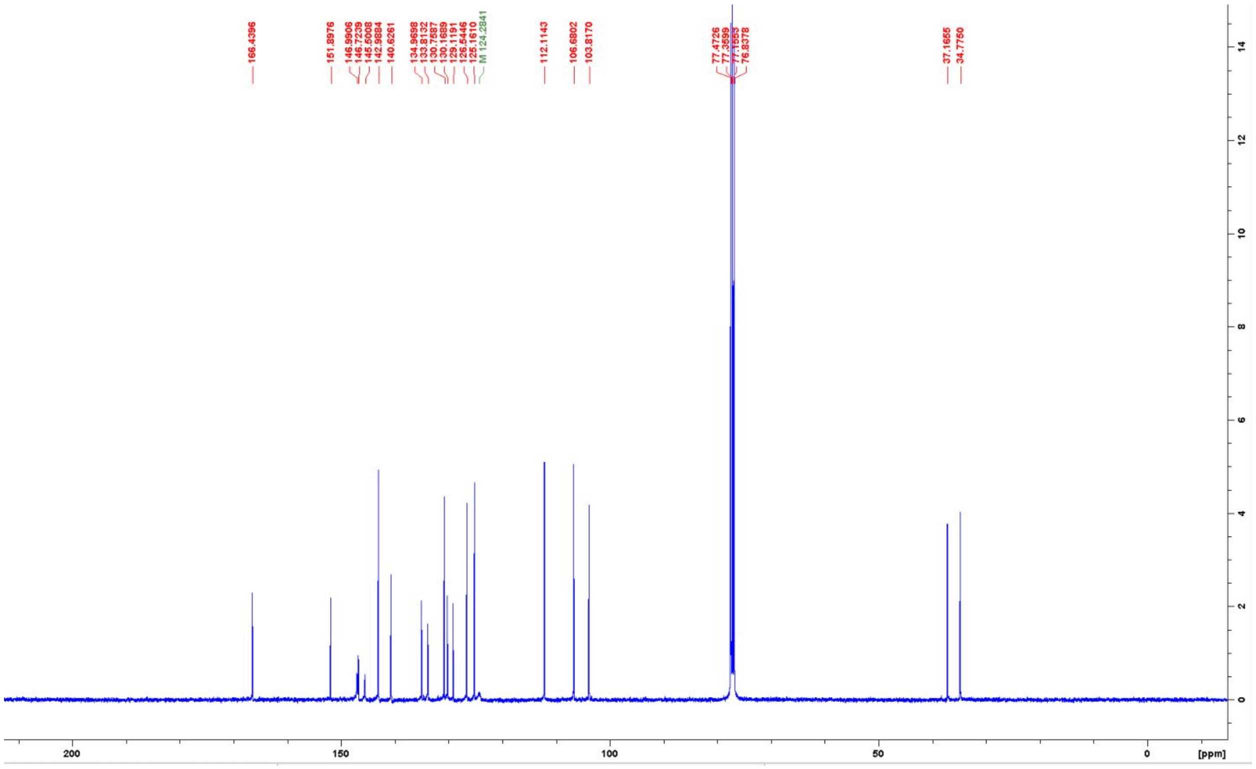

^{1}H NMR Spectra for Compound 19d

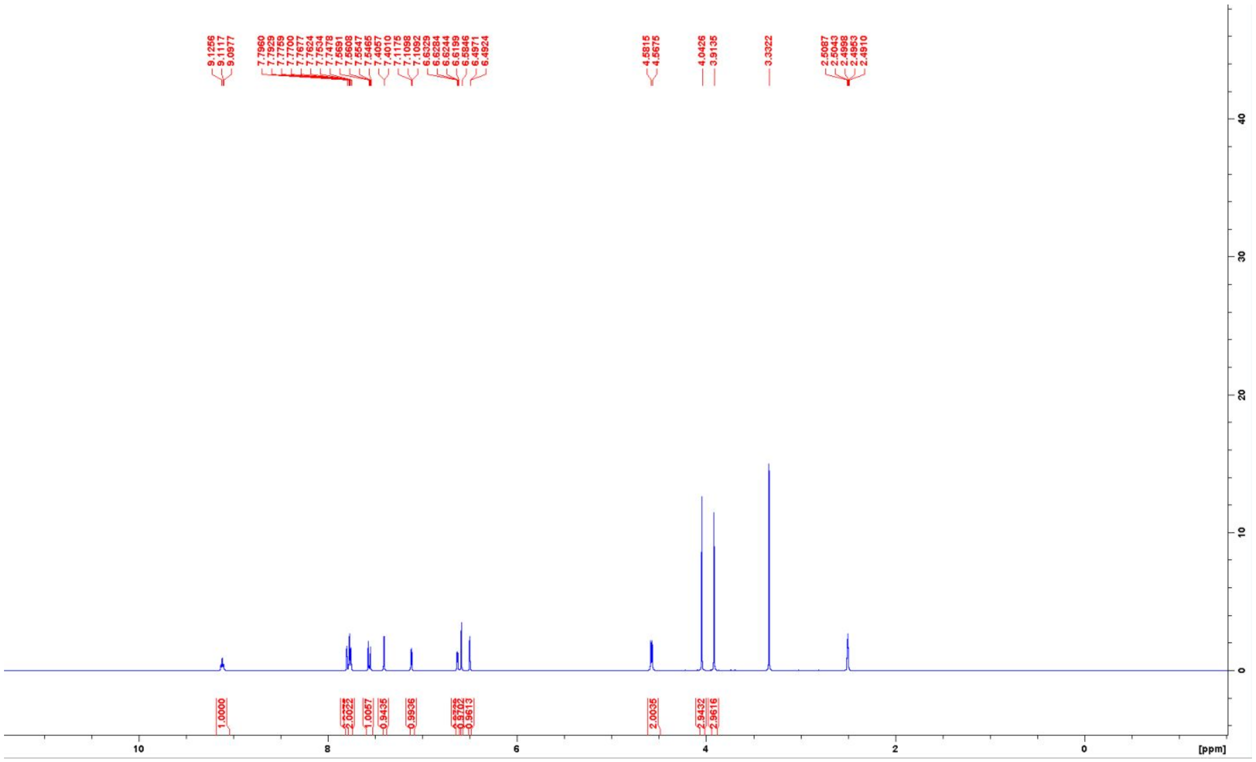

^{13}C NMR Spectra for Compound 19d

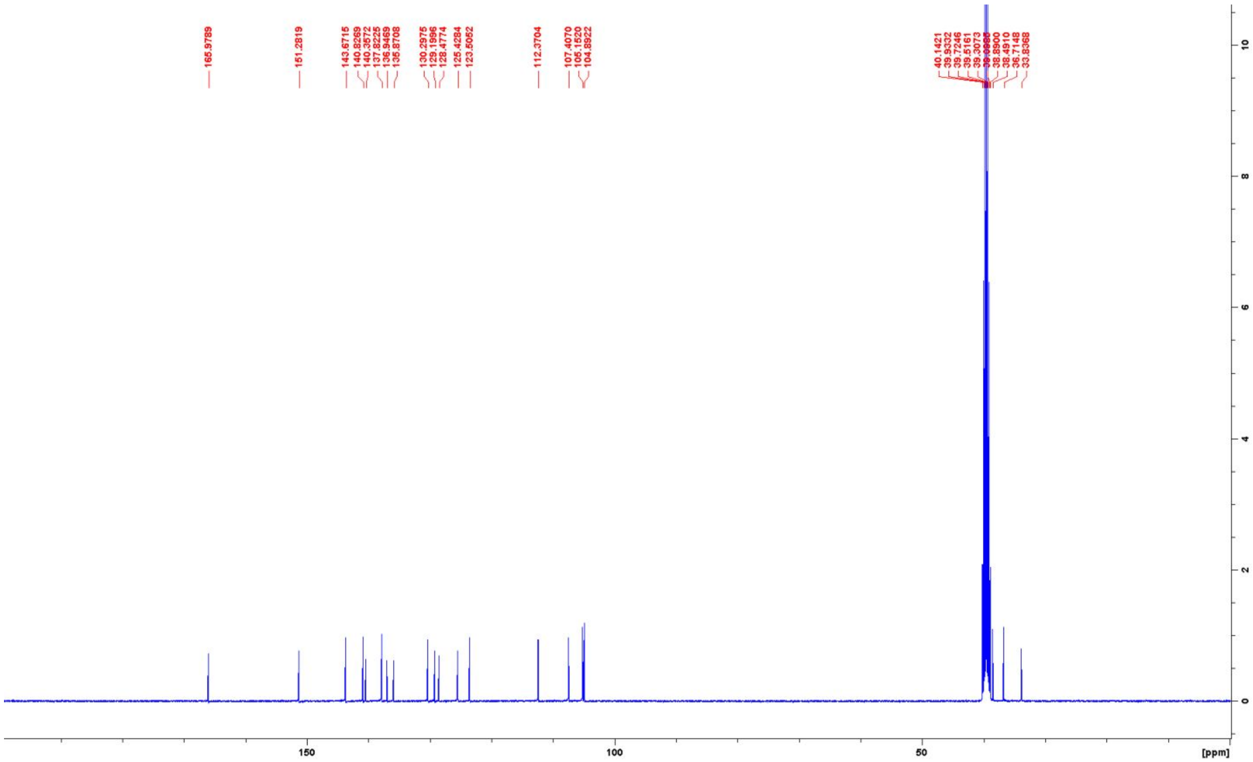

Supplement: Supplementary file 1 — jm4c02554_si_001.pdf [file jm4c02554_si_001.pdf]
